# Cu^I^-Zeolite Catalysis for Biaryl Synthesis via Homocoupling Reactions of Phenols or Aryl Boronic Acids

**DOI:** 10.3390/molecules29235552

**Published:** 2024-11-25

**Authors:** Xiaohui Di, Tony Garnier, Arnaud Clerc, Eliott Jung, Christian Lherbet, Valérie Bénéteau, Patrick Pale, Stefan Chassaing

**Affiliations:** 1 Catalyse Organométallique, Synthèse Organique et Santé (COSyS), Institut de Chimie (UMR-CNRS 7177), Université de Strasbourg, 67000 Strasbourg, France; 2 LSPCMIB, UMR CNRS UPS 5068, Université Toulouse 3 Paul Sabatier, 118 Route de Narbonne, 31062 Toulouse cedex 9, France

**Keywords:** biaryl, biphenols, zeolites, copper, catalysis, coupling

## Abstract

Due to the importance of biaryls as natural products, drugs, agrochemicals, dyes, or organic electronic materials, a green alternative biaryl synthesis has been developed based on easy-to-prepare and cheap copper(I)-exchanged zeolite catalysts. Cu^I^-USY proved to efficiently catalyze the direct homocoupling of either phenols or aryl boronic acids under simple and practical conditions. The Cu^I^-USY-catalyzed oxidative homocoupling of phenols could conveniently be performed under air either in warm methanol or water with good to high yields. In methanol, a small amount of Cs_2_CO_3_ was required, while none was necessary in water. The homocoupling of aryl boronic acids was best performed also in warm methanol, without an additive. These mild conditions showed good functional-group tolerance, leading to a variety of substituted (hetero)biaryls (28 examples). The heterogeneous Cu^I^-USY catalyst could readily be recovered and reused. Interestingly, the homocoupling of vinyl boronic acids was successfully coupled to a Diels–Alder reaction, even in a one-pot process, allowing access to highly functionalized cyclohexenes.

## 1. Introduction

**Figure 1:**
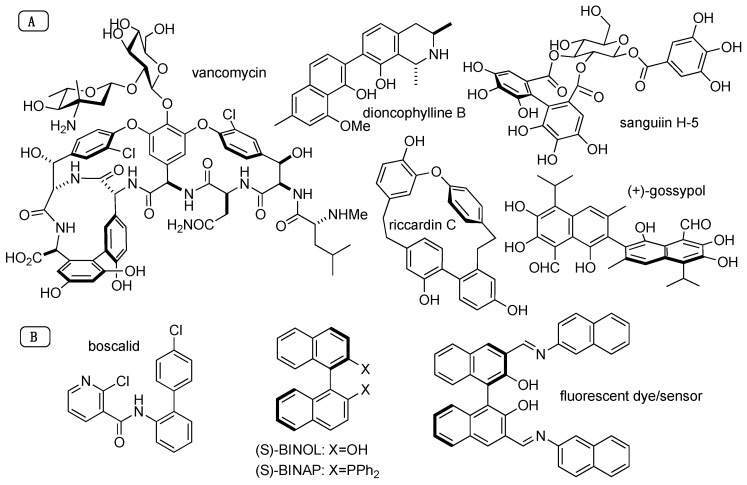
Representative examples of natural (**A**, top) and non-natural (**B**, bottom) biaryl-containing molecules.

Known since the early works of Sandmeyer [1,2], Pschorr [3], and, a few years later, Ullmann [4], the biaryl motif is encountered in numerous natural products (Figure 1A). It can be found in secondary metabolites of various organisms. The most typical of these metabolites are the vancomycin family of antibiotics, well known for their ability to treat infections caused by bacteria that are resistant to other drugs; vancomycin has thus now been on the WHO’s list of essential medicines since 2017 [5,6]. The biaryl motif is also present in various plants in the form (i) of alkaloids, such as potent antimalarial dioncophyllines B and C [7,8]; (ii) of polyphenols, such as pluripotent riccardin C [9] and the ellagitannins sanguiins [10] and tellimagrandins [11]; or (iii) of toxic substances, such as gossypol [12] isolated from cotton seeds. 

This motif is also found in many non-natural molecules including pharmaceuticals, such as highly antihypertensive sartans [13], and agrochemicals, such as boscalid [14] (Figure 1B). While a biaryl unit is the core of various chiral ligands/catalysts [15], dyes, and liquid crystals [16], it has also been employed in various technical applications/materials, e.g., as solar cell components, as organic electronic materials [17], or in MOF for gas storage [18].

**Scheme 1:**
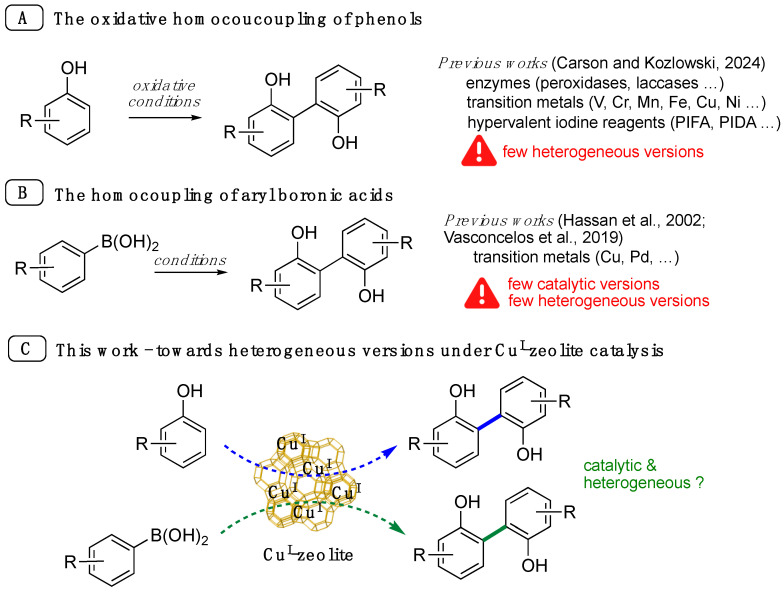
Known methods for biaryl synthesis via oxidative phenol homocoupling (**A**) [21] and aryl boronic acid homocoupling (**B**) [22,26], and the presently proposed alternative based on Cu^I^-zeolite catalysis (**C**).

Because of their relevance to life and materials sciences, biaryls have thus received much attention from synthetic organic chemists. In nature, the preferred synthetic tool for constructing the biaryl bond is the C-C oxidative homocoupling of phenols that is mediated by various enzymes, such as peroxidases, laccases, etc. [19,20]. Therefore, several biomimetic methods based on the use of enzymes, transition metals, and hypervalent iodine reagents have been developed to produce biphenols (Scheme 1A) [21]. The other known syntheses mostly rely on metal-catalyzed homo- or cross-coupling of aryl halides, first in Ullmann-type reactions mediated by copper under harsh conditions [4], then through the now classical palladium-catalyzed reactions under milder conditions [22]. The more recent and concomitant discovery by Chan-Lam and Evans’ groups that aryl boronic acids can also be coupled in the presence of copper(II) salts [23,24] has gained interest, mostly due to the availability of numerous boronic acids and to the mild conditions employed (Scheme 1B) [25]. Homocoupling was first observed as a side reaction in these reactions, hence providing a rapid and mild alternative route to symmetrical biaryls. As a result, several catalysts, mostly based on palladium, have been developed to facilitate the homocoupling of aryl boronic acids, but they usually relied on specific, complex ligands [26]. The use of silver, gold, rhodium, and copper as promoters has also emerged, but to a much lesser extent than palladium [26,27]. Noteworthy is that in the case of copper, most examples required a stoichiometric amount of copper and base.

In contrast, not many heterogeneous versions of these known methods have so far been developed, despite the inherent advantages of easy separation, better handling characteristics, and recyclability exhibited by heterogeneous catalysts [28,29,30,31,32,33,34]. Moreover, only a few methods use inexpensive, earth-abundant copper as catalytically active metal and none of them rely on the use of inexpensive zeolites as solid supports.

On these bases, and due to the current environmental and greening context, we embarked on the synthesis of biaryls using copper(I)-loaded zeolites as catalysts (Scheme 1C). Besides those mentioned above, one advantage of applying zeolites as heterogeneous catalysts is to take benefit from the nanometric size and shape of their internal cavities [35,36]. Zeolites are indeed well known for their selectivity in some petrochemical processes, notably in xylene production [37], and now in zeolite-catalyzed organic reactions [38,39,40], especially in changing the stereoselectivity of some reactions and in limiting the oligomerization of others. We thus explore here the catalytic potential of Cu^I^-USY [38,39], our powerful ‘home-made’ zeolite-based material, in the synthesis of biaryls via (i) the C-C oxidative homocoupling of phenols, and (ii) the C-C homocoupling of aryl boronic acids. Our aim is also to develop conditions that are as mild and green as possible.

## 2. Results and Discussion

In our previous works, Cu^I^-loaded zeolites of the faujasite type, especially Cu^I^-USY, proved to be the best catalyst [38,39,41,42,43,44]. The latter is obtained by H^+^ to Cu^I^ exchange from its parent acidic zeolite (H-USY) and can be readily characterized [45,46]. Therefore, we first looked for coupling conditions involving Cu^I^-USY as a heterogeneous catalyst.

### 2.1. Biaryl Synthesis via Cu^I^-USY Oxidative Phenol Coupling

#### 2.1.1. Setup of Coupling Conditions (Table 1)

To evaluate the catalytic potential of Cu^I^-zeolites, especially Cu^I^-USY in the oxidative phenol coupling, we selected 2,4-di-*tert*-butylphenol **1a** as a model substrate. When the recently reported homogeneous conditions [47] were transposed to Cu^I^−USY, the reaction performed with **1a** was very slow and only traces of the expected product, **2a**, could be detected (entry 2 vs. 1). Heating and more dilute conditions or a higher catalyst concentration allowed us to reach a significant amount of the coupling product (entries 3–5 vs. 2). While shifting to more polar and/or protic solvents was not beneficial (entries 6–7), methanol nevertheless appeared as a promising alternative solvent for performing the expected coupling (entry 7). Rewardingly, greener solvents and cesium carbonate as a base improved yields, especially in methanol and water (entries 8–9 vs. 7). Halving the quantity of cesium carbonate proved to be profitable (entry 10 vs. 8). Among the bases screened, cesium carbonate proved to be the best base (entry 10 vs. 7, 11–12). Surprisingly, the base was required in methanol but not in water (entry 13 vs. 14). Further control experiments showed that no reaction took place with native zeolite or without a catalyst, in both methanol and water (entries 15–20).

**Table 1:**
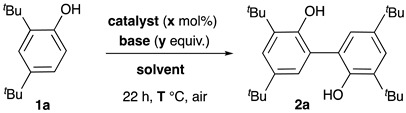
Screening of reaction conditions for the Cu^I^-USY-catalyzed oxidative phenol coupling of 2,4-di-*tert*-butylphenol **1a** ^a^.

Entry ^a^	Catalyst (x mol%)	Base (y equiv.)	Solvent	T (°C)	Yield ^b^ (%)
1 ^c,d^	CuCl (10)	NEt_3_ (0.5)	CH_2_Cl_2_	rt	79
2 ^d^	Cu^I^-USY (10)	NEt_3_ (0.5)	CH_2_Cl_2_	rt	traces ^e^
3 ^d^	Cu^I^-USY (10)	NEt_3_ (0.5)	CH_2_Cl_2_	90 ^f^	42
4	Cu^I^-USY (10)	NEt_3_ (0.5)	CH_2_Cl_2_	90 ^f^	48
5	Cu^I^-USY (15)	NEt_3_ (0.5)	CH_2_Cl_2_	90 ^f^	55
6	Cu^I^-USY (15)	NEt_3_ (0.5)	DMF	100	traces ^e^
7	Cu^I^-USY (15)	NEt_3_ (0.5)	MeOH	65	35 ^e^
8	Cu^I^-USY (15)	Cs_2_CO_3_ (0.5)	MeOH	65	65 ^e^
9	Cu^I^-USY (15)	Cs_2_CO_3_ (0.5)	H_2_O	100	63 ^e^
**10**	**Cu^I^-USY (15)**	**Cs_2_CO_3_ (0.25)**	**MeOH**	**65**	**70**
11	Cu^I^-USY (15)	K_2_CO_3_ (0.25)	MeOH	65	35 ^e^
12	Cu^I^-USY (15)	K_3_PO_4_ (0.25)	MeOH	65	17 ^e^
13	Cu^I^-USY (15)	-	MeOH	65	traces ^e^
**14**	**Cu^I^-USY (15)**	**-**	**H_2_O**	**100**	**65**
15	H-USY (15)	Cs_2_CO_3_ (0.25)	MeOH	65	traces
16	NH_4_-USY (15)	Cs_2_CO_3_ (0.25)	MeOH	65	traces
17	none	Cs_2_CO_3_ (0.25)	MeOH	65	traces
18	H-USY (15)	-	H_2_O	100	traces
19	NH_4_-USY (15)	-	H_2_O	100	traces
20	none	-	H_2_O	100	traces

^a^ Reactions run for 22 h under air with 2,4-di-tert-butylphenol (1 M concentration) unless otherwise stated. ^b^ Yield of isolated pure product. ^c^ Homogeneous control conditions like previously developed conditions. See ref. [43] for details. ^d^ Reaction run under air with 2,4-di-tert-butylphenol at 0.1 M concentration. ^e^ Incomplete conversion. ^f^ Reaction run in sealed tube. Bold lines (10, 14) highlighted the best results under each condition.

#### 2.1.2. Substrate Scope and Limitations

**Scheme 2:**
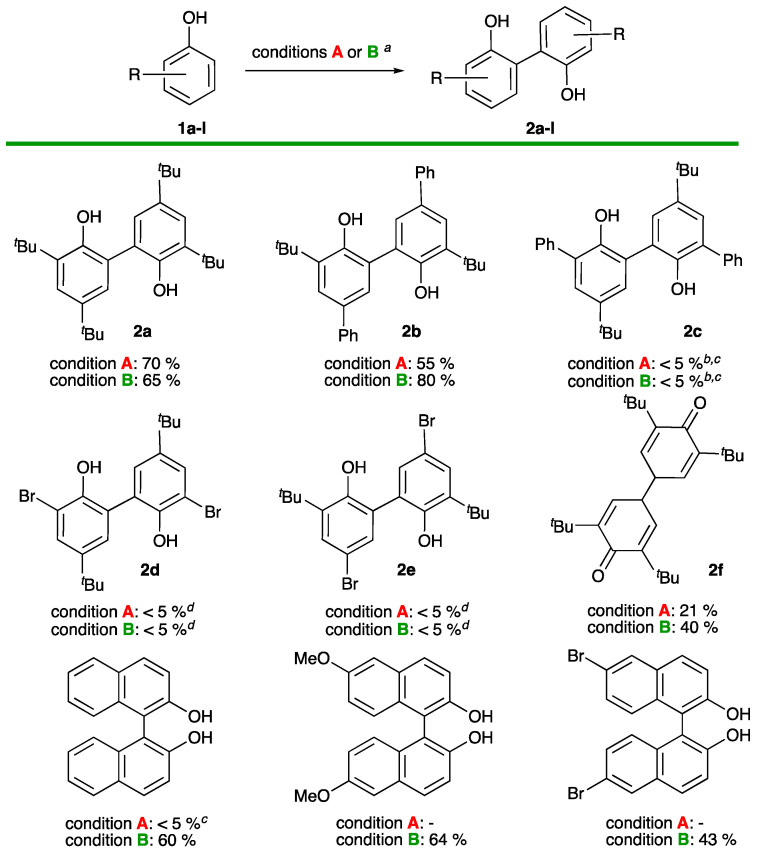
The scope of the Cu^I^-USY-catalyzed oxidative-phenol-coupling conditions. *^a^* Condition **A**: reactions run under air in MeOH with Cu^I^-USY (15 mol%), Cs_2_CO_3_ (0.25 equiv.), and a phenolic substrate (1.0 equiv. with a 1 M concentration) at 65 °C during 22 h, or condition **B**: reactions run under air in H_2_O with Cu^I^-USY (15 mol %) and a phenolic substrate (1 M) at 100 °C during 22 h. *^b^* Incomplete conversion. *^c^* The obtention of a complex mixture of products was observed, as revealed by LCMS. *^d^* Traces of the expected product detected by LCMS.

These green conditions were then applied to the coupling of a representative set of phenols (Scheme 2).

Compared to our model phenol, which dimerized to biphenol **2a** with similar efficiency in methanol or water, its 4-phenylated analog gave the expected dimer **2b** with better yield in water but lower yield in methanol. In contrast, its regioisomer led to a mixture of products from which the expected dimer **2c** could be detected in only a low amount among various side products. Some of the latter may correspond to dibenzofuran derivatives, the formation of which being known from *ortho*-phenyl phenols under oxidative conditions [48]. The two brominated analogs behave similarly, providing again a complex mixture in which only low amounts of the expected dimers **2d** and **2e** were detected.

Interestingly, the 2,6-di-*tert*-butylphenol, a regiosiomer of **1a**, provided the expected dimer, but under the sensitive diphenoquinone form **2f**. Due to its fragility, this product could only be isolated in modest yields, with again better results in water.

Rewardingly, 2-naphthol derivatives could also be engaged in this homocoupling, providing the corresponding binaphthols in good yields. The simplest member, 2-naphthol, readily gave the expected dimer **2g** but only cleanly in water. As for some other preceding examples, basic conditions in methanol proved deleterious and only led to a complex mixture of products. The electron-enriched 6-methoxy-2-naphthol reacted slightly more efficiently than its parent β-naphthol compound, furnishing biphenol **2h** in good yield in water. However, the brominated dimeric analog **2i** was obtained in lower yield from 6-bromo-2-naphthol. The latter results revealed some influence of electronic effects upon this oxidative coupling, reminiscent of related coupling in homogeneous conditions.

#### 2.1.3. Cu^I^-USY Catalyst Recovery and Recycling

**Figure 2:**
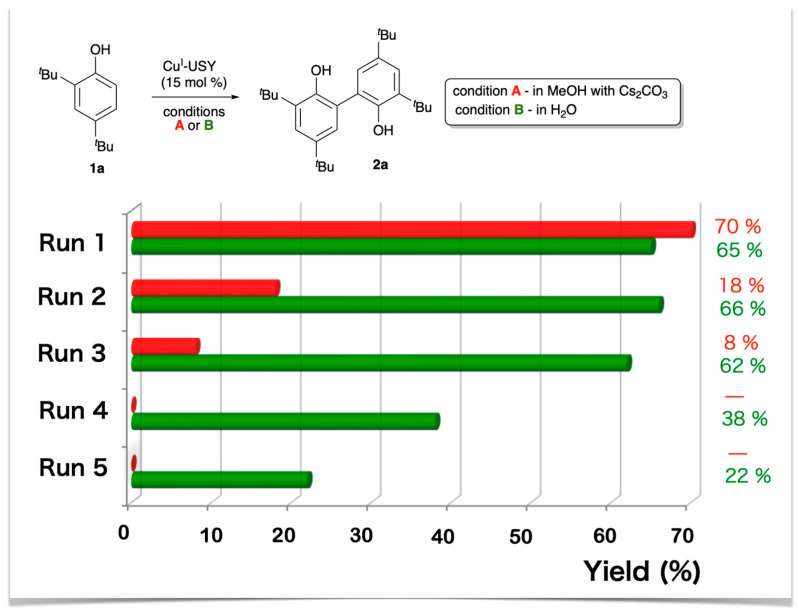
Recycling studies: oxidative phenol coupling of 2,4-di-*tert*-butylphenol **1a** successively catalyzed by the same recovered Cu^I^-USY material under reaction conditions **A** and **B**.

The Cu^I^-USY catalyst recycling and reuse were evaluated under both reaction conditions, **A**/**B**, with the model substrate, i.e., 2,4-di-*tert*-butylphenol **1a** (Scheme 2, Figure 2). After each reaction run, the insoluble materials were filtered, successively washed with water and methanol, dried, and re-engaged in the next run. As can be seen, condition **A** induced deleterious effects on the zeolitic materials, with an important drop in catalytic activity after the first run and a lower one after the second. This could be attributed to the sensitivity of the zeolitic material to basic conditions, leading to its degradation. In sharp contrast, the catalytic activity was maintained for three runs under condition **B**. A substantial decrease nevertheless took place in the fourth run. In that case, we performed a Sheldon test to examine whether leaching of copper active species is occurring or not. After the filtration of the solid material, the test indicated the additional formation of biphenol **2a**, but at a rate by far slower than the Cu^I^-USY-catalyzed reaction. This result reveals the leaching of some active species from the native zeolite catalyst, thus clarifying the decrease observed under condition **B** after the third run.

#### 2.1.4. Mechanistic Scenario

Mechanistic aspects related to the oxidative homocoupling of phenols (or naphthols) have already attracted much attention, especially when performed under copper catalysis [21,49,50]. While various mechanistic scenarios have been reported so far, the archetypal mechanisms are currently those involving phenoxy radicals (or metal-bound surrogates thereof) as key intermediates. These radicals could be generated either by hydrogen radical abstraction or by deprotonation/electron abstraction.

**Scheme 3:**
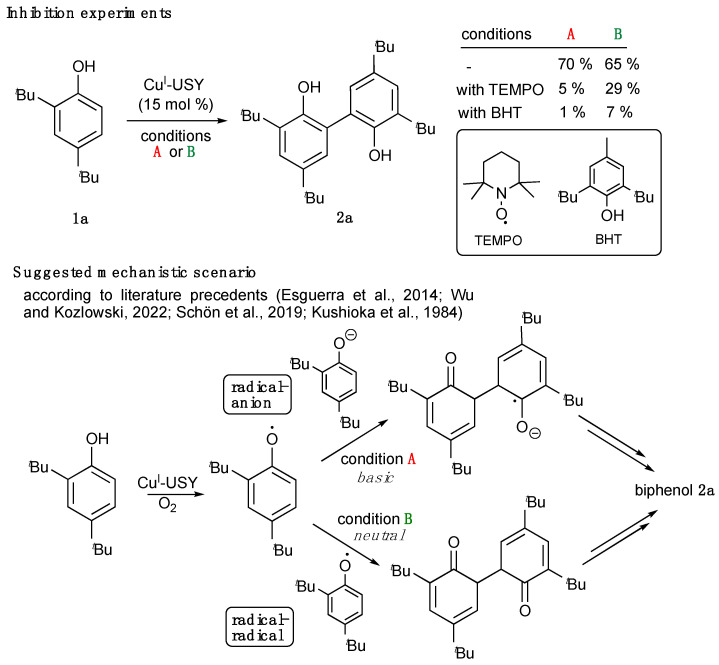
(**Top**) Inhibition experiments with TEMPO and BHT as radical scavengers (condition **A**: in MeOH in the presence of Cs_2_CO_3_ and condition **B**: in H_2_O). (**Bottom**) The suggested mechanistic scenario based on the many precedents in the literature (refs. [47,50,51,52] and references therein).

In order to check the involvement of a radical pathway in the case of our Cu^I^-catalyzed versions, inhibition experiments were conducted on the model reaction. Thus, the oxidative dimerization of 2,4-di-*tert*-butylphenol **1a** was performed in the presence of 2,2,6,6-tetramethylpiperidin-1-oxyl (TEMPO) and 2,6-di-*tert*-butyl-*p*-cresol (BHT) as radical scavengers. We observed that in methanol in the presence of cesium carbonate (condition **A**) or in water (condition **B**), the formation of the expected biphenol **2a** was clearly inhibited (Scheme 3, top), thus supporting the involvement of a radical pathway. However, no additional product between these scavengers and the possible radical forms of **1a** could be detected from these experiments.

According to these experimental results and the many precedents in the literature [47,50,51,52], we propose a radical–radical mechanistic scenario in the case of water as a solvent (i.e., under neutral conditions) and a radical–anion scenario in the case of methanol as a solvent (i.e., under basic conditions).

### 2.2. Biaryl Synthesis via Cu^I^-USY Homocoupling of Aryl Boronic Acids

#### 2.2.1. Setup of Coupling Conditions (Table 2)

In the case of the homocoupling of aryl boronic acids, we selected the 4-methoxyphenyl boronic acid **3a-*p*** (*p* for para) as a model substrate, due to its known reactivity in Chan–Evans–Lam (CEL) reactions.

The classical solvent for CEL reactions, dichloromethane, surprisingly did not allow full conversion and only led to trace amounts of the expected dimer **4a-*p***, whatever the temperature (entry 1). Toluene, which often proved to be a good solvent for zeolite-catalyzed organic reactions [34,35], gave similar results (entry 2). More polar solvents were then evaluated, but again conversion was moderate to low and **4a-*p*** could only be barely detected (entries 3–5).

**Table 2:**
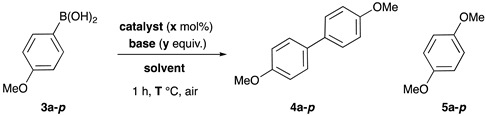
Screening of reaction conditions for the Cu^I^-USY-catalyzed homocoupling of 4-methoxyphenyl boronic acid **3a-*p*** *^a^*.

Entry	Catalyst (x mol%)	Solvent	T (°C)	Conversion *^b^* (%)	Yield *^c^* (4a + 5a %)
1	Cu^I^-USY (10)	CH_2_Cl_2_	rt to 40 *^d^*	45	traces
2	Cu^I^-USY (10)	PhMe	rt to 65 *^d^*	42	traces
3	Cu^I^-USY (10)	EtOAc	rt to 65 *^d^*	17	traces
4	Cu^I^-USY (10)	Me_2_CO	rt to 65 *^d^*	39	traces
5	Cu^I^-USY (10)	MeCN	rt to 65 *^d^*	40	traces
6	Cu^I^-USY (10)	^i^PrOH	65	18	traces
7	Cu^I^-USY (10)	EtOH	65	90	25
**8**	**Cu^I^-USY (10)**	**MeOH**	**65**	**100**	**56** + 16
9	Cu^I^-USY (10)	H_2_O	65	94	28
10	Cu^I^-USY (10)	CF_3_CH_2_OH	65	28	traces
11	Cu^I^-USY (10)	MeOH	25	100	38 + 10
12	Cu^I^-USY (10)	MeOH	45	100	45 + 14
13	Cu^I^-USY (20)	MeOH	65	100	54 + 11
14	Cu^I^-USY (5)	MeOH	65	100	47 + 16
15	Cu^I^-USY (1)	MeOH	65	100	46 + 19
16 *^e^*	Cu^I^-USY (10)	MeOH	65	66	13 + 1
17	Cu^II^-USY (10)	MeOH	65	89	46 + 9
18	H-USY (10)	MeOH	65	-	-
19	NH_4_-USY (10)	MeOH	65	43	traces
20	none	MeOH	65	42	traces
21	Cu^I^-β (10)	MeOH	65	100	51 + 19
22	Cu^I^-MOR (10)	MeOH	65	100	47 + 20
23	Cu^I^-ZSM5 (10)	MeOH	65	100	46 + 23

*^a^* Reactions run for 1 h under air with 4-methoxyphenyl boronic acid **3a** (0.25 M concentration) unless otherwise stated. *^b^* Conversion estimated by ^1^H NMR of the crude using dimethyl terephthalate as the internal standard. *^c^* Yield estimated by ^1^H NMR of the crude using dimethyl terephthalate as the internal standard. *^d^* Reactions performed at rt and monitored, then at 10 °C or higher, and checked again, etc., up to the mentioned temperature. *^e^* The reaction performed under argon. Bold line (8) highlighted the best results.

Despite the possibility of competitive CEL coupling, the reaction was also performed in protic solvents. Interestingly, such solvents provided low to full conversion within 1 h (entries 6–9), but among them, only methanol allowed us to obtain satisfactory yield of the expected dimer **4a-*p*** (entry 8). Nevertheless, and as suspected, the latter was accompanied by a non-negligible amount of the CEL-coupling product **5a-*p***. To look at the possible role of an H-bond, the strong H-bond donor and non-nucleophilic trifluoroethanol was examined. However, low conversion and yield were observed (entry 10). Decreasing reaction temperature lowered the formation of **5a-*p***, but also of the expected homocoupling product **4a-*p*** (entries 11–12 vs. 8). Increasing the catalyst amount did not change the results much (entry 13 vs. 8) but decreasing it did, as observed by the slight decrease in homocoupling product yields and the relative increase in coupling product yield (from 22 to 30%). Running the reaction under an inert atmosphere also induced lower conversion and yield (entry 16 vs. 8). This result prompted us to check the ability of Cu^II^-USY to catalyze this reaction. Under the optimal conditions, the latter was indeed active, but less than Cu^I^-USY, as conversion and yield were both lower (entry 17 vs. 8). Further control experiments showed that no reaction took place with native zeolite or without a catalyst (entries 18–20).

These results showed that Cu^I^-USY is an active catalyst for the dimerization of 4-methoxyphenyl boronic acid **4a** and that methanol is the best solvent, although it has been reported as deleterious in the classical CEL reaction [24], and that air is essential.

Due to the overall rod shape of the expected dimers, zeolites with linear internal pores would also be appropriate to host such kinds of compounds. To check this possibility, a series of three common zeolites with different pore size and shapes was applied as catalysts to the present homocoupling reaction (entries 21–23). Quite expectedly, Cu^I^-β, which exhibits two sets of channels with a rather large one (6.4 × 7.6 Å), gave results close to those achieved with Cu^I^-USY with its largest pores (7.4 × 12 Å; entry 21 vs. 8). Mordenite and ZSM-5, featuring smaller-sized channels (3–4 Å and 5–6 Å, respectively), provided similar results, but with a slightly lower amount of biaryl product and an increased amount of the CEL-coupling product (entries 22–23 vs. 21 vs. 8). In contrast to what was expected, smaller-sized-channel zeolites slightly favor the CEL-coupling reaction rather than the homocoupling reaction.

To go further, we also briefly explored the reactivity of boronic esters and related boron derivatives (Table 3). Indeed, it is well known that boronic acids are in equilibrium with their cyclic trimer boroxines, which can alter their reactivity in coupling reactions. Furthermore, boronic acids in methanol could also be in equilibrium with the corresponding methyl boronate. As the latter could be the actual reactive species, it was worth looking at their reactivity and comparing them.

As suspected, the methyl boronate reacted as rapidly (1 h) as its boronic acid parent and provided similar results (entry 2 vs. 1). The other screened boron derivatives reacted more slowly, with the formation of only small amounts of the expected product **4a-*p*** (entries 3–8 vs. 2 vs. 1). Pinacol ester was slowly transformed but mostly to the CEL-coupling product, i.e., **5a-*p***, although modest yields were observed (entries 3 and 4 vs. 1). In contrast, the MIDA analog was more rapidly converted but mostly to the corresponding boronic acid **3a-*p***. However, and quite surprisingly, no homocoupling nor CEL-coupling products were observed (entries 5, 6). This result suggests that the aminodiacid liberated upon MIDA hydrolysis may have poisoned the copper catalyst. Similarly, the trifluoroborate analog was also relatively rapidly converted, but again the hydrolysis products were the most preeminent products formed, together with some of the hydrolysis intermediates (entries 7, 8). Because no coupling products could be detected, it seems that the fluoride liberated upon hydrolysis ended up as copper fluoride, also poisoning the catalyst here.

These results seem to reflect the hydrolysis/solvolysis rate of the starting boronate ester or the tetrafluoroborate. They also confirmed that arylboronic acids are the real reagent in the present Cu^I^-USY-catalyzed homocoupling reaction.

#### 2.2.2. Substrate Scope and Limitations

**Scheme 4:**
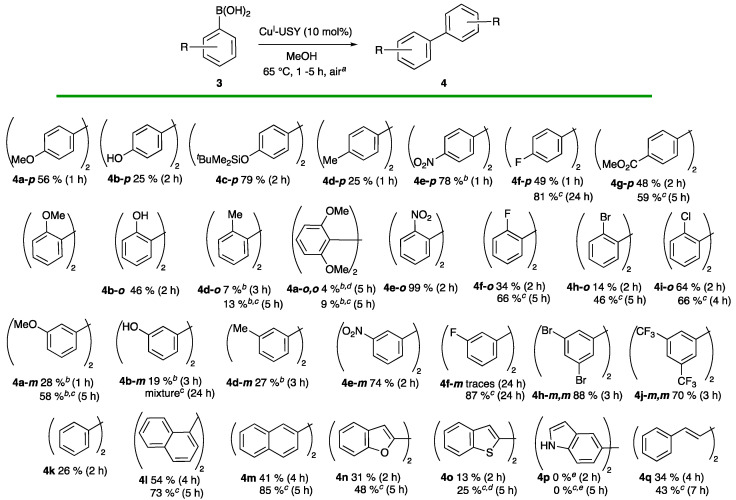
The scope of the Cu^I^-USY-catalyzed boronic acid homocoupling conditions. *^a^* Reactions run under optimized conditions at 65 °C under air during the indicated time unless otherwise stated. *^b^* Yield estimated by ^1^H NMR of the crude reaction mixture using dimethyl terephthalate as the internal standard. *^c^* The reaction was performed at room temperature during the indicated time. *^d^* The protodeboronated form was isolated in 42% yield. *^e^* Only the protodeboronated form, indole, was detected.

With conditions and the right type of substrate in hand, the scope and limitations of this Cu^I^-USY-catalyzed homocoupling of aryl boronic acids were investigated, mostly with benzene boronic acids substituted by different groups inducing various electronic or steric effects, in order to explore their possible roles in the reaction (Scheme 4).

When 4-methoxyphenyl boronic acid was allowed to react in the presence of Cu^I^-USY in refluxing MeOH, its consumption was completed after 1 h and the expected biphenyl **4a-*p*** was isolated with 56% yield, together with 1,4-dimethoxybenzene **5a-*p***, the CEL-coupling product, as shown previously (Table 2). Its hydroxy analog reacted similarly, but the corresponding biphenol **4b-*p*** was obtained with a lower yield, together with side-products, issued from phenolic oxidative degradation. As expected, the protection of this hydroxy group restored the homocoupling formation and even increased it, since higher yield of the silylated derivative **4c-*p*** was obtained and with only 4% of the CEL-coupling product **5c-*p***. The 4-tolyl boronic acid also gave the expected product **4d-*p*** together with various side-products, probably for the same reason, and only low yields could be achieved.

The *ortho*-analogs of these boronic acids provided the corresponding dimers **4a-*o*** and **4b-*o*** in similar but slightly higher yields (62–46 vs. 56–40%), except again for the 2-tolyl derivative **4d-*o***. Unfortunately, the sterically hindered tetramethoxylated biphenyl **4a-*o,o*** could only be formed in very low yield from 2,6-dimethoxyphenyl boronic acid, with 42% of the protodeboronation compound as a major product and 10% of the corresponding CEL-coupling product **5a-*o,o***.

Comparatively, phenyl boronic acids *para*- or *ortho*-substituted with electron-withdrawing groups (EWGs) led to the expected dimers **4e**-**4i** (-***p*** and/or -***o***) in yields higher, sometimes quantitatively, than those obtained with electron-donating groups (EDGs) (respectively, 46–99 vs. 13–79%). In some cases, performing the reaction at room temperature proved advantageous, since higher yields could be obtained (see, e.g., **4f-*p*** and **-*o***, 81 vs. 49% and 66 vs. 34%, and **4h-*o***, 46 vs. 14%, at, respectively, 25 or 65 °C). The latter results confirmed the sensitivity of such compounds.

In order to understand this discrepancy between *ortho*- and *para*-substituted phenyl boronic acids, the *meta*-analogs (in which only inductive effects would be operative) were engaged under the same conditions. In the EDG series, the 3-methoxy-, hydroxy-, or methylphenyl boronic acids provided the corresponding dimers **4a-*m***, **4b-*m***, and **4d-*m*** with yields like those observed for the *para*-derivatives **4a-*p***, **4b-*p***, and **4d-*p***. In the EWG series, results proved more scattered, with the *meta*-nitro derivative giving yield similar to the *para*-analog but lower to the *ortho*-analog (74 vs. 78 vs. 99%, respectively), while the *meta*-fluoro derivative only led to traces of the homocoupling product **4f-*m*** under standard conditions, but high yield if the reaction was run at room temperature, close to what was observed with the *para*-analog at the same temperature (87 vs. 81%, respectively). Interestingly, the 3,5-dibromo- and 3,5-ditrifluoromethyl-phenyl boronic acids led to the corresponding homocoupling products **4h-*m,m*** and **4j-*m,m*** in good to high yield (88 and 70%, respectively).

Submitted to the standard conditions, the simplest phenylboronic acid provided the expected biphenyl **4k** with a modest 26% isolated yield. The related but larger α- and β-naphthyl boronic acids also gave the corresponding dimers **4l** and **4m**, but higher yields could be achieved when the reactions were run at room temperature (73 vs. 54% and 85 vs. 41% at 25 vs. 65 °C).

Heteroaryl compounds were also briefly screened. 2,2′-bibenzofuran **4n** was isolated with 31% yield but with 48% yield when the reaction was performed at room temperature. While its sulfur congener **4o** could also be obtained but in lower yield (with 42% yield of the protodeboronated form—benzothiazole—as a major product), no traces of the expected bis-indole **4p** could be detected. In both cases, protodeboronation appeared as the preferred pathway. Interestingly, styryl boronic acid could also be converted to its dimer **4q** with modest yield.

As the latter provided an attractive access to substituted dienes, we attempted combining this easy-to-implement homocoupling with a Diels–Alder reaction (Table 4). For that, we submitted first the so-obtained (*E*,*E*)-1,4-diphenylbuta-1,3-diene **4q** to *N*-phenyl maleimide (NPM) and dimethyl acetylene dicarboxylate (DMAD) as standard, reactive dienophiles and we then tried to achieve a one-pot two-step reaction.

To look at its reactivity, the diene **4q** was first reacted with *N*-phenyl maleimide in refluxing toluene, a classical solvent for the Diels–Alder reaction. Under an inert atmosphere, the expected cycloadduct was readily obtained, while decomposition only occurred under air (entry 2 vs. 1). The presence or not of Cu^I^-USY did not alter the reaction efficiency, as almost the same yields of the cycloadduct were observed (entry 3 vs. 2). To achieve conditions closer to those allowing the Cu^I^-USY-catalyzed homocoupling (Table 2 and Scheme 4), the Diels–Alder reaction was performed in methanol. While no conversion was observed at room temperature, the cycloaddition process proved to be effective in methanol only at high temperature (entries 4–5) and yields could be improved by using an excess of *N*-phenyl maleimide (entries 6 to 8). Here, again, running the reaction under air proved deleterious (entry 9 vs. 7), while yields were slightly improved in the absence of Cu^I^-USY (entry 10 vs. 7). With acetylene dicarboxylate as dienophile (entries 11–14), the toluene conditions led to high yields of the expected adduct in the presence or not of a catalyst (entries 11–12). In this case, the presence of Cu^I^-USY proved deleterious when using methanol as a solvent (entry 14 vs. 13).

**Scheme 5:**
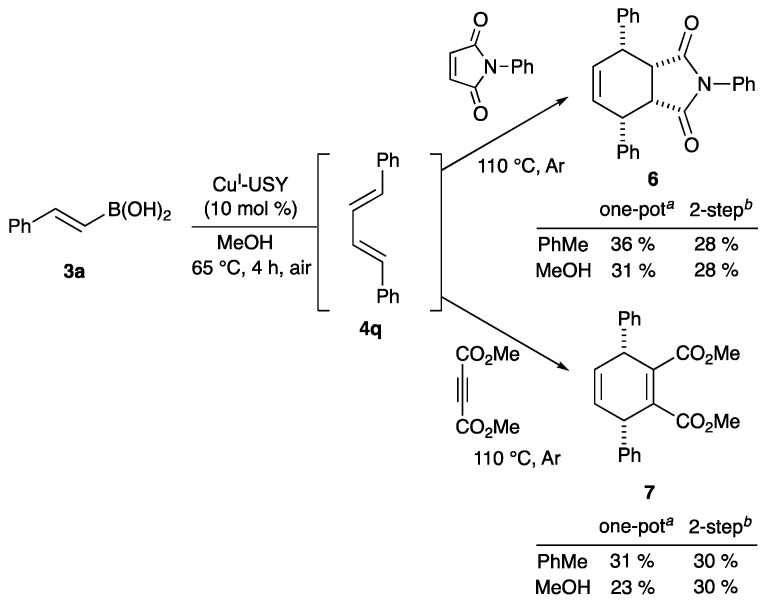
The evaluation of the potential of the one-pot Cu^I^-USY-catalyzed homocoupling/Diels–Alder process. *^a^* Isolated yield of the one-pot process (without the isolation of the intermediate **4q**). *^b^* Overall yield of the two-step sequence with the isolation/purification of **4q**.

We then evaluated the potential of the one-pot synthesis of cycloadducts **6** and **7** from styryl boronic acid by telescoping the Cu^I^-USY-catalyzed homocoupling reaction and the Diels–Alder reaction, without isolating the intermediate diene **4q** (Scheme 5). 

In both cases, the one-pot process was accomplished with the formation of the expected cycloadducts **6** and **7**. While the yields remained modest, it is worth noting that the efficiency of the one-pot process is comparable to that of the two-step sequence, without the need to isolate intermediate **4q**. This efficiency is even better with toluene as a solvent, especially with *N*-phenyl maleimide as diene.

#### 2.2.3. Cu^I^-USY Catalyst Recovery and Recycling 

**Figure 3:**
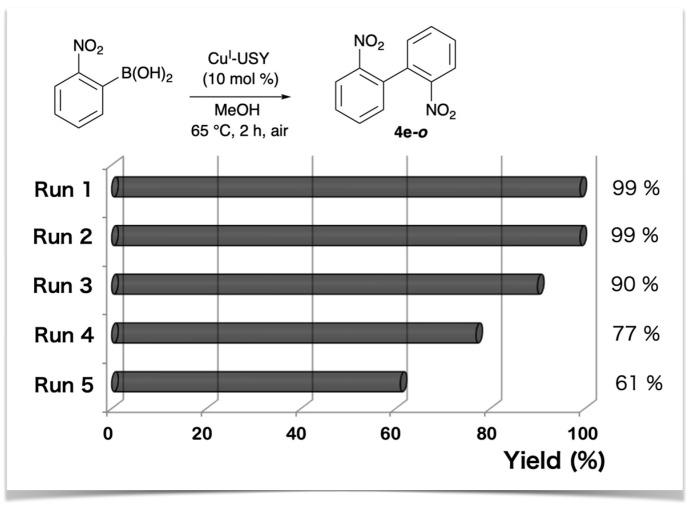
Recycling studies: homocoupling of 2-nitrophenyl boronic acid successively catalyzed by the same recovered Cu^I^-USY material.

The catalyst recycling and reuse were examined with one of the most reactive boronic acids, i.e., the 2-nitrophenyl boronic acid (see Scheme 4). Using the same recycling procedure as for the oxidative coupling (see Section 2.1.3), the catalyst could be used three times with similar efficiency, but after some decrease occurred, it could be used as often (Figure 3). Despite this decrease, noteworthy is that the recovered zeolitic materials keep a reasonable activity even after the fifth run.

#### 2.2.4. Mechanistic Scenario

After several and sometimes contradictory proposals for copper-catalyzed homocoupling of boronic acids, the actual mechanism was recently deciphered [53]. A nice combination of kinetic, NMR, cyclic voltammetry, conductimetry, and theoretical studies confirmed the role of a base and revealed the involvement of dimeric Cu^II^-Cu^II^ complexes, which evolve to a mixed-valence Cu^III^-Cu^I^ dimer allowing biaryl formation through Cu-Cu transmetalation and reductive elimination.

**Figure 4:**
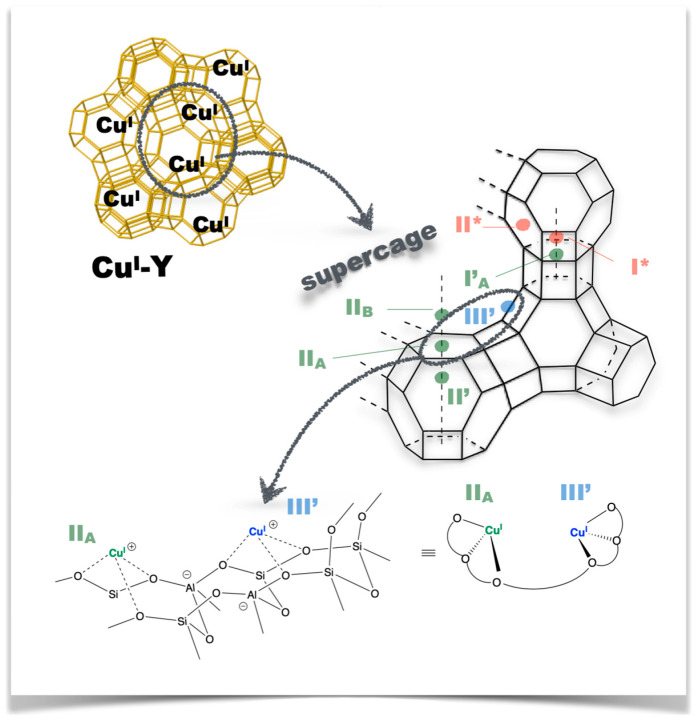
(**Top**) The location of copper ions in the Cu^I^-Y zeolite framework. (**Bottom**) A Zoomed-in view of the area including Cu^I^ cations located at site II and III’ positions to illustrate their proximity in space.

In order to link this relevant work to our Cu^I^-USY-catalyzed version, it is first worth noting that dimeric copper species and their presence within some zeolites have already been suggested as key active species for nitrogen oxide decomposition [54,55]. Therefore, dimeric copper complexes similar to those deduced from the above mechanistic study may occur within the so-called supercage in Y (faujasite)-type zeolites. Indeed, as Cu^I^ cations are located in the so-called site I′, II, and III′ positions within the faujasite structure (Figure 4, top left and middle right) [56], Cu^I^ cations located at site II and III′ positions at the supercage border could be close enough to independently react with boronic acids and then be bridged by the solvent (Figure 4, bottom).

**Scheme 6:**
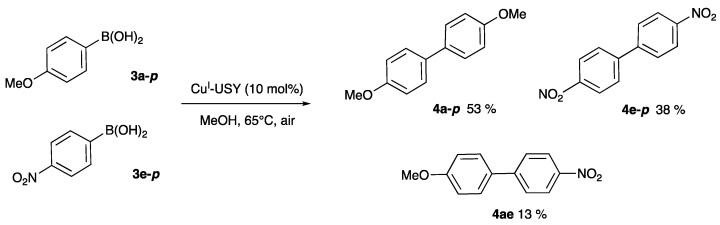
Competition experiment using 4-methoxyphenyl and 4-nitrophenyl boronic acids.

To test the viability of dimeric complexes as key intermediates in the present heterogeneous homocoupling, we performed a competition experiment (Scheme 6). Running a reaction under the setup conditions with two different boronic acids should lead to two homocoupling products and no cross-coupling product if each one is produced in a single zeolite cage through dimeric complexes. As expected, when submitting a 1:1 mixture of 4-methoxyphenyl and 4-nitrophenyl boronic acids **3a-*p*** and **3e-*p***, the corresponding homocoupling dimers **4a-*p*** and **4e-*p*** were produced in yield similar to the independent reaction for the 4-methoxy derivative and in lower yield for the 4-nitro derivative. Nevertheless, the cross-coupling product could also be detected, but in low yield (Scheme 6).

**Scheme 7:**
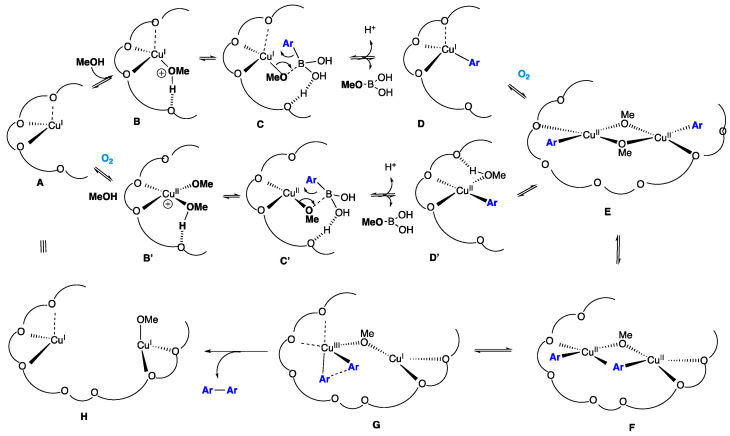
Mechanistic scenario for Cu^I^-USY-catalyzed homocoupling of aryl boronic acids.

As mentioned above, the presence of a base, especially hydroxide, is often mandatory for promoting a homocoupling reaction, and the recent mechanistic investigation showed its key role in the B-to-Cu transmetalation step. Since here no base is required, we wondered what could play this activating role. As shown above, solvents, especially hydroxylated solvents, proved to be essential (Table 2, entries 1–10). These results tend to support a mechanism in which methanol acts as a ligand completing the Cu coordination sphere with the zeolite framework, assists the transmetalation from boronic acid to copper (see **B**-**C**-**D** and/or **B**′-**C′**-**D′** in Scheme 7), and then helps in forming dimeric complexes (**E**). The latter would then allow Cu-Cu transmetalation (**F**) and reductive elimination (**G**) as reported, finally leading to the biaryl formation (see Scheme 7). In this cascade, the zeolite may stabilize copper intermediates, its oxygenated framework acting as a huge cooperative ligand. The latter could also assist in various proton transfers.

## 3. Materials and Methods

### 3.1. General Information

All starting materials were commercial and were used as received. Reactions were monitored by thin-layer chromatography carried out on silica plates (silica gel 60 F_254_, Merck, Darmstadt, Germany) using UV light for visualization. Column chromatographies were performed on silica gel 60 (0.040–0.063 mm, Merck) using mixtures of ethyl acetate (or diethyl ether) and cyclohexane as eluents. Evaporations of solvents were conducted under reduced pressure at temperatures less than 30 °C unless otherwise noted. Melting points (M.p.) were measured with a Stuart SMP30 apparatus (Cole-Parmer, Londres, UK) in open capillary tubes and are uncorrected. IR spectra were recorded neatly on a Bruker Alpha ATR Diamant (Bruker Optic, Ettingen, Germany). Values of wave numbers are reported in cm^−1^—^1^H and ^13^C NMR spectra were recorded on Bruker Avance spectrometers (Bruker Biospin, Ettingen, Germany) at 300, 400, or 500 MHz for ^1^H NMR experiments and 75, 100, and 126 MHz for ^13^C NMR experiments. Chemical shifts *δ* and coupling constants *J* are given in ppm and Hz, respectively. Chemical shifts *δ* are reported relative to the residual solvent as an internal standard (chloroform-d*_1_*: 7.26 ppm for ^1^H and 77.0 ppm for ^13^C). Carbon multiplicities were determined by DEPT 135 experiments. ElectroSpray Ionization (ESI), Desorption Chemical Ionization (DCI), and Atmospheric-Pressure Chemical Ionization (APCI) low-resolution mass spectra were obtained from the Service Commun de Spectroscopie de Masse of the Plateforme Technique, Institut de Chimie de Toulouse, as well as from the Service de Spectroscopie de Masse of the Fédération de Chimie Le Bel (FR2010).

### 3.2. General Experimental Procedures

Detailed experimental procedures, characterizations and spectral data for all compounds can be found in the Supplementary Materials.

#### 3.2.1. General Procedure for Preparation of Cu^I^-USY

Cu^I^-USY was prepared according to a well-established solid/solid exchange procedure [41]. Commercial NH_4_-USY was first loaded in an oven and heated at 550 °C for 4 h to give H-USY. A mixture of so-formed H-USY (1 g) and CuCl (376 mg, 1.0 equiv. related to the number of H-zeolite protons) were ground by a pestle and mortar and then heated in a furnace under flowing nitrogen at 350 °C. After 3 days of heating, the furnace was cooled down to room temperature and nitrogen feed was stopped; the resulting solid Cu-USY could be directly used or stored in a flask under argon for longer preservation.

#### 3.2.2. General Procedure for Cu^I^-USY-Catalyzed Oxidative-Phenol-Coupling Reactions

In methanol as solvent (condition **A**)—In a 5 mL round-bottomed flask, Cu^I^-USY (ca. 15 mg, 10 mol% of copper species), methanol as a solvent (0.5 mL), the phenol (0.5 mmol, 1.0 equiv.), and cesium carbonate as a base (0.125 mmol, 0.25 equiv.) were successively added. The flask was equipped with a reflux condenser and after 22 h stirring at 65 °C (unless otherwise stated), the reaction was complete as revealed by either a TLC or LCMS analysis. After cooling to room temperature, the resulting mixture was diluted with 1 M aqueous HCl solution (1 × 10 mL) and the solid materials were removed by filtration over a PTFE Millipore membrane (Darmstadt, Germany). The aqueous phase was then extracted with ethyl acetate (3 × 10 mL) and the resulting organic phases were combined, washed brine (1 × 10 mL), dried over Na_2_SO_4_, filtered, and evaporated. The resulting crude product was purified by column chromatography on a short pad of silica gel, eluting with pure cyclohexane, thus furnishing the expected biphenols in pure forms.

In water as solvent (condition **B**)—In a 5 mL round-bottomed flask, Cu^I^-USY (ca. 15 mg, 10 mol% of copper species), water as a solvent (0.5 mL), and the phenol (0.5 mmol) were successively added. The flask was equipped with a reflux condenser and after 22 h stirring at 100 °C (unless otherwise stated), the reaction was complete as revealed by either a TLC or LCMS analysis. After cooling to room temperature, the solid materials were removed by filtration over a PP Millipore membrane and further washed with ethyl acetate (1 × 50 mL). The organic phase was then recovered and dried over Na_2_SO_4_, filtered, and evaporated. The resulting crude product was purified by column chromatography on a short pad of silica gel, eluting with pure cyclohexane, thus furnishing the expected biphenols in pure forms.

#### 3.2.3. General Procedure for Cu^I^-USY-Catalyzed Homocoupling of Aryl Boronic Acids

In a 50 mL Schlenk flask, the aryl boronic acid (0.5 mmol, 1.0 equiv.), Cu^I^-USY (10 mg, 6 mol% of copper species), and MeOH (2 mL, c = 0.25 mol/L) were successively added. The reaction mixture was stirred at 65 °C under air and monitored by TLC until the full consumption of the starting material. Then, the reaction was cooled down to room temperature. After the addition of 15 mL of EtOAc, the mixture was stirred for an additional 2 h and then filtered over a filter funnel (50 mL, 4 (10–16 μm)) with celite and washed with ethyl acetate (4 × 5 mL). The filtrate was concentrated under reduced pressure and the crude was purified by flash chromatography over silica gel using a cyclohexane/EtOAc mixture. 

#### 3.2.4. General Procedure for Cu^I^-USY-Catalyzed Diels–Alder Reactions

In a screwcap reaction tube, 1,4-diphenyl-1,3-butadiene **4q** (0.5 mmol, 1 equiv.), dienophile *N*-phenyl maleimide, and Cu^I^-USY (6 mol%) were successively added in toluene (2 mL), and the tube was then sealed under argon. After 24 h heating at 110 °C, the Cu^I^-USY was removed by hot filtration with celite and washed with EtOAc. After cooling the filtered solution to room temperature, toluene was decanted and the solid was washed 4 times with cold diethyl ether (4 × 10 mL). The solid was dried to give the desired product 2,4,7-triphenyl-3a,4,7,7a-tetrahydro-1*H*-isoindole-1,3(2*H*)-dione (**6**) as a white solid. 

The procedure is similar in the case of acetylene dicarboxylate as the dienophile, except that after cooling down to room temperature, the solution was evaporated to obtain a yellow crude. The solid was then washed with 2-propanol and hexane to provide the title compound, namely dimethyl 3,6-diphenylcyclohexa-1,4-diene-1,2-dicarboxylate (**7**), as a colorless solid.

## 4. Conclusions

A new green heterogeneously catalyzed synthesis of biaryls has been reported here through oxidative homocoupling of phenols and homocoupling of (hetero)aryl- or vinylboronic acids. In both cases, the readily available copper(I)-exchanged zeolite Cu^I^-USY was the best catalyst for these reactions. 

Cu^I^-USY allowed the oxidative homocoupling of phenols under air either in warm methanol or water with good to high yields. Under the latter conditions, the catalyst could be easily recovered and reused at least three times.

Alternatively, a new ligand- and base-free Cu^I^-USY-catalyzed protocol was described for the homocoupling of (hetero)aryl- or vinylboronic acids. These heterogeneous conditions offered a mild and efficient access to a variety of biaryls under simple and practical conditions, i.e., refluxing in methanol, under air, and without any additive. The catalyst could be easily recovered and recycled three to five times. Applied to vinyl boronic acids, this mild homocoupling reaction readily produced dienes, which could directly be engaged in a Diels–Alder reaction. The two steps were also coupled together in a one-pot process.

## Figures and Tables

**Table 3:**
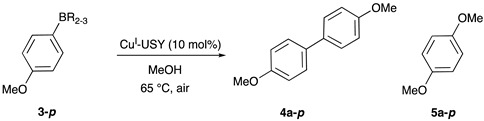
Screening of various boron derivatives for the Cu^I^-USY-catalyzed formation of 4,4′-dimethoxy-1,1′-biphenyl **4a-*p*** *^a^*.

Entry	Boron Derivative	Time (h)	Conversion *^b^* (%)	Yield *^c^* (4a + 5a %)
1	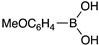	1	100	56 + 16
2	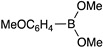	1	100	48 + 5
3	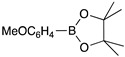	1	26	4 + 13
4		5	55	10 + 32
5	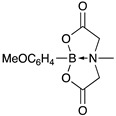	1	78	0 + 0 + 2 *^d^*
6		5	79	0 + 0 + 2 *^d^*
7	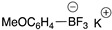	1	61	0 + 4 + 7 *^d^* + 3 *^e^*
8		5	61	7 + 5 + 8 *^e^*

*^a^* Reactions run at 65 °C under air with the boron derivative (0.25 M concentration) unless otherwise stated. *^b^* Conversion estimated by ^1^H NMR of the crude reaction mixture using dimethyl terephthalate as the internal standard. *^c^* Yield estimated by ^1^H NMR of the crude using dimethyl terephthalate as the internal standard. *^d^* Yield of 4-methoxyphenyl boronic **5a** acid also formed. *^e^* Yield of 4-methoxyphenyl difluoroboronic acid also formed.

**Table 4:**
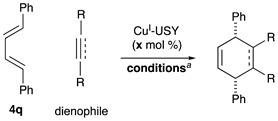
Screening of reaction conditions for the cycloaddition of (*E*,*E*)-1,4-diphenylbuta-1,3-diene **4q** with *N*-phenylmaleimide (NPM) or dimethyl acetylenedicarboxylate (DMAD) as dienophiles *^a^*.

Entry	Dienophile	Ratio 4q/Dienophile	Cu^I^-USY (x mol%)	Conditions *^a^*	Yield *^b^* (%)
1	NPM	1.2:1	0	PhMe, 110 °C, air	0
2	NPM	1.2:1	0	PhMe, 110 °C, Ar	84
3	NPM	1.2:1	6	PhMe, 110 °C, Ar	80
4	NPM	1.2:1	6	MeOH, 90 °C, Ar	18
5	NPM	1.2:1	6	MeOH, 110 °C, Ar	50
6	NPM	1:1	6	MeOH, 110 °C, Ar	65
7	NPM	1:2	6	MeOH, 110 °C, Ar	62
8	NPM	1:4	6	MeOH, 110 °C, Ar	84
9	NPM	1:2	6	MeOH, 110 °C, air	0
10	NPM	1:2	0	MeOH, 110 °C, Ar	84
11	DMAD	1:2	0	PhMe, 110 °C, Ar	89
12	DMAD	1:2	6	PhMe, 110 °C, Ar	85
13	DMAD	1:2	0	MeOH, 110 °C, Ar	88
14	DMAD	1:2	6	MeOH, 110 °C, Ar	23

*^a^* Reactions run with (*E*,*E*)-1,4-diphenylbuta-1,3-diene **4q** (0.5 mmol) in a sealed tube. *^b^* Isolated yield of the expected cycloadduct.

## Data Availability

Data are contained within the article and Supplementary Materials.

## Supplementary Materials

The following supporting information can be downloaded at: https://www.mdpi.com/article/10.3390/molecules29235552/s1, Detailed experimental procedures and spectral data for all compounds. References [57,58,59,60,61,62,63,64,65,66,67] are cited in the Supplementary Materials.
