# Supplementary material for: CuI-Zeolite Catalysis for Biaryl Synthesis via Homocoupling Reactions of Phenols or Aryl Boronic Acids
Source: Molecules. 2024 Nov 25;29(23):5552. doi: 10.3390/molecules29235552 (PMC11643476; doi:10.3390/molecules29235552)

SUPPORTING INFORMATION

Cu^{I}-Zeolite Catalysis for Biaryl Synthesis *via* Homocoupling of Phenols and Aryl Boronic Acids

Xiaohui Di[1], Tony Garnier[1], Arnaud Clerc[1], Eliott Jung[1], Christian Lherbet[2], Valérie Bénéteau[1], Patrick Pale[1,*] and Stefan Chassaing[1,*]

[1] *Catalyse Organométallique, Synthèse organique et Santé (COSyS), Institut de Chimie (UMR-CNRS 7177), Université de Strasbourg; Strasbourg, France; chassaing@unistra.fr*

[2] *LSPCMIB, Université Toulouse 3 Paul Sabatier, UMR CNRS UPS 5068, 118 route de Narbonne, 31062 Toulouse cedex 9, France*

chassaing@unistra.fr; ppale@unistra.fr

CONTENTS

SPECTROSCOPIC DATA S3-S12

REFERENCES S12-S13

COMPILATION OF NMR SPECTRA ^{1}H, ^{13}C & DEPT135 S14-S101

SPECTROSCOPIC DATA

All products reported in this work are known compounds. Spectroscopic data (as well as NMR spectra) are given for a large selection of biaryl products as representative examples.

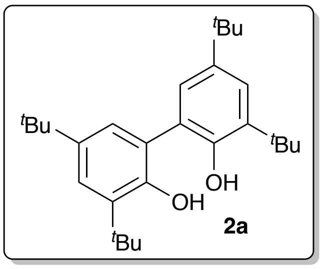

4,4',6,6'-Tetra-*tert*-butyl-2,2'-biphenol (2a) - CAS 457625-29-5: From 2,4-di-*tert*-butylphenol, the expected 2,2'-biphenol **2a** was isolated as a white solid. – Yields 70% (conditions **A** in MeOH) and 65% (conditions **B** in H_2O). – R_f = 0.40 (cyclohexane). – Mp 195-198 °C. – FTIR-ATR (neat) 3520, 2960, 1440, 1360 cm^{-1}. – ^{1}H NMR (300 MHz, $CDCl_3$): δ = 7.43 (d, ^4J = 2.4 Hz, 2H), 7.15 (d, ^4J = 2.4 Hz, 2H), 5.25 (s, 2H, OH)1.49 (s, 18H), 1.36 (s, 18H). – ^{13}C NMR (75 MHz, $CDCl_3$): δ = 149.8, 143.0, 136.2, 125.3, 124.8, 122.3, 35.2, 34.5, 31.7, 29.7. – MS (ESI, negative mode) *m/z* (rel intensity) 409 (*[M-H]*$^-$, 100). *For previous characterization of* ***2a**, see Ref. 1.*

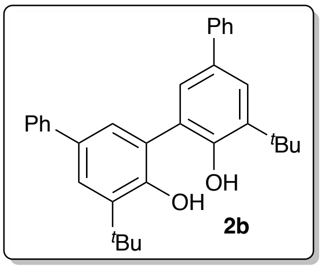

6,6'-Di-*tert*-butyl-4,4'-diphenyl-2,2'-biphenol (2b) - CAS 128378-50-7: From 2-*tert*-butyl-4-phenylphenol, the expected 2,2'-biphenol **2b** was isolated as a light brown solid. – Yields 55% (conditions **A** in MeOH) and 80% (conditions **B** in H_2O). – R_f = 0.45 (98:2 cyclohexane/EtOAc). – Mp 81-86 °C. – FTIR-ATR (neat) 3520, 2960, 1430, 1240 cm^{-1}. – ^{1}H NMR (300 MHz, $CDCl_3$): δ = 7.64 (d, ^4J = 2.4 Hz, 2H), 7.61-7.57 (m, 4H), 7.46-7.41 (m, 6H), 7.36-7.30 (m, 2H), 5.43 (s, 2H, OH), 1.52 (s, 18H). – ^{13}C NMR (75 MHz, $CDCl_3$): δ = 151.8, 140.9, 137.7, 133.8, 126.8, 127.2, 126.9, 126.8, 122.9, 35.2, 29.6. – MS (ESI, negative mode) *m/z* (rel intensity) 449 (*[M-H]*$^-$, 100). *For previous characterization of* ***2b**, see Ref. 1.*

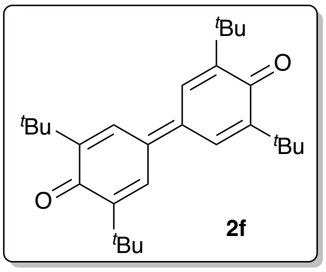

3,3',5,5'-Tetra-*tert*-butyl-4,4'-biphenoquinone (2f) - CAS 2455-14-3: From 2,6-di-*tert*-butylphenol, the expected 4,4'-biphenoquinone **2f** was isolated as a brown solid. – Yields 21% (conditions **A** in MeOH) and 40% (conditions **B** in H_2O). – R_f = 0.30 (cyclohexane). – Mp 243-244 °C. – FTIR-ATR (neat) 2960, 1600, 1360 cm^{-1}. – ^{1}H NMR (300 MHz, $CDCl_3$): δ = 7.71 (s, 4H), 1.36 (s, 36H). – ^{13}C NMR (75 MHz, $CDCl_3$): δ = 186.4, 150.4, 136.1, 126.0, 36.0, 29.6. – MS (DCI, positive mode) *m/z* (rel

intensity) 409 (*[MH]*$^+$, 50), 411 (*[M+3H]*$^+$, 70), 428 (*[M+2H+NH$_4$]*$^+$, 100). *For previous characterization of **2f**, see Ref. 2.*

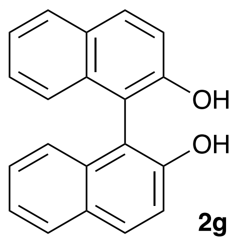

2g

1,1'-Bi-2-naphthol (2g) - CAS 602-09-5: From 2-naphthol, the expected binaphthol **2g** was isolated as a white solid. – Yield 60% (conditions **B** in H_2O). – R_f = 0.60 (7:3 cyclohexane/EtOAc). – Mp 206-212 °C. – FTIR-ATR (neat) 3480, 3400, 1600, 1380, 1220, 1180, 1150 cm^{-1}. – ^{1}H NMR (300 MHz, $CDCl_3$): δ = 7.98 (d, ^3J = 8.9 Hz, 2H), 7.90 (dd, ^3J = 8.9 Hz, ^4J = 1.2 Hz, 2H), 7.40-7.29 (m, 6H), 7.16 (td, ^3J = 8.1 Hz, ^5J = 0.6 Hz, 2H), 5.05 (s, 2H, O<u>H</u>). – ^{13}C NMR (75 MHz, $CDCl_3$): δ = 152.7, 133.4, 131.4, 129.4, 128.4, 127.5, 124.2, 124.0, 117.7, 110.8. – MS (ESI, negative mode) *m/z* (rel intensity) 285 (*[M-H]*$^-$, 100). *For previous characterization of **2g**, see Ref. 3.*

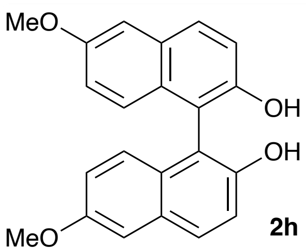

2h

6,6'-Dimethoxy-1,1'-bi-2-naphthol (2h) - CAS 145372-06-1: From 6-methoxy-2-naphthol, the expected binaphthol **2h** was isolated as a dark brown solid. – Yield 65% (conditions **B** in H_2O). – R_f = 0.40 (7:3 cyclohexane/EtOAc). – Mp 193-197 °C. – FTIR-ATR (neat) 3550, 3400, 1600, 1370, 1370, 1240, 1160 cm^{-1}. – ^{1}H NMR (300 MHz, $CDCl_3$): δ = 7.83 (d, ^3J = 9.0 Hz, 2H), 7.33 (d, ^3J = 9.0 Hz, 2H), 7.20 (d, ^4J = 2.5 Hz, 2H), 7.06 (d, ^3J = 9.0 Hz, 2H), 6.98 (dd, ^3J = 9.0 Hz, ^4J = 2.5 Hz, 2H), 4.96 (s, 2H, O<u>H</u>), 3.89 (s, 3H). – ^{13}C NMR (75 MHz, $CDCl_3$): δ = 156.3, 150.9, 130.3, 129.9, 128.5, 125.8, 119.8, 118.1, 111.3, 106.8, 55.3. – MS (ESI, negative mode) *m/z* (rel intensity) 345 (*[M-H]*$^-$, 100). *For previous characterization of **2h**, see Ref. 3.*

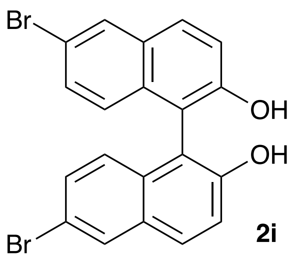

2i

6,6'-Dibromo-1,1'-bi-2-naphthol (2i) - CAS 13185-00-7: From 6-bromo-2-naphthol, the expected binaphthol **2i** was isolated as a dark brown solid. – Yield 43% (conditions **B** in H_2O). – R_f = 0.40 (7:3 cyclohexane/EtOAc). – Mp 169-179 °C. – FTIR-ATR (neat) 3450, 1590, 1160, 1150 cm^{-1}. – ^{1}H NMR (300 MHz, $CDCl_3$): δ = 8.04 (d, ^4J = 2.0 Hz, 2H), 7.87 (d, ^3J = 8.9 Hz, 2H), 7.38 (d, ^3J = 9.0 Hz, 2H), 7.36 (dd, ^3J = 8.9 Hz, ^4J = 2.0 Hz, 2H), 6.95 (d, ^3J = 9.0 Hz, 2H), 5.05 (s, 2H, O<u>H</u>). – ^{13}C NMR (75 MHz, $CDCl_3$): δ = 152.9, 131.9, 130.8, 130.6, 130.5, 130.4, 125.8, 118.9, 118.0, 110.6. – MS (ESI, negative mode) *m/z* (rel intensity) 441 (*[M(^{79}Br,^{79}Br)-H]*$^-$, 50), 443 (*[M(^{79}Br,^{81}Br)-H]*$^-$, 100), 445 (*[M(^{81}Br,^{81}Br)-H]*$^-$, 50). *For previous characterization of **2i**, see Ref. 3.*

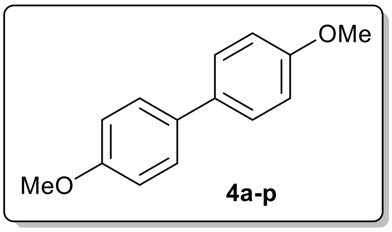

4,4'-Dimethoxy-1,1'-biphenyl (4a-p) - CAS 2132-80-1: From 4-methoxyphenylboronic acid, the expected biaryl **4a-p** was isolated as a white solid. – Yield 53%. – R_f = 0.65 (70:30 cyclohexane/EtOAc). – FTIR-ATR (neat) 2957, 2914, 2839, 1603, 1568, 1497, 1464, 1435, 1328, 1273, 1240, 1182, 1137, 1039, 1011, 996 cm^{-1}. – ^{1}H NMR (300 MHz, $CDCl_3$): δ = 7.53-7.45 (m, 4H), 7.00-6.92 (m, 4H), 3.85 (s, 6H). – ^{13}C NMR (126 MHz, $CDCl_3$): δ = 158.9 (Cq), 133.7, 127.9, 114.3 (Cq), 55.6 (CH_3). – MS (EI, positive mode) *m/z* (rel intensity) 214 (*[M]*$^+$, 100), 190 (100), 171 (43), 156 (11), 128 (18). *For previous characterization of* ***4a-p****, see Ref. 4.*

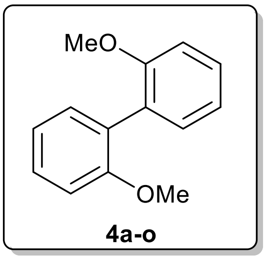

2,2'-Dimethoxy-1,1'-biphenyl (4a-o) - CAS 4877-93-4: From 2-methoxyphenylboronic acid, the expected biaryl **4a-o** was isolated as a white solid. – Yield 62%. – R_f = 0.67 (70:30 cyclohexane/EtOAc). – FTIR-ATR (neat) 3057, 3024, 2962, 2929, 2835, 1590, 1500, 1481, 1455, 1427, 1298, 1283, 1253, 1226, 1164, 1126, 1111, 1054, 1020, 1000 cm^{-1}. – ^{1}H NMR (300 MHz, $CDCl_3$): δ = 7.37-7.31 (m, 2H), 7.28-7.25 (m, 2H), 7.05-6.97 (m, 4H). 3.79 (s, 6H). – ^{13}C NMR (126 MHz, $CDCl_3$): δ = 157.0 (Cq), 131.5, 128.6, 127.8, 120.3, 111.1 (Cq), 55.7 (CH_3). – MS (EI, positive mode) *m/z* (rel intensity) 214 (*[M]*$^+$, 100), 199 (18), 184 (38). *For previous characterization of* ***4a-o****, see Ref. 4.*

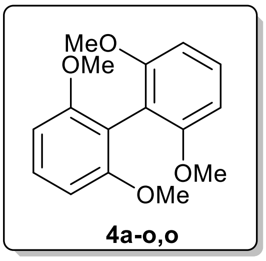

2,2',6,6'-Tetramethoxy-1,1'-biphenyl (4a-o,o) - CAS 19491-10-2: The biaryl **4a-o,o** was obtained from 2,6-dimethoxyphenyl)boronic acid. – Yields 4% (conditions at 65 C°) and 9% (conditions at 25 C°). – R_f = 0.76 (70:30 cyclohexane/EtOAc). – ^{1}H NMR (300 MHz, $CDCl_3$): δ = 7.29 (t, *J* = 8.1 Hz, 2H), 6.66 (d, *J*= 8.1 Hz, 4H), 3.72 (s, 12H). *For previous characterization of* ***4a-o,o****, see Ref. 4.*

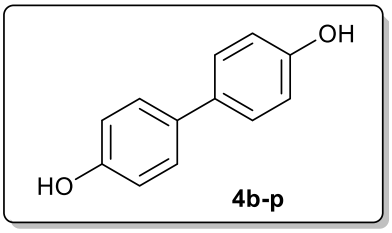

4,4'-Dihydroxy-1,1'-biphenyl (4b-p) - CAS 92-88-6: From 4-hydroxyphenylboronic acid, the expected 1,1'-biphenyl **4b-p** was isolated as an orange solid. – Yield 25%. – R_f = 0.50 (70:30 cyclohexane/EtOAc). – FTIR-ATR (neat) 3344, 3020, 2954, 2921, 2852, 1887, 1705, 1608, 1589, 1494, 1420, 1377, 1294, 1234, 1173, 1134, 1116, 1020, 1001 cm^{-1}. – ^{1}H NMR (500 MHz, DMSO-d_6): δ = 9.40 (s, 2H), 7.37-7.35 (m, 4H), 6.80-6.77 (m, 4H). – ^{13}C NMR (126 MHz, DMSO-d_6): δ = 156.2 (Cq), 131.2, 127.0, 115.6. – MS (ESI, positive mode) *m/z* (rel intensity) 186 (*[M]*$^+$, 100). *For previous characterization of* ***4b-p****, see Ref. 6.*

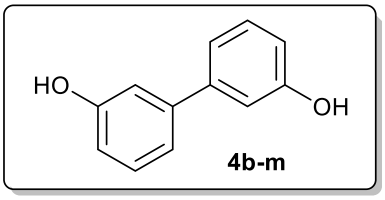

3,3'-Dihydroxy-1,1'-biphenyl (4b-m) - CAS 612-76-0: The biaryl **4b-m** was obtained from 3-hydroxyphenylboronic acid. – Yield 19%. – ^{1}H NMR (300 MHz, $CDCl_3$): δ = 7.30-7.27 (m, 2H), 7.14-7.12 (m, 2H), 7.04-7.03 (m, 2H). 6.84-6.82 (m, 2H). *For previous characterization of **4b-m**, see Ref. 6.*

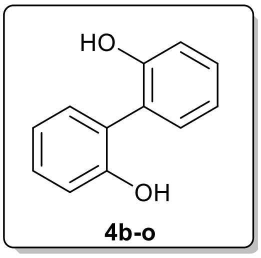

2,2'-Dihydroxy-1,1'-biphenyl (4b-o) - CAS 1806-29-7: From 3-hydroxyphenylboronic acid, the expected biaryl **4b-o** was isolated as a yellow solid. – Yield 46%. – R_f = 0.34 (70:30 cyclohexane/EtOAc). – FTIR-ATR (neat) 3268, 3171, 2919, 2850, 1705, 1605, 1588, 1572, 1480, 1437, 1370, 1340, 1267, 1211, 1111, 1093, 1045, 1005, 937 cm^{-1}. – ^{1}H NMR (500 MHz, $CDCl_3$): δ = 7.56-7.53 (m, 2H), 7.50-7.47 (m, 2H), 7.29-7.24 (m, 4H), 5.77 (s, 2H). – ^{13}C NMR (126 MHz, $CDCl_3$): δ = 153.0 (Cq), 131.4, 130.2, 123.6, 121.8, 116.8. – MS (ESI, positive mode) *m/z* (rel intensity) 186 (*[M]*$^{+}$, 100). *For previous characterization of **4b-o**, see Ref. 7.*

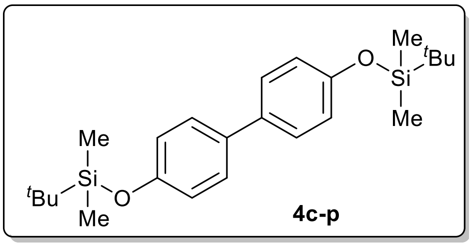

4,4'-bis((*tert*-Butyldimethylsilyl)oxy)-1,1'-biphenyl (4c-p) - CAS 1492015-05-0: From (4-((*tert*-butyldimethylsilyl)oxy)phenyl)boronic acid, the expected biaryl **4c-p** was obtained. – Yield 79%. – R_f = 0.75 (95:5 cyclohexane/EtOAc). – ^{1}H NMR (500 MHz, $CDCl_3$): δ = 7.42-7.39 (m, 4H), 6.89-6.86 (m, 4H), 1.00 (s, 18H), 0.22 (s, 12 H). – ^{13}C NMR (126 MHz, $CDCl_3$): δ = 154.9 (Cq), 134.2, 127.8, 120.4, 25.9, 18.4, -4.2. *For previous characterization of **4c-p**, see Ref. 8.*

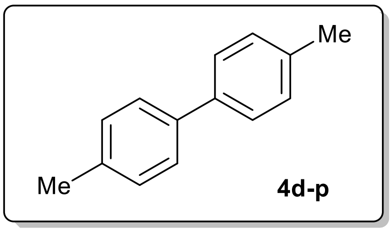

4,4'-Dimethyl-1,1'-biphenyl (4d-p) - CAS 613-33-2: The biaryl **4d-p** was obtained from 4-tolylboronic acid. – Yield 25%. – FTIR-ATR (neat) 3021, 2913, 2852, 1902, 1500, 1487, 1445, 1311, 1178, 1112, 1037, 1005, 837, 816, 724, 547, 501, 427 cm^{-1}. – ^{1}H NMR (300 MHz, $CDCl_3$): δ = 7.50-7.46 (m, 4H), 7.26-7.21 (m,4H), 2.39 (s, 6H). – ^{13}C NMR (126 MHz, $CDCl_3$): δ = 138.4 (Cq), 136.8, 129.6, 126.9, 21.2. – MS (ESI, positive mode) *m/z* (rel intensity) 182 (*[M]*$^{+}$, 100). *For previous characterization of **4d-p**, see Ref. 4.*

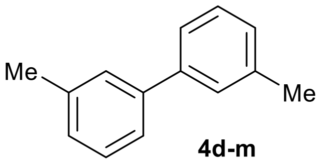

4d-m

3,3'-Dimethyl-1,1'-biphenyl (4d-m) - CAS 612-75-9: The biaryl **4d-m** was obtained from 3-tolylboronic acid. – Yield 27%. – R_f = 0.56 (cyclohexane). – ^{1}H NMR (500 MHz, $CDCl_3$): δ = 7.41-7.37 (m, 4H), 7.35-7.29 (m, 2H), 7.18-7.14 (m, 2H), 2.42 (s, 6H). *For previous characterization of **4d-m**, see Ref. 4.*

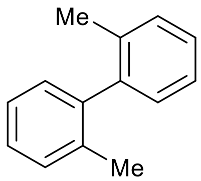

4d-o

2,2'-Dimethyl-1,1'-biphenyl (4d-o) - CAS 605-39-0: The biaryl **4d-o** was obtained from 2-tolylboronic acid. – Yield 7% (conditions at 65 C°) and 13% (conditions at 25 C°). – ^{1}H NMR (300 MHz, $CDCl_3$): δ = 7.43-7.38 (m, 4H), 7.36-7.31 (m, 2H), 7.19-7.14 (m, 2H), 2.43 (s, 6H). *For previous characterization of **4d-p**, see Ref. 4.*

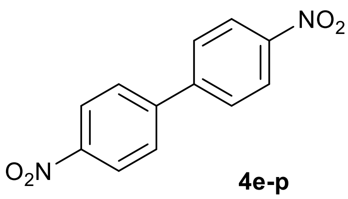

4e-p

4,4'-Dinitro-1,1'-biphenyl (4e-p) - CAS 1528-74-1: From 4-nitrophenylboronic acid, the expected biaryl **4e-p** was isolated as a yellow solid. – Yield 78%. – R_f = 0.66 (70:30 cyclohexane/EtOAc). – FTIR-ATR (neat) 3096, 2921, 2857, 2444, 1928, 1794, 1597, 1508, 1476, 1393, 1375, 1338, 1260, 1180, 1106, 1006 cm^{-1}. – ^{1}H NMR (500 MHz, $CDCl_3$): δ = 8.38-8.33 (m, 4H), 7.81-7.77 (m, 4H). – ^{13}C NMR (126 MHz, $CDCl_3$): δ = 148.1, 145.0, 128.4, 124.4. – MS (ESI, positive mode) *m/z* (rel intensity) 244 (*[M]*$^+$, 100). 214 (34), 151 (44). *For previous characterization of **4e-p**, see Ref. 5.*

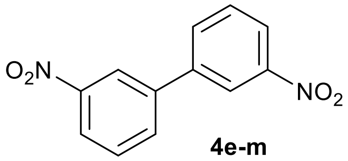

4e-m

3,3'-Dinitro-1,1'-biphenyl (4e-m) - CAS 958-96-3: From 3-nitrophenylboronic acid, the expected biaryl **4e-m** was isolated as a yellow solid. – Yield 74%. – R_f = 0.64 (70:30 cyclohexane/EtOAc). – FTIR-ATR (neat) 3384, 3092, 3080, 2922, 2852, 1706, 1622, 1593, 1519, 1464, 1417, 1344, 1300, 1266, 1214, 1103, 1082, 998 cm^{-1}. – ^{1}H NMR (300 MHz, $CDCl_3$): δ = 8.52-8.49 (m, 2H), 8.33-8.29 (m, 2H), 7.99-7.96 (m, 2H), 7.73-7.68 (m, 2H). – ^{13}C NMR (126 MHz, $CDCl_3$): δ = 148.7 (Cq), 140.2, 133.1, 130.2, 123.1, 122.0. – MS (ESI, positive mode) *m/z* (rel intensity) 244 (*[M]*$^+$, 100). *For previous characterization of **4e-m**, see Ref. 4.*

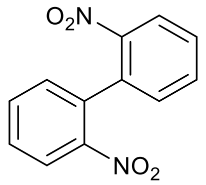

4e-o

2,2'-Dinitro-1,1'-biphenyl (4e-o) - CAS 2436-96-6: From 2-nitrophenylboronic acid, the expected biaryl **4e-o** was isolated as an orange solid. – Yield 99%. – R_f = 0.41 (70:30 cyclohexane/EtOAc). – FTIR-ATR (neat) 1605, 1571, 1514, 1467, 1349, 1297, 1163, 1145, 1103, 1006, 955 cm^{-1}. – ^1H NMR (300 MHz, $CDCl_3$): δ = 8.23-8.21 (m, 2H), 7.69 (td, ^3J = 7.5 Hz, ^4J = 1.4 Hz, 2H), 7.62-7.58 (m, 2H), 7.31-7.29 (m, 2H). – ^{13}C NMR (126 MHz, $CDCl_3$): δ = 147.2 (Cq), 134.2 (Cq), 133.5, 130.9, 129.2, 124.9. – MS (EI, negative mode) *m/z* (rel intensity) 198 (*[M-NO$_2$]$^+$*, 100), 168 (44), 139 (42), 115 (30). *For previous characterization of **4e-o**, see Ref. 9.*

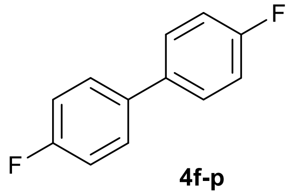

4f-p

4,4'-Difluoro-1,1'-biphenyl (4f-p) - CAS 398-23-2: From 4-fluorophenylboronic acid, the expected biaryl **4f-p** was isolated as a pale yellow solid. – Yields 49% (conditions at 65 C°) and 81% (conditions at 25 C°). – R_f = 0.81 (70:30 cyclohexane/EtOAc). – FTIR-ATR (neat) 3068, 3048, 2921, 2853, 1891, 1594, 1487, 1394, 1320, 1226, 1156, 1108, 1004, 803 cm^{-1}. – ^1H NMR (500 MHz, $CDCl_3$): δ = 7.52-7.45 (m, 4H), 7.15-7.08 (m, 4H). – ^{13}C NMR (126 MHz, $CDCl_3$): δ = 162.4 (d, J = 245.0 Hz, Cq), 136.4 (d, J = 3.0 Hz), 128.6 (d, J = 8.0 Hz), 115.7 (d, J = 21.0 Hz). – ^{19}F NMR (500 MHz, $CDCl_3$): δ = -115.7. – MS (EI, positive mode) *m/z* (rel intensity) 190 (*[M]$^+$*, 100). *For previous characterization of **4f-p**, see Ref. 5.*

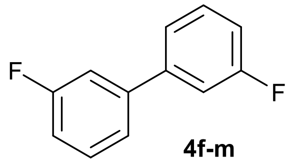

4f-m

3,3'-Difluoro-1,1'-biphenyl (4f-m) - CAS 396-64-5: From 3-fluorophenylboronic acid, the expected biaryl **4f-m** was isolated as a colorless solid. – Yield 87% (conditions at 25 C°). – R_f = 0.77 (70:30 cyclohexane/EtOAc). – FTIR-ATR (neat) 3208, 2955, 2920, 2851, 2261, 2081, 1611, 1580, 1453, 1420, 1410, 1377, 1258, 1193, 1157, 1028, 935 cm^{-1}. – ^1H NMR (500 MHz, $CDCl_3$): δ = 7.44-7.39 (m, 2H), 7.37-7.34 (m, 2H), 7.29-7.26 (m, 2H), 7.09-7.05 (m, 2H). – ^{13}C NMR (126 MHz, $CDCl_3$): δ = 163.3 (d, J = 246.1 Hz, Cq), 142.3 (dd, J = 7.7 Hz, J = 2.3 Hz, Cq), 130.5 (d, J = 8.4 Hz), 122.9 (d, J = 2.9 Hz), 114.8 (d, J = 21.2 Hz), 114.2 (d, J = 22.3 Hz). – ^{19}F NMR (500 MHz, $CDCl_3$): δ = -112.8. – MS (EI, positive mode) *m/z* (rel intensity) 190 (*[M]$^+$*, 100). *For previous characterization of **4f-m**, see Ref. 10.*

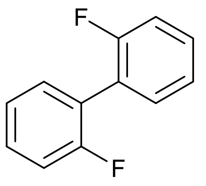

4f-o

2,2'-Difluoro-1,1'-biphenyl (4f-o) - CAS 388-82-9: From 2-fluorophenylboronic acid, the expected biaryl **4f-o** was isolated as a white solid. – Yields 34% (conditions at 65 C°) and 66% (conditions at 25 C°). – R_f = 0.77 (70:30 cyclohexane/EtOAc). – FTIR-ATR (neat) 3201, 2922, 2852, 2261, 1432, 1411, 1192 cm^{-1}. – ^{1}H NMR (300 MHz, CDCl$_3$): δ = 7.41-7.35 (m, 4H), 7.24-7.21 (m, 2H), 7.19-7.15 (m, 2H). – ^{13}C NMR (126 MHz, CDCl$_3$): δ = 159.9 (d, J = 248.6 Hz, Cq), 131.7 (d, J = 7.6 Hz), 129.9 (d, J = 8.3 Hz), 124.2, 116.0 (d, J = 21.1 Hz), 115.8 (d, J = 22.5 Hz). – ^{19}F NMR (500 MHz, CDCl$_3$): δ = -114.8. – MS (EI, positive mode) *m/z* (rel intensity) 190 (*[M]*$^+$, 100). *For previous characterization of **4f-o**, see Ref. 10.*

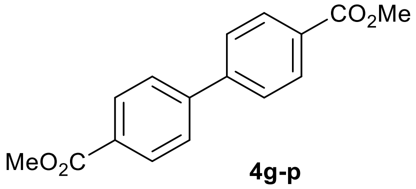

4g-p

Dimethyl biphenyl-4,4'-dicarboxylate (4g-p) - CAS 792-74-5: The biaryl **4g-p** was obtained from 4-methoxycarbonylphenylboronic acid. – Yields 48% (conditions at 65 C°) and 59% (conditions at 25 C°). – ^{1}H NMR (300 MHz, CDCl$_3$): δ = 8.13 (d, J = 8.3 Hz, 4H), 7.69 (d, J = 8.3 Hz, 4H), 3.95 (s, 6H). *For previous characterization of **4g-p**, see Ref. 5.*

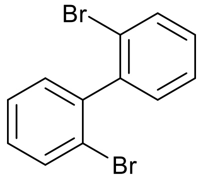

4h-o

2,2'-Dibromo-1,1,'-biphenyl (4h-o) - CAS 13029-09-9: From 2-bromophenylboronic acid, the expected biaryl **4h-o** was isolated as a white solid. – Yields 14% (conditions at 65 C°) and 46% (conditions at 25 C°). – R_f = 0.77 (70:30 cyclohexane/EtOAc). – FTIR-ATR (neat) 3055, 2955, 2922, 2852, 1919, 1799, 1733, 1698, 1631, 1616, 1583, 1560, 1453, 1421, 1264, 1255, 1159,1120, 1074, 1044, 1024, 1001 cm^{-1}. – ^{1}H NMR (500 MHz, CDCl$_3$): δ = 7.69-7.67 (m, 2H), 7.40-7.37 (m, 2H), 7.28-7.24 (m, 4H). – ^{13}C NMR (126 MHz, CDCl$_3$): δ = 142.2 (Cq), 132.7, 131.1, 129.5, 127.3, 123.6 (Cq). – MS (ESI, positive mode) *m/z* (rel intensity) 312 (*[M]*$^+$, 100), 310 (46), 311 (7), 313 (14), 314 (50), 315 (7). *For previous characterization of **4h-o**, see Ref. 6.*

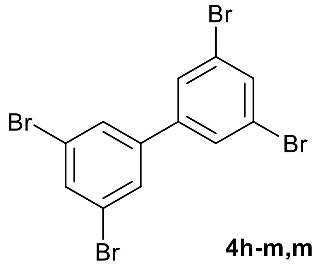

4h-m,m

3,3',5,5'-Tetrabromo-1,1'-biphenyl (4h-m,m) – CAS 16400-50-3: From 3,5-dibromophenylboronic acid, the expected biaryl **4h-m,m** was isolated as a white solid. – Yield 88%. – R_f = 0.80 (70:30 cyclohexane/EtOAc). – FTIR-ATR (neat) 3100, 3064, 2921, 2851, 1775, 1727, 1576, 1539, 1404, 1385, 1362, 1096, 1066, 986 cm^{-1}. – ^{1}H NMR (500 MHz, CDCl$_3$): δ = = 7.701-7.693 (m, 2H), 7.593-7.589 (m, 4H). – ^{13}C NMR (126 MHz, CDCl$_3$): δ = 141.9 (Cq), 134.0, 129.1, 123.7 (Cq). – MS (ESI, positive mode) *m/z* (rel intensity) 470 (*[M]*$^+$, 100), 466 (20), 468 (72), 469 (12), 471 (20), 472 (72), 473 (12), 474 (20). *For previous characterization of **4h-m,m**, see Ref. 4.*

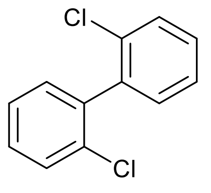

4i-o

2,2'-Dichloro-1,1,'-biphenyl (4i-o) - CAS 13029-08-8: From 2-chlorophenylboronic acid, the expected biaryl **4i-o** was isolated as a white solid. – Yields 64% (conditions at 65 C°) and 66% (conditions at 25 C°). – R_f = 0.82 (70:30 cyclohexane/EtOAc). – FTIR-ATR (neat) 3057, 2957, 2923, 2854, 1963, 1927, 1808, 1728, 1626, 1566, 1487, 1461, 1422, 1261, 1241, 1159, 1127, 1084, 1055, 1026, 1004, 946 cm^{-1}. – ^{1}H NMR (500 MHz, $CDCl_3$): δ = 7.51-7.47 (m, 2H), 7.37-7.32 (m, 4H), 7.30-7.27 (m, 2H). – ^{13}C NMR (126 MHz, $CDCl_3$): δ = 138.5 (Cq), 133.6, 131.3, 129.6, 129.4, 126.6. – MS (ESI, positive mode) *m/z* (rel intensity) 223 (*[M]*$^+$, 100). *For previous characterization of **4i-o**, see Ref. 4.*

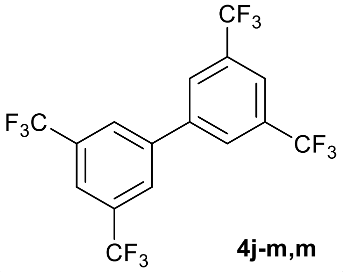

3,3',5,5'-Tetrakis(trifluoromethyl)-1,1'-biphenyl (4j-m,m) - CAS 396-44-1: From 3,5-bis(trifluoromethyl)phenylboronic acid, the expected biaryl **4j-m,m** was isolated as a white solid. – Yield 70%. – R_f = 0.48 (cyclohexane). – FTIR-ATR (neat) 3097, 2927, 1616, 1457, 1348, 1275, 1175, 1119, 1104, 1072, 929 cm^{-1}. – ^{1}H NMR (300 MHz, $CDCl_3$): δ = 7.69 (bs, 4H), 7.59 (bs, 2H). – ^{13}C NMR (126 MHz, $CDCl_3$): δ = 140.6 (Cq), 133.0 (q, J = 33.8 Hz, Cq), 127.7 (q, J = 2.9 Hz), 125.3 (q, J = 273.0 Hz, Cq), 122.8 (sept, J = 3.8 Hz). – ^{19}F NMR (500 MHz, $CDCl_3$): δ = -62.9. – MS (EI, positive mode) *m/z* (rel intensity) 426 (*[M]*$^+$, 100). *For previous characterization of **4j-m,m**, see Ref. 8.*

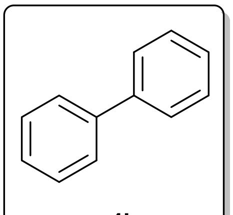

4k

Biphenyl (4k) - CAS 92-52-4: From phenylboronic acid, the expected biphenyl **4k** was isolated as a white solid. – Yield 26%. – FTIR-ATR (neat) 3062, 3034, 2925, 2853, 1958, 1884, 1597, 1569, 1479, 1429, 1344, 1265, 1181, 1170, 1111, 1091, 1076, 1041, 1006 cm^{-1}. – ^{1}H NMR (300 MHz, $CDCl_3$): δ = 7.62-7.56 (m, 4H), 7.49-7.40 (m, 4H), 7.39-7.29 (m, 2H). – ^{13}C NMR (126 MHz, $CDCl_3$): δ = 141.2 (Cq), 128.8, 127.3, 127.2. *For previous characterization of **4k**, see Ref. 4.*

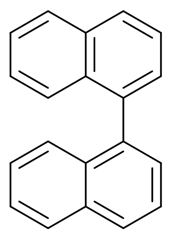

4l

1,1'-Binaphthalene (4l) - CAS 604-53-5: From 1- naphthylboronic acid, the expected biphenyl **4l** was isolated as a white solid. – Yields 54% (conditions at 65 C°) and 73% (conditions at 25 C°). – R_f = 0.48 (cyclohexane). – FTIR-ATR (neat) 3040, 1586, 1504, 1378, 1327, 1256, 1211, 1199, 1178, 1162, 1130, 1013, 968 cm^{-1}. – ^{1}H NMR (300 MHz, $CDCl_3$): δ = 7.97-7.95 (m, 4H), 7.62-7.59 (m, 2H), 7.51-7.47 (m, 4H), 7.41-7.40 (m, 2H), 7.31-7.28 (m, 2H). – ^{13}C NMR (126 MHz, $CDCl_3$): δ = 138.6, 133.6, 133.0, 128.3, 128.0, 127.9. 126.7, 126.1, 125.9, 125.5. – MS (ESI, positive mode) *m/z* (rel intensity) 254 (*[M]*$^+$, 100). *For previous characterization of **4l**, see Ref. 6.*

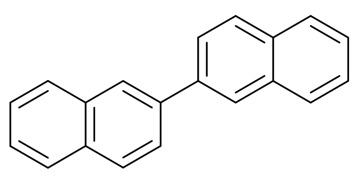

4m

2,2'-Binaphthalene (4m) - CAS 612-78-2: From 2-naphthylboronic acid, the expected biaryl **4m** was isolated as a white solid. – Yields 41% (conditions at 65 C°) and 85% (conditions at 25 C°). – R_f = 0.74 (70:30 cyclohexane/EtOAc). – FTIR-ATR (neat) 3053, 2921, 2852, 1593, 1461, 1376, 1259, 1129, 1093, 1021 cm^{-1}. – ^1H NMR (500 MHz, $CDCl_3$): δ = 8.18 (s, 2H), 7.98-7.88 (m, 8H), 7.49-7.55 (m, 4H). – ^{13}C NMR (126 MHz, $CDCl_3$): δ = 138.5, 133.9, 132.8, 128.7, 128.4, 127.8, 126.5, 126.3, 126.2, 125.9. – MS (ESI, positive mode) *m/z* (rel intensity) 254 (*[M]*$^+$, 100). *For previous characterization of* ***4m****, see Ref. 4.*

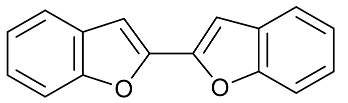

4n

2,2'-Bibenzofuran (4n) - CAS 41014-29-3: From benzofuran-3-ylboronic acid, the expected 2,2'-bibenzofuran **4n** was isolated as a white solid. – Yields 31% (conditions at 65 C°) and 48% (conditions at 25 C°). – R_f = 0.81 (70:30 cyclohexane/EtOAc). – FTIR-ATR (neat) 3158, 3122, 3065, 3056, 3034, 3016, 1940, 1899, 1863, 1820, 1780, 1625, 1611, 1515, 1467, 1438, 1342, 1299, 1253, 1216, 1170, 1152, 1140, 1102, 1046, 1008 cm^{-1}. – ^1H NMR (500 MHz, $CDCl_3$): δ = 7.65-7.63 (m, 2H), 7.56-7.54 (m, 2H), 7.35–7.32 (m, 2H), 7.29–7.26 (m, 2H), 7.17-7.16 (m, 2H). – ^{13}C NMR (126 MHz, $CDCl_3$): δ = 155.2, 147.8, 128.7, 125.2, 123.5, 121.5, 111.4, 103.8. – MS (ESI, positive mode) *m/z* (rel intensity) 234 (*[M]*$^+$, 100). *For previous characterization of* ***4m****, see Ref. 4.*

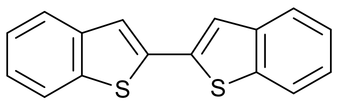

4o

2,2'-Dibenzothiophene (4o) - CAS 65689-53-4: From benzo[b]thiophen-2-ylboronic acid, the expected 2,2'-dibenzothiophene **4o** was isolated as a white solid. – Yields 13% (conditions at 65 C°) and 25% (conditions at 25 C°). – R_f = 0.76 (70:30 cyclohexane/EtOAc). – FTIR-ATR (neat) 3051, 3027, 2958, 2918, 2850, 1418, 1249 cm^{-1}. – ^1H NMR (300 MHz, $CDCl_3$): δ = 7.82-7.77 (m, 4H), 7.52 (s, 2H), 7.38-7.32 (m, 4H). – ^{13}C NMR (126 MHz, $CDCl_3$): δ = 140.3, 139.6, 137.3, 125.1, 124.9, 123.9, 122.3, 121.5. – MS (ESI, positive mode) *m/z* (rel intensity) 266 (*[M]*$^+$, 100). *For previous characterization of* ***4o****, see Ref. 11.*

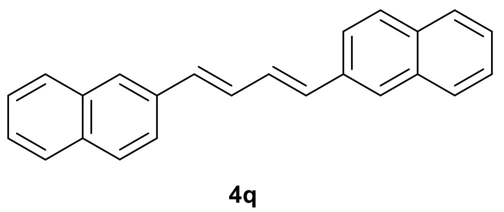

4q

(1*E*,3*E*)-1,4-Diphenylbuta-1,3-diene (4q) - CAS 538-81-8: From (*E*)-styrylboronic acid, the expected 1,3'-diene **4q** was isolated as a white solid. – Yields 34% (conditions at 65 C°) and 43% (conditions at 25 C°). – R_f = 0.31 (cyclohexane). – FTIR-ATR (neat) 3078, 3054, 3015, 2924, 2854, 1952, 1870, 1731, 1592, 1571, 1488, 1443, 1292, 1176, 1073, 983, 912 cm^{-1}. – ^1H NMR (500 MHz, $CDCl_3$): δ = 7.47-7.44 (m, 4H), 7.36-7.31 (m, 4H), 7.26–7.22 (m, 2H), 7.00-6.94 (m, 2H), 6.71-6.65 (m, 2H). – ^{13}C NMR (126 MHz, $CDCl_3$): δ = 137.5 (Cq), 133.0, 129.4, 128.8, 127.7,

126.5. – MS (ESI, positive mode) *m/z* (rel intensity) 206 (*[M]*$^+$, 100). *For previous characterization of **4o**, see Ref. 10.*

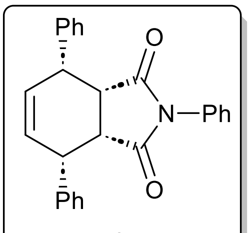

6

2,4,7-Triphenyl-3a,4,7,7a-tetrahydro-1*H*-isoindole-1,3(*2H*)-dione (6) - CAS 20929-47-9: From (*E*)-styrylboronic acid, the expected cycloadduct **6** was isolated as a white solid. – Yields 36% (one-pot process in PhMe) and 31% (one-pot process in MeOH) – R_f = 0.37 (70:30 cyclohexane/EtOAc). – FTIR-ATR (neat) 3062, 3029, 2946, 2835, 1780, 1705, 1596, 1495, 1453, 1374, 1165, 750, 702, 673, 618, 576, 495 cm^{-1}. – ^{1}H NMR (500 MHz, $CDCl_3$): δ = 7.45-7.24 (m, 13H), 7.06-6.94 (m, 2H), 6.60–6.48 (m, 2H), 3.93 (d, *J* = 4.9 Hz, 2H), 3.66 (dd, *J* = 4.9 Hz, *J* = 2.2 Hz, 2H). – ^{13}C NMR (126 MHz, $CDCl_3$): δ = 175.1, 139.3, 131.8, 131.6, 129.1, 129.0, 128.6, 128.5, 127.4, 126,5, 46.7, 41.9. – MS (ESI, positive mode) *m/z* (rel intensity) 380 (*[M+H]*$^+$, 100). *For previous characterization of **6**, see Ref. 12.*

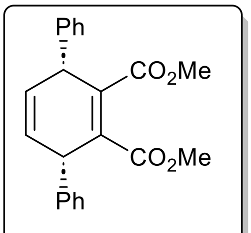

7

Dimethyl 3,6-diphenylcyclohexa-1,4-diene-1,2-dicarboxylate (7) - CAS 49538-77-4: From (*E*)-styrylboronic acid, the expected cycloadduct **7** was isolated as a colorless solid. – Yields 31% (one-pot process in PhMe) and 23% (one-pot process in MeOH) – R_f = 0.52 (70:30 cyclohexane/EtOAc). – FTIR-ATR (neat) 1740, 1650, 1610, 1500, 1450-1400, 1265, 1150, 1000, 855, 795, 760, 750, 740, 700 cm^{-1}. – ^{1}H NMR (300 MHz, $CDCl_3$): δ = 7.40-7.26 (m, 10H), 5.80-5.77 (m, 2H), 4.48–4.52 (m, 2H), 3.56 (s, 6H). – ^{13}C NMR (126 MHz, $CDCl_3$): δ = 167.9 (Cq), 141.2, 135.6, 128.7, 128.3, 127.1, 126.0, 52.0, 44.0. – MS (ESI, positive mode) *m/z* (rel intensity) 348 (*[M]*$^+$, 100). *For previous characterization of **7**, see Ref. 13.*

REFERENCES

1- Esguerra, K. V. N.; Fall, Y.; Petitjean, L.; Lumb, J.-P. Controlling the Catalytic Aerobic Oxidation of Phenols. *J. Am. Chem. Soc.* **2014**, *136*, 7662-7668.

2- Hossain, Md. A.; Akiyama, K.; Sugiura, K.-i. Pyren-2-yl-Substituted Biphenoquinone and *p*-Benzoquinone: Pyren-2-yl as a Weak Electron-Withdrawing Substituent. *Chemistry Select* **2016**, *1*, 6859-6865.

3- Kondo, M.; Tada, S.; Shioiri, T.; Nakajima, H.; Nishijima, M.; Honma, T.; Fujiwara, K. Aerobic Oxidative Coupling of 2-Naphthols Catalyzed by Flame-Made VO_x/*t*-ZrO_2: Effect of Metal Oxide Support and VO_x Content on Catalytic Activity. *Appl. Catal. A: Gen.* **2024**, *676*, 119638.

4- Kirai, N.; Yamamoto, Y. Homocoupling of Arylboronic Acids Catalyzed by 1,10-Phenanthroline-Ligated Copper Complexes in Air. *Eur. J. Org. Chem.* **2009**, *12*, 1864-1867.

5- Appa, R. M.; Lakshmidevi, J.; Naidu, B. R.; Venkateswarlu, K. Pd-Catalyzed Oxidative Homocoupling of Arylboronic Acids in WEPA: A Sustainable Access to Symmetrical Biaryls under Added Base and Ligand-Free Ambient Conditions. *Mol. Catal.* **2021**, *501*, 111366.

6- Minus, M. B.; Moor, S. R.; Pary, F. F.; Nirmani, L. P. T.; Chwatko, M.; Okeke, B.; Singleton, J. E.; Nelson, T. L.; Lynd, N. A.; Anslyn, E. V. "benchtop" Biaryl Coupling Using Pd/Cu Cocatalysis: Application to the Synthesis of Conjugated Polymers. *Org. Lett.* **2021**, *23*, 2873-2877.

7- Das, S. K.; Chandra, B. K.; Molla, R. A.; Sengupta, M.; Islam, S. M.; Majee, A.; Bhaumik, A. CuO Grafted Triazine Functionalized Covalent Organic Framework as An Efficient Catalyst for C-C Homocoupling Reaction. *Mol. Catal.* **2020**, *480*, 110650.

8- Cao, Y.-N.; Tian, X.-C.; Chen, X.-X.; Yao, Y.-X.; Gao, F.; Zhou, X.-L. Rapid Ligand-Free Base-Accelerated Copper-Catalyzed Homocoupling Reaction of Arylboronic Acids. *Synlett* **2017**, *28*, 601-606.

9- Lin, C.-N.; Huang, C.-Y.; Yu, C.-C.; Chen, Y.-M.; Ke, W.-M.; Wang, G.-J.; Lee, G.-A.; Shieh, M. Iron Carbonyl Cluster-Incorporated Cu(I) NHC Complexes in Homocoupling of Arylboronic Acids: An Effective $[TeFe_3(CO)_9]^{2-}$ Ligand. *Dalton Trans.* **2015**, *44*, 16675-16679.

10- Kaboudin, B.; Mostafalu, R.; Yokomatsu, T. Fe_3O_4 Nanoparticle-Supported Cu(II)-*β*-Cyclodextrin Complex as a Magnetically Recoverable and Reusable Catalyst for the Synthesis of Symmetrical Biaryls and 1,2,3-Triazoles from Aryl Boronic Acids. *Green Chem.* **2013**, *15*, 2266-2274.

11- Ostrowska, S.; Rogalski, S.; Lorkowski, J.; Walkowiak, J.; Pietraszuk, C. Effective Homocoupling of Aryl- and Alkenylboronic Acids in the Presence of Low Loadings of $[\{Pd(\mu–OH)Cl(IPr)\}_2]$. *Synlett* **2018**, *29*, 1735-1740.

12- Iosub, A. V.; Stahl, S. S. Palladium-Catalyzed Aerobic Oxidative Dehydrogenation of Cyclohexenes to Substituted Arene Derivatives. *J. Am. Chem. Soc.* **2015**, *137*, 3454-3457.

13- Thirion, D.; Poriel, C.; Rault-Berthelot, J.; Barrière, F.; Jeannin, O. (2,1-*a*)-Indenofluorene Derivatives: Syntheses, X-ray Structures, Optical and Electrochemical Properties. *Chem. Eur. J.* **2010**, *16*, 13646-13658.

COMPILATION OF NMR SPECTRA

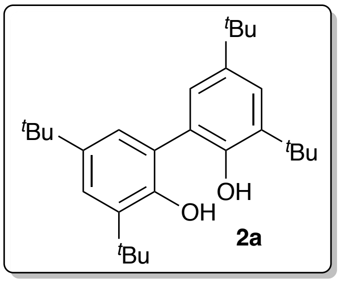

2a

^{1}H-NMR in $CDCl_3$

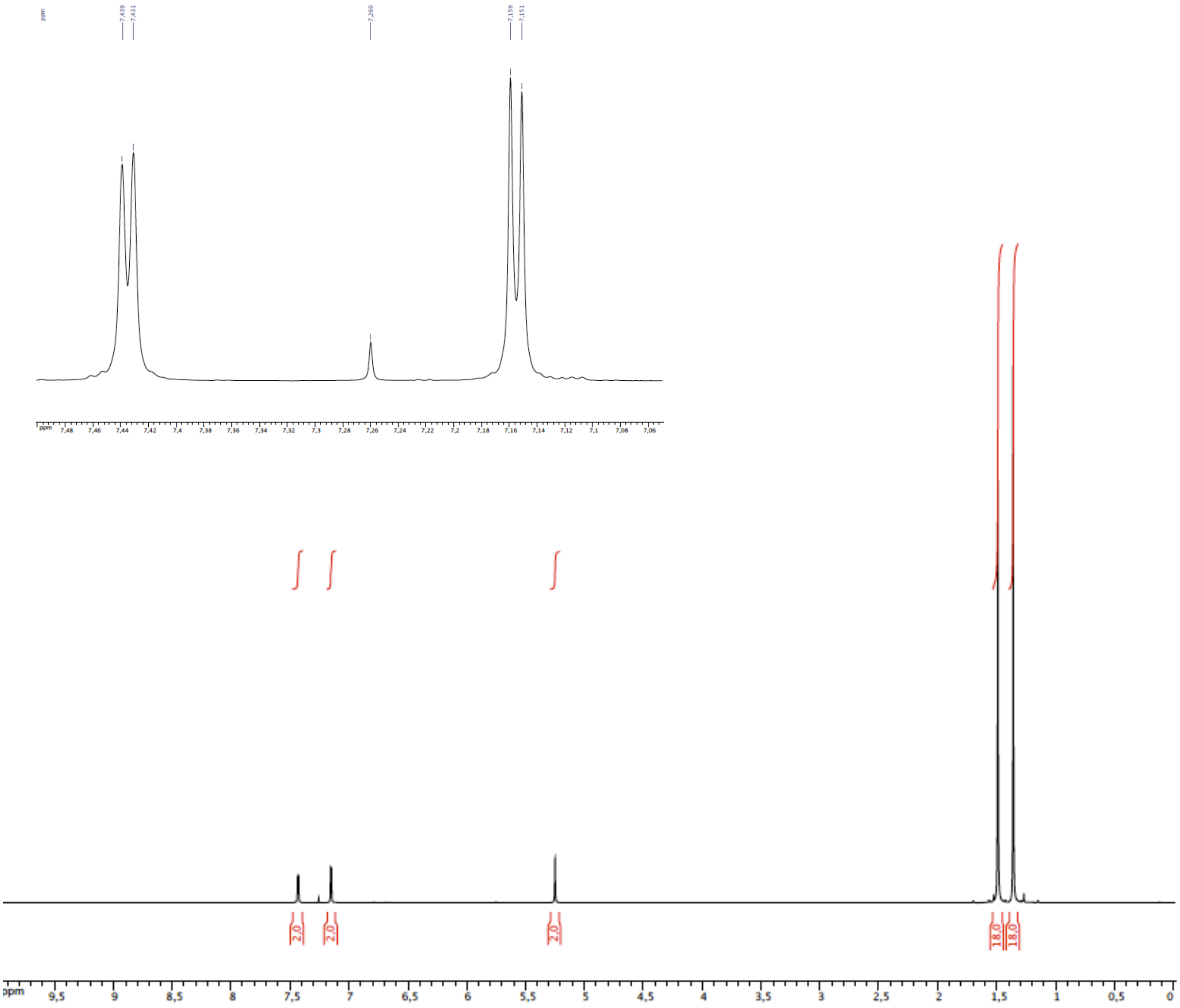

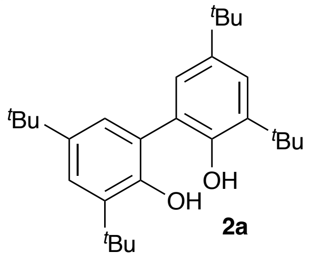
2a

^{1}H-NMR in $CDCl_3$ – list of peaks

Label	Intensity	Frequency(point)	Frequency(ppm)	Frequency(Hz)
1	87652896	28631	7,439	2233,048
2	92407184	28656	7,431	2230,687
3	15554727	29200	7,260	2179,32
4	122908464	29520	7,159	2149,104
5	117192328	29546	7,151	2146,649
6	147823952	35580	5,253	1576,891
7	1418354176	47533	1,493	448,233
8	1792464768	47948	1,363	409,047

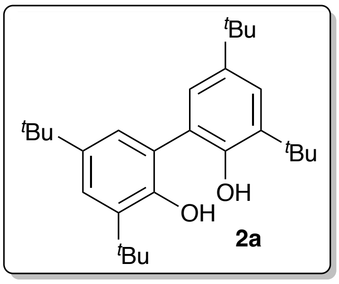

^{13}C NMR in $CDCl_3$

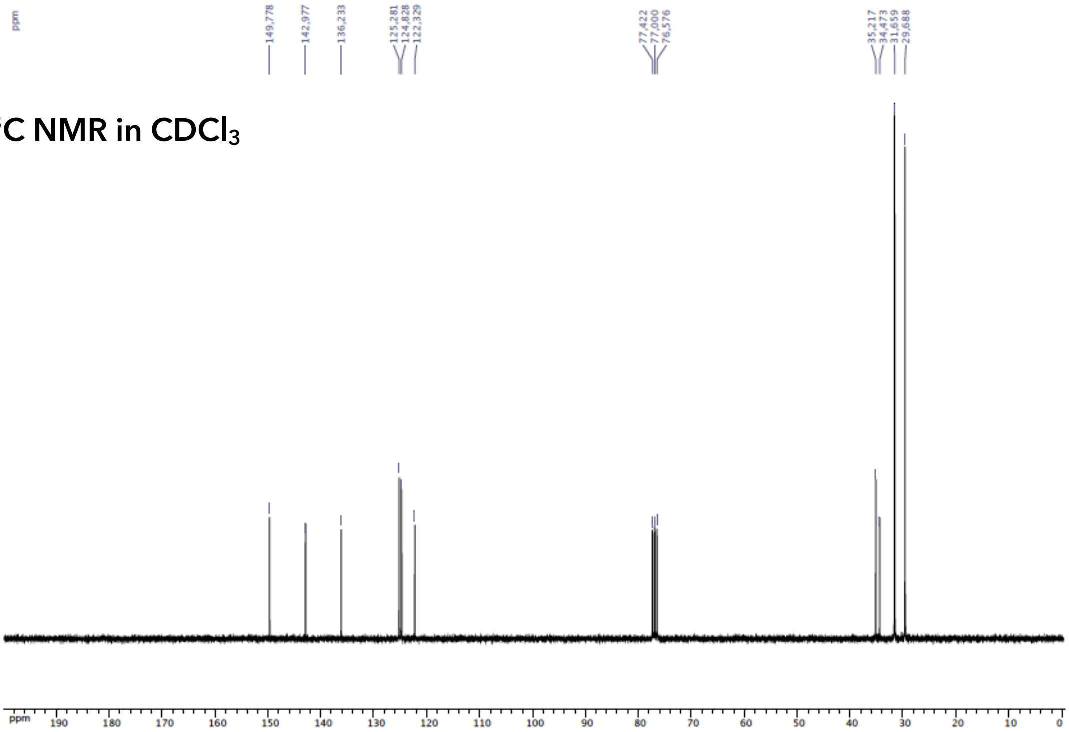

DEPT135 in $CDCl_3$

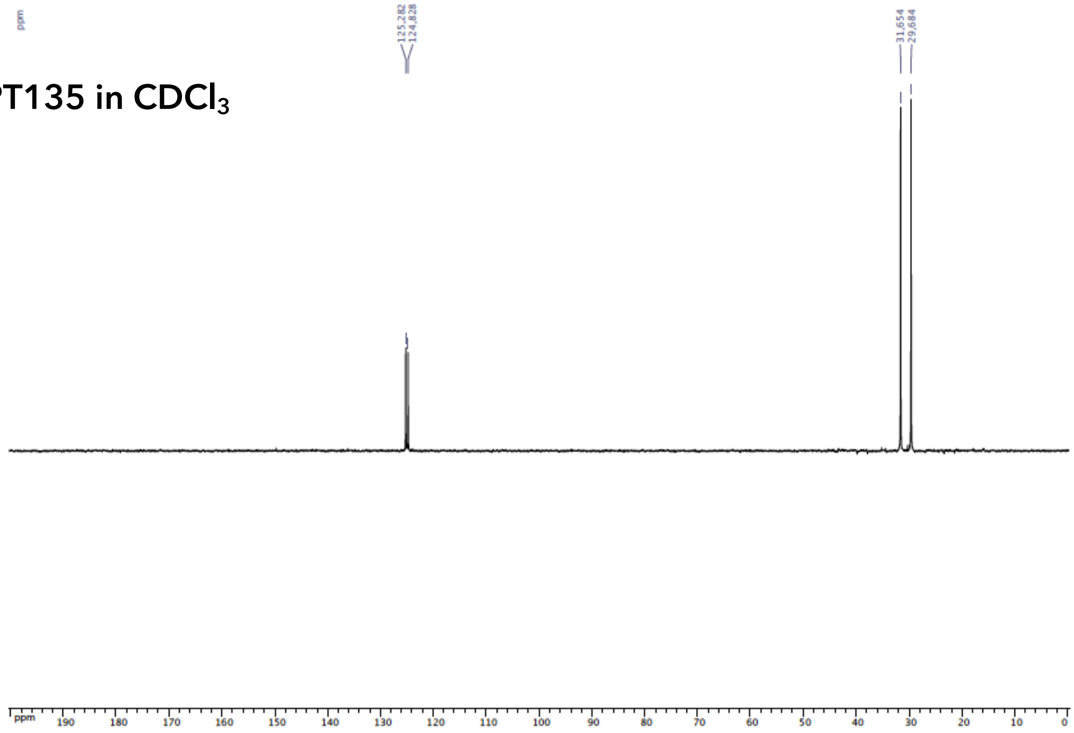

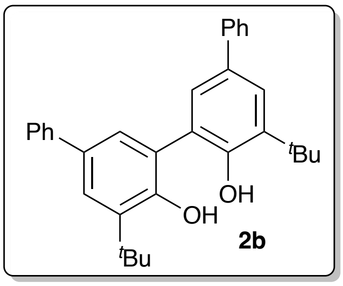

^{1}H-NMR in $CDCl_3$

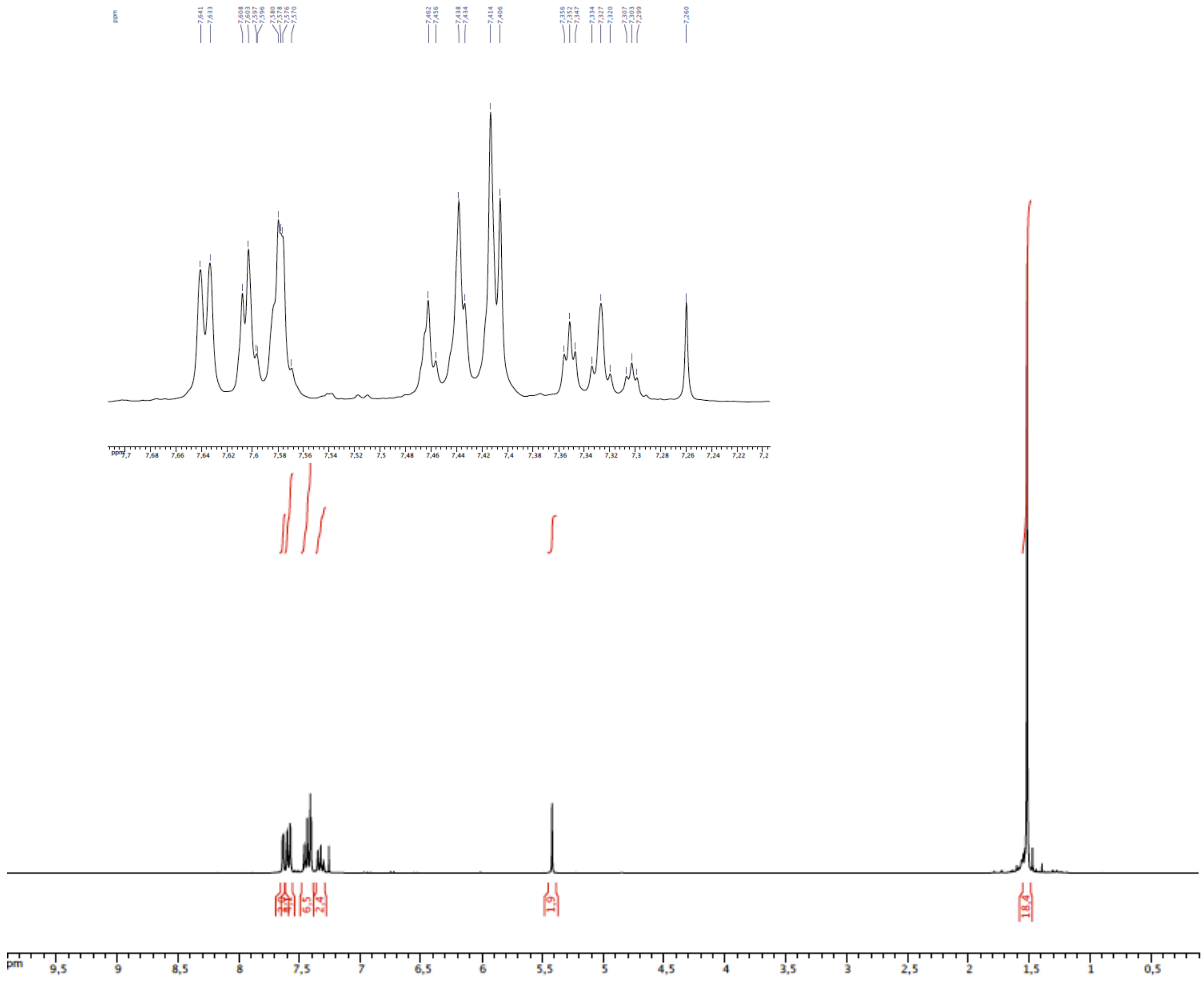

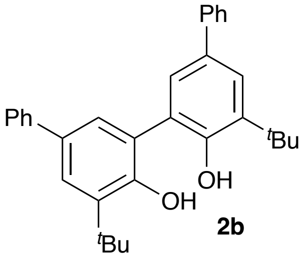

2b

^{1}H-NMR in $CDCl_3$ – list of peaks

Label	Intensity	Frequency(point)	Frequency(ppm)	Frequency(Hz)
1	85834760	27989	7,641	2293,668
2	90134968	28013	7,633	2291,402
3	70456504	28094	7,608	2283,754
4	98929104	28109	7,603	2282,338
5	31654328	28129	7,597	2280,449
6	117909776	28183	7,58	2275,35
7	109235312	28189	7,578	2274,784
8	107531712	28194	7,576	2274,311
9	65961768	28557	7,462	2240,035
10	27069152	28576	7,456	2238,241
11	130177024	28633	7,438	2232,859
12	64077540	28648	7,434	2231,443
13	187673984	28712	7,414	2225,399
14	132254544	28736	7,406	2223,133
15	31260440	28896	7,356	2208,025
16	52175640	28909	7,352	2206,798
17	32980966	28923	7,347	2205,476
18	23607538	28965	7,334	2201,51
19	64043640	28987	7,327	2199,433
20	25593302	29064	7,303	2192,162
21	64469212	29200	7,26	2179,32
22	164315376	35035	5,425	1628,352
23	1439661568	47453	1,518	455,787

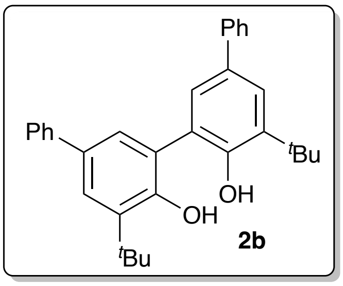

^{13}C NMR in $CDCl_3$

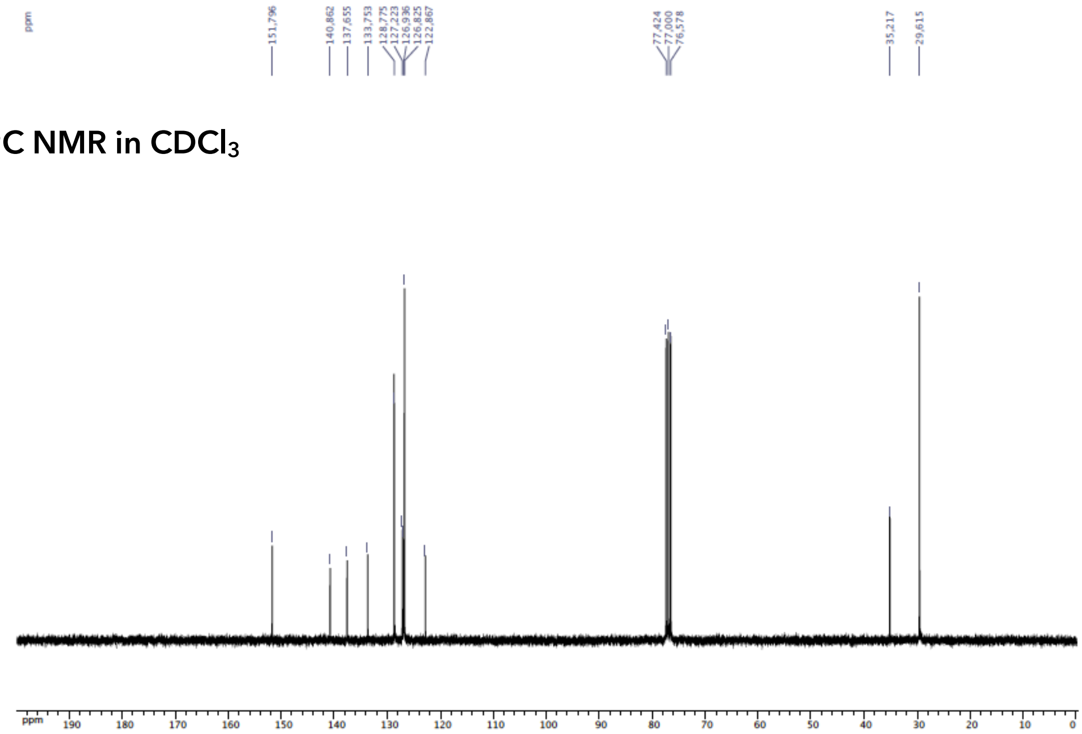

DEPT135 in $CDCl_3$

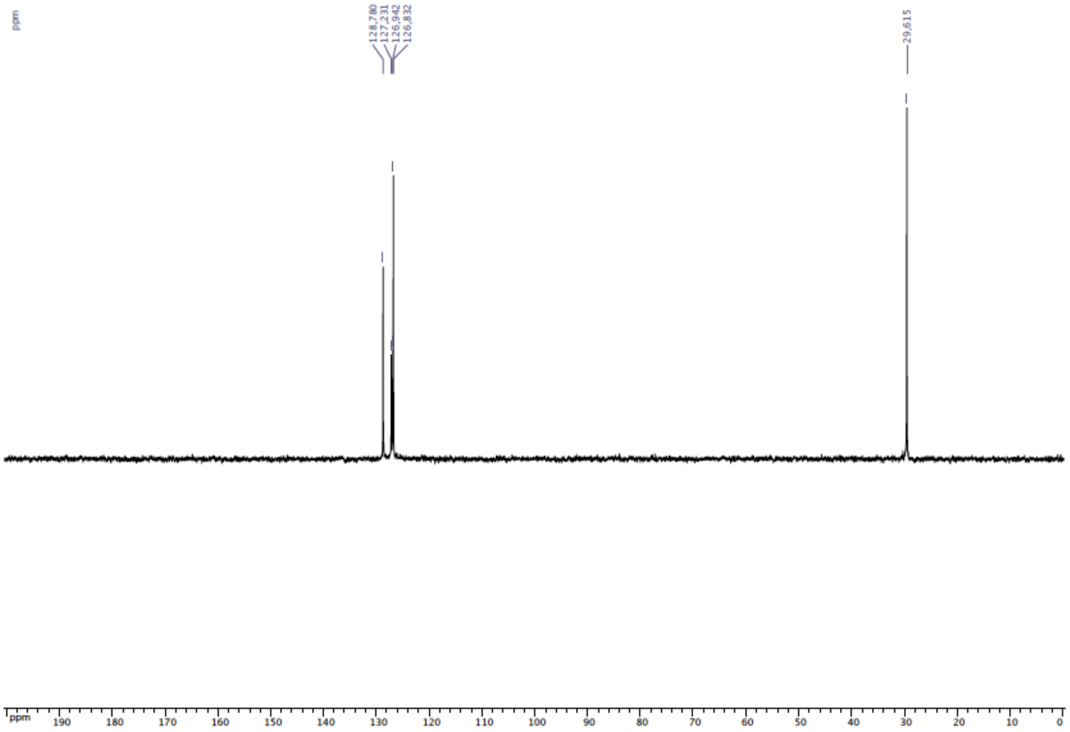

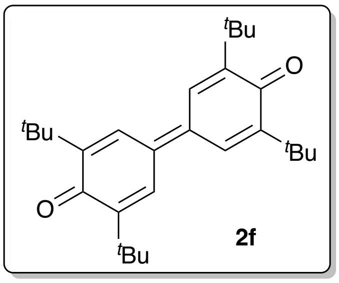

^{1}H-NMR in $CDCl_3$

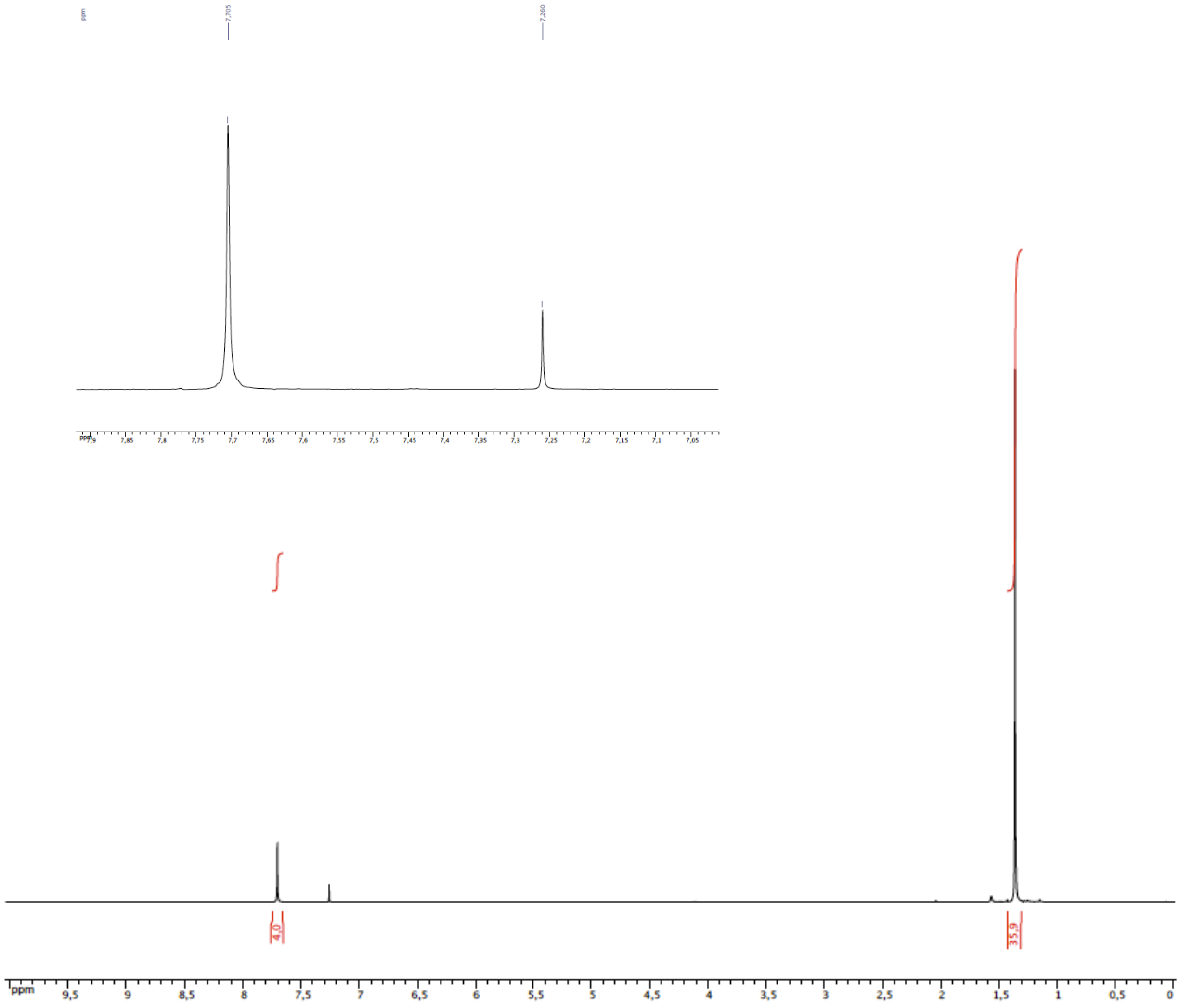

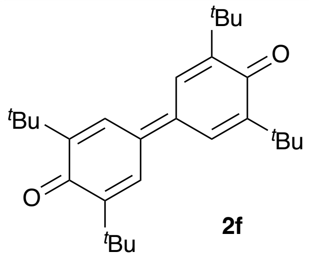

2f

^{1}H-NMR in $CDCl_3$ – list of peaks

Label	Intensity	Frequency(point)	Frequency(ppm)	Frequency(Hz)
1	344234112	27786	7,705	2312,837
2	103685016	29200	7,260	2179,32
3	3139241216	47947	1,363	409,142

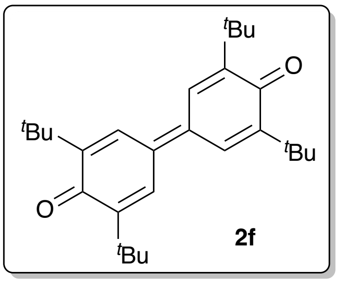

2f

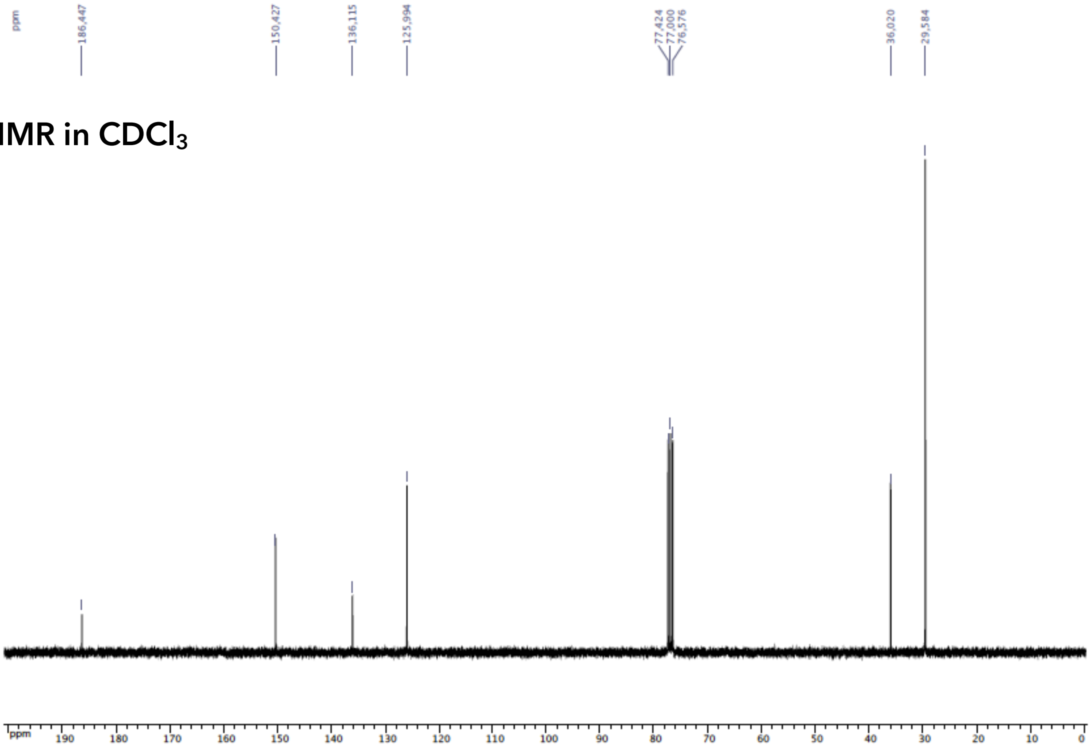

^{13}C NMR in $CDCl_3$

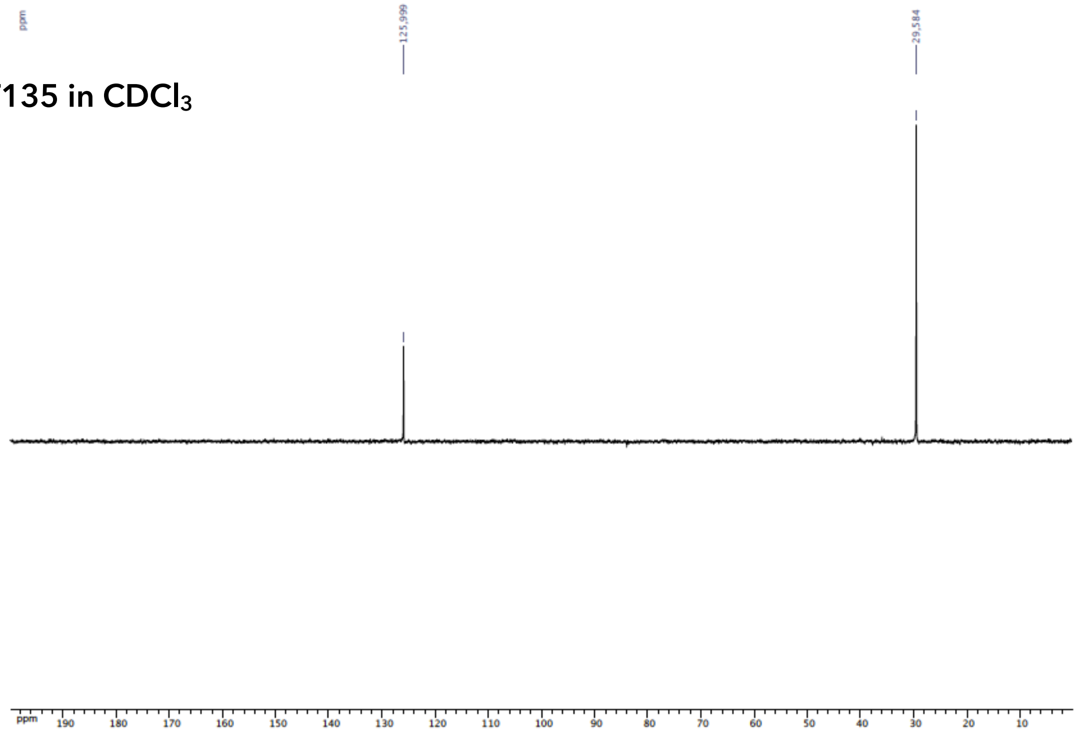

DEPT135 in $CDCl_3$

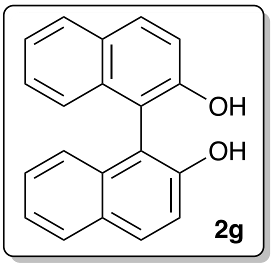

2g

^{1}H-NMR in $CDCl_3$

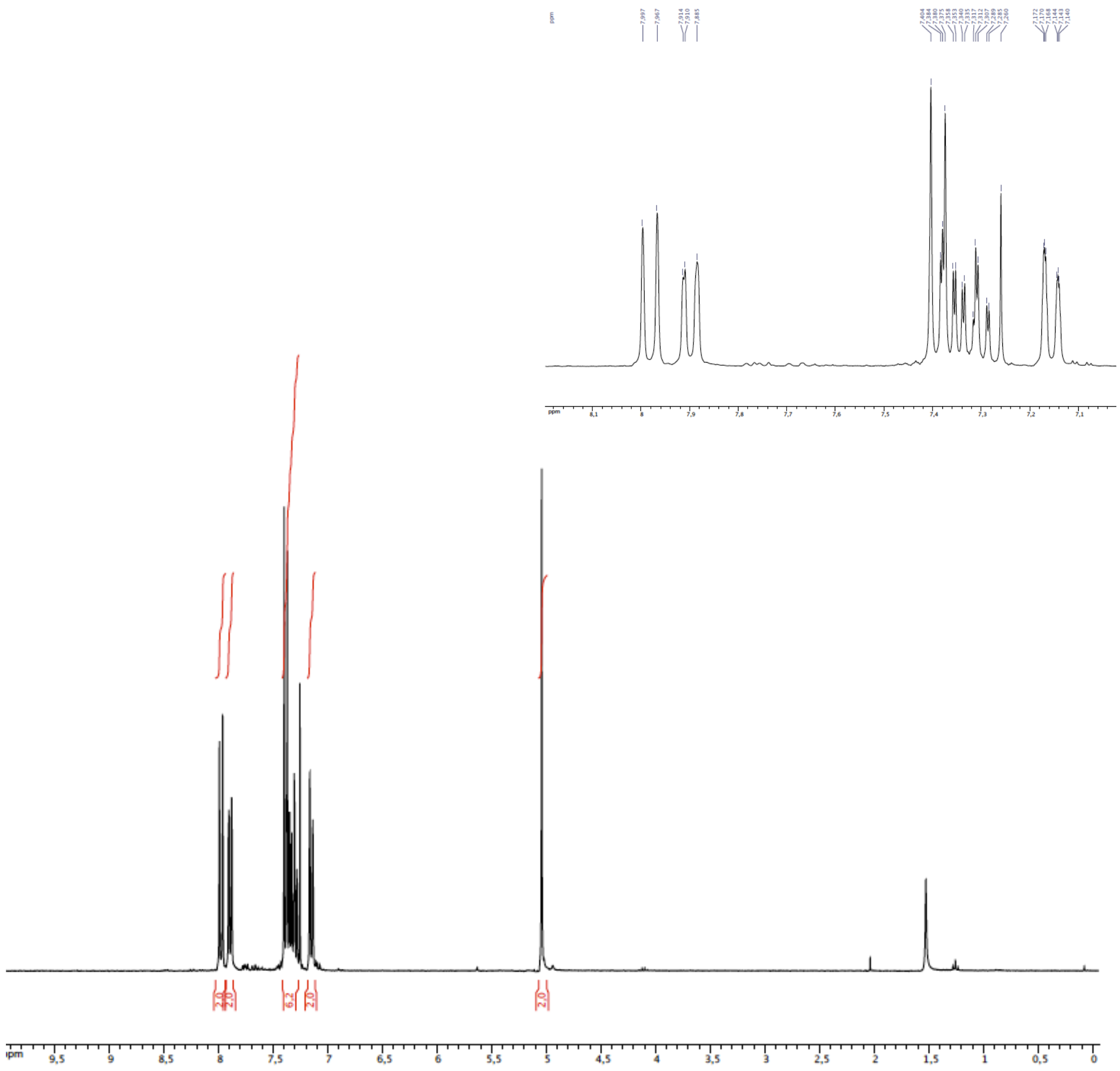

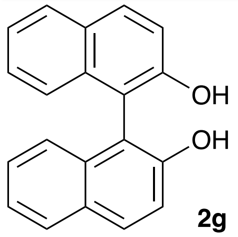

2g

^{1}H-NMR in $CDCl_3$ – list of peaks

Label	Intensity	Frequency(point)	Frequency(ppm)	Frequency(Hz)
1	82582072	26857	7,997	2400,557
2	91507288	26951	7,967	2391,681
3	53227800	27121	7,914	2375,629
4	57870944	27134	7,91	2374,402
5	62453792	27212	7,885	2367,036
6	166686816	28742	7,404	2222,567
7	63327656	28805	7,384	2216,618
8	81991456	28818	7,38	2215,39
9	150790432	28835	7,375	2213,785
10	57115564	28888	7,358	2208,781
11	57189944	28904	7,353	2207,27
12	45769120	28947	7,34	2203,21
13	49692716	28963	7,335	2201,699
14	28194156	29020	7,317	2196,317
15	71015568	29035	7,312	2194,9
16	60720136	29050	7,307	2193,484
17	36360460	29108	7,289	2188,007
18	33303108	29122	7,285	2186,685
19	103338816	29200	7,26	2179,32
20	69016400	29480	7,172	2152,881
21	71016032	29486	7,17	2152,315
22	65743100	29493	7,168	2151,654
23	51621116	29568	7,144	2144,572
24	54413468	29573	7,143	2144,1
25	49304380	29581	7,14	2143,344
26	180285744	36235	5,047	1515,043

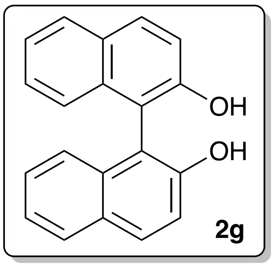

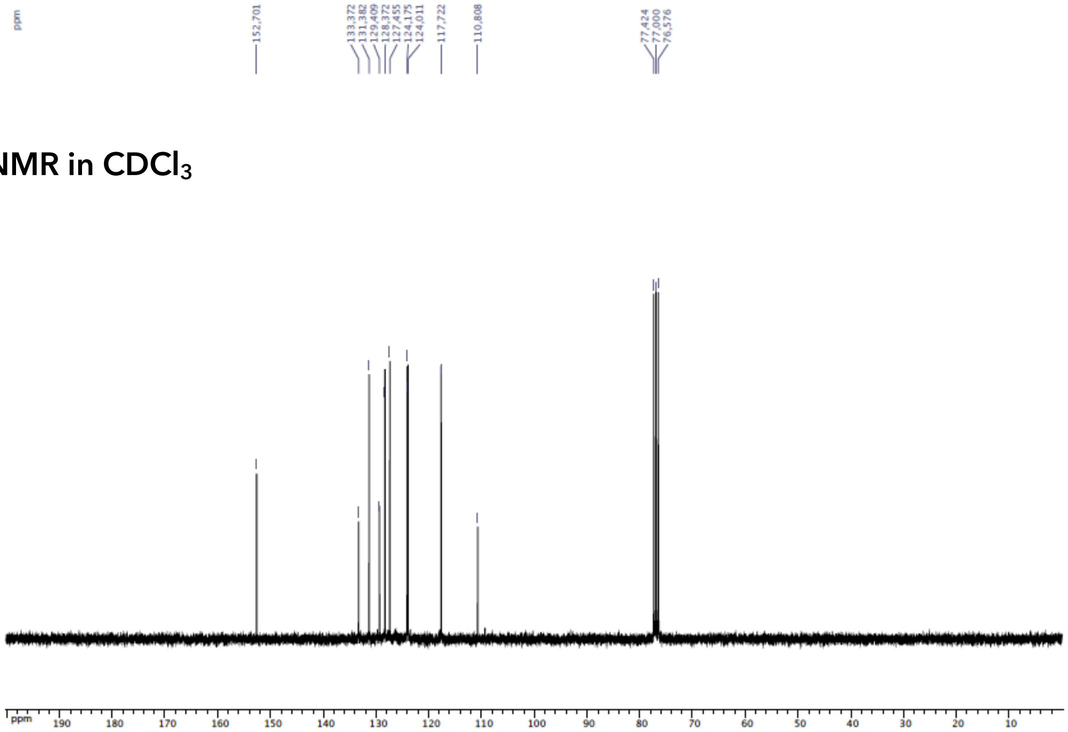

^{13}C NMR in $CDCl_3$

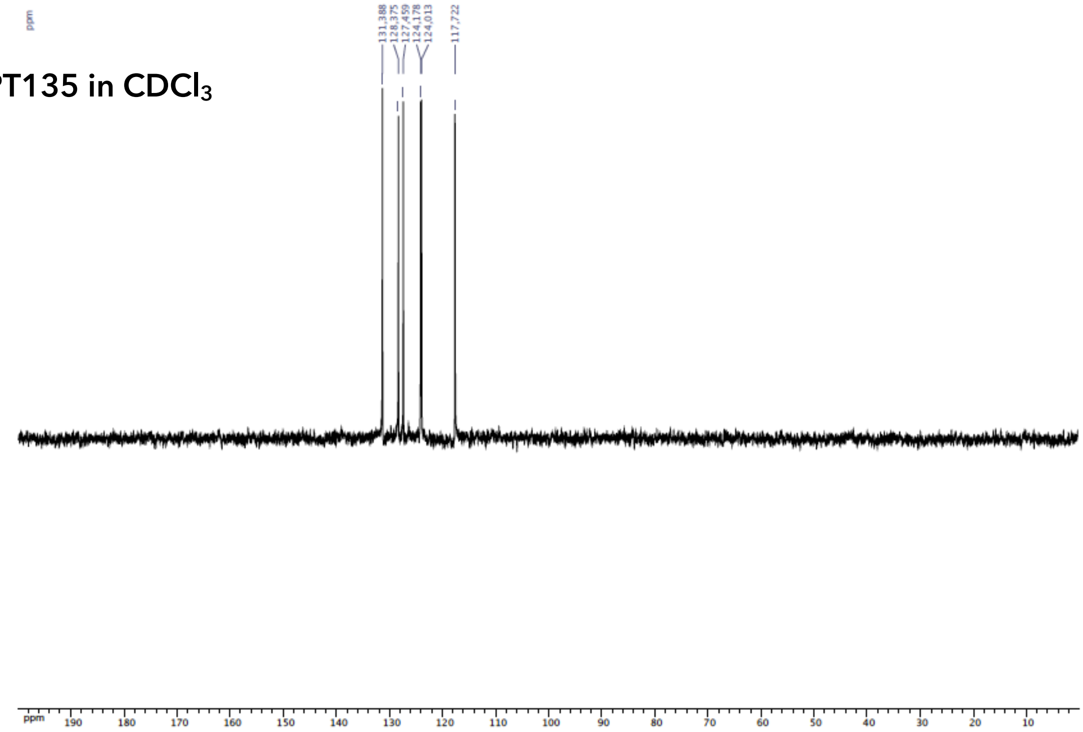

DEPT135 in $CDCl_3$

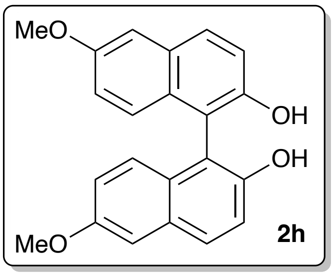

^{1}H-NMR in $CDCl_3$

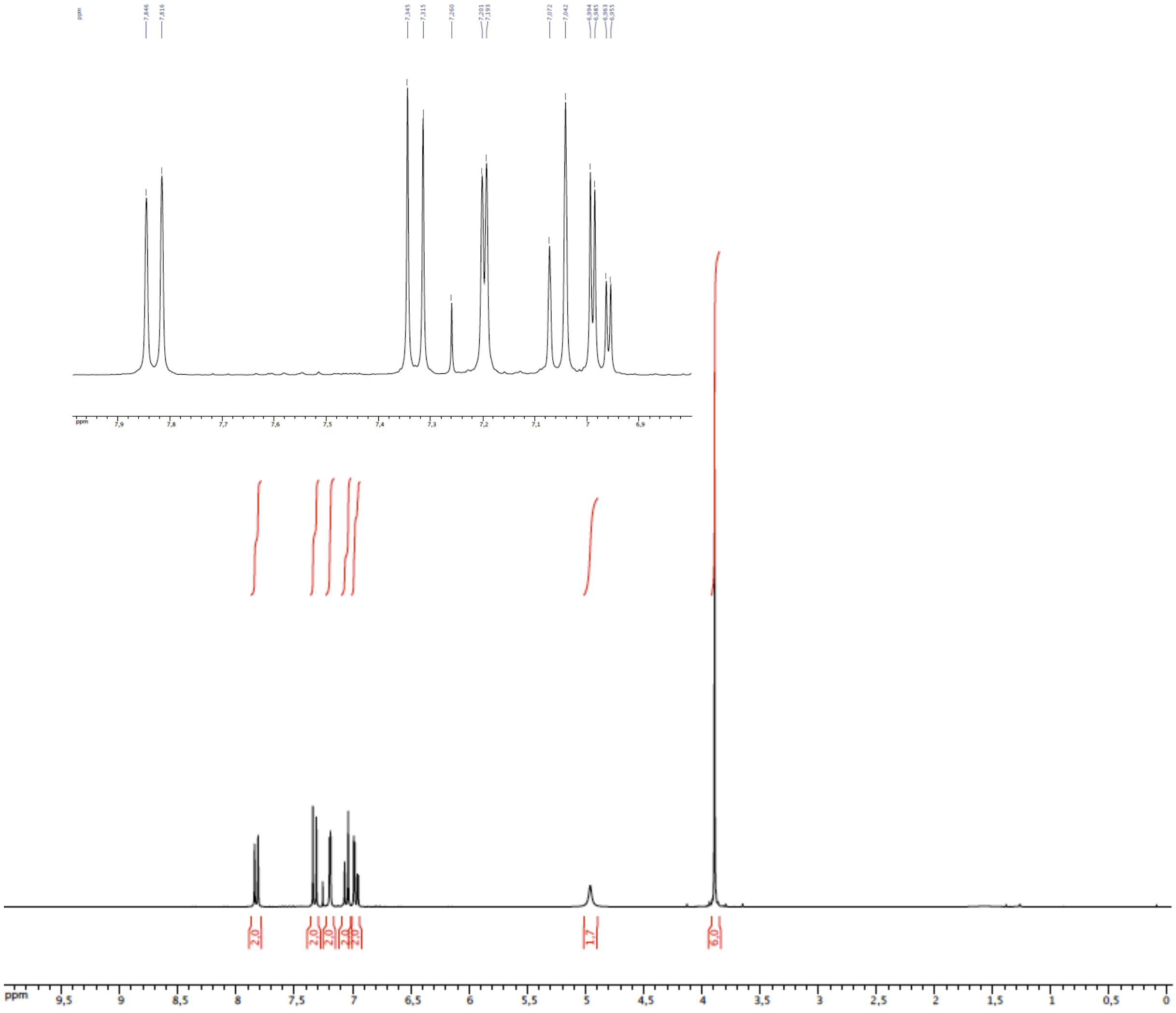

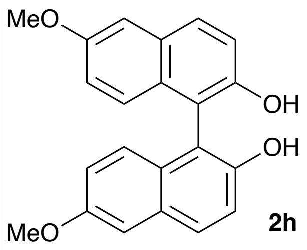

2h

^{1}H-NMR in $CDCl_3$ – list of peaks

Label	Intensity	Frequency(point)	Frequency(ppm)	Frequency(Hz)
1	101837232	27338	7,846	2355,139
2	114383008	27433	7,816	2346,169
3	165351200	28931	7,345	2204,72
4	147613952	29026	7,315	2195,75
5	41534136	29200	7,26	2179,32
6	114344720	29386	7,201	2161,757
7	121671424	29413	7,193	2159,208
8	74316864	29797	7,072	2122,949
9	156953152	29894	7,042	2113,79
10	116791184	30046	6,994	2099,437
11	106736080	30073	6,985	2096,888
12	54145060	30143	6,963	2090,278
13	51926372	30170	6,955	2087,728
14	35106288	36505	4,962	1489,548
15	875405440	39902	3,894	1168,788

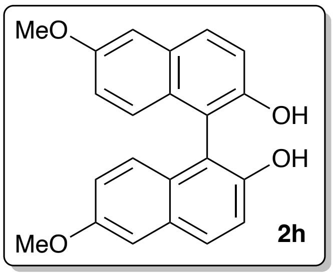

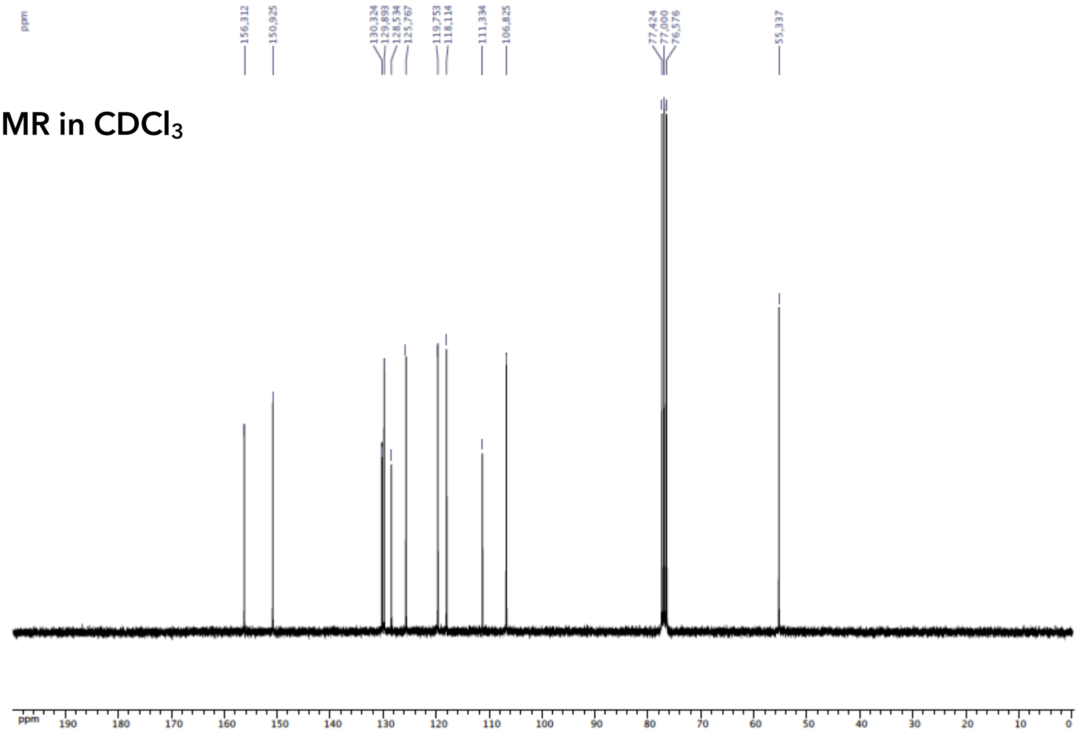

^{13}C NMR in $CDCl_3$

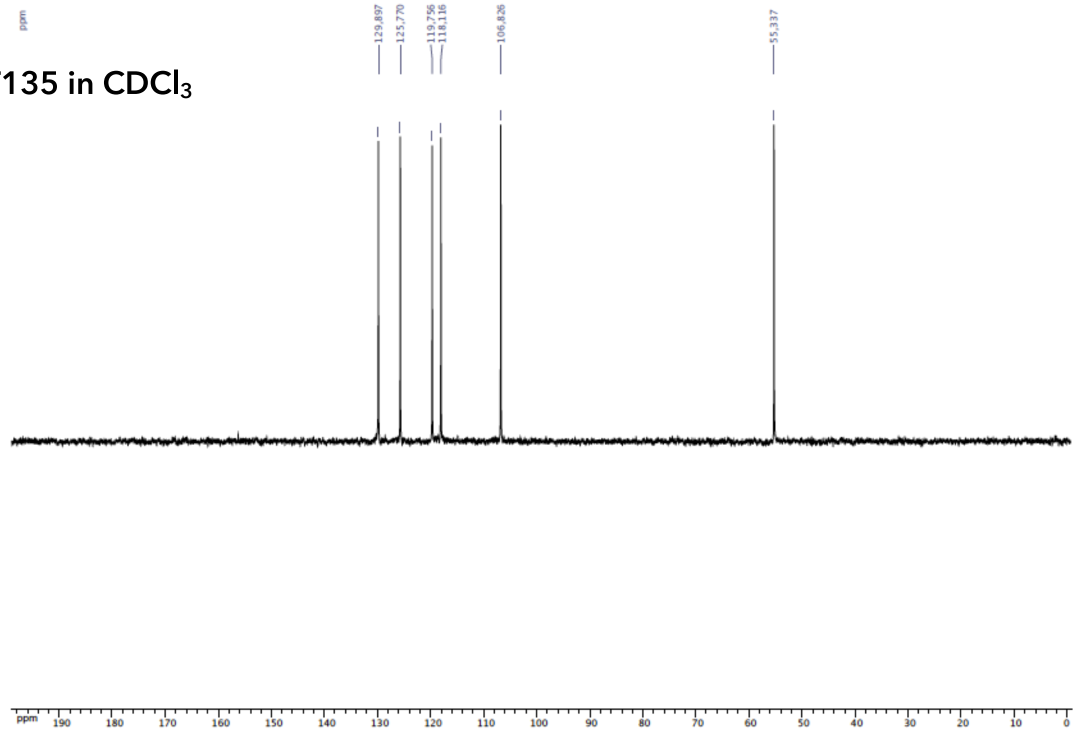

DEPT135 in $CDCl_3$

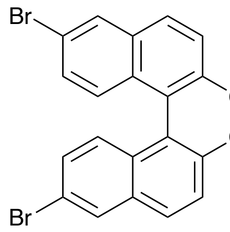

2i

^{1}H-NMR in $CDCl_3$

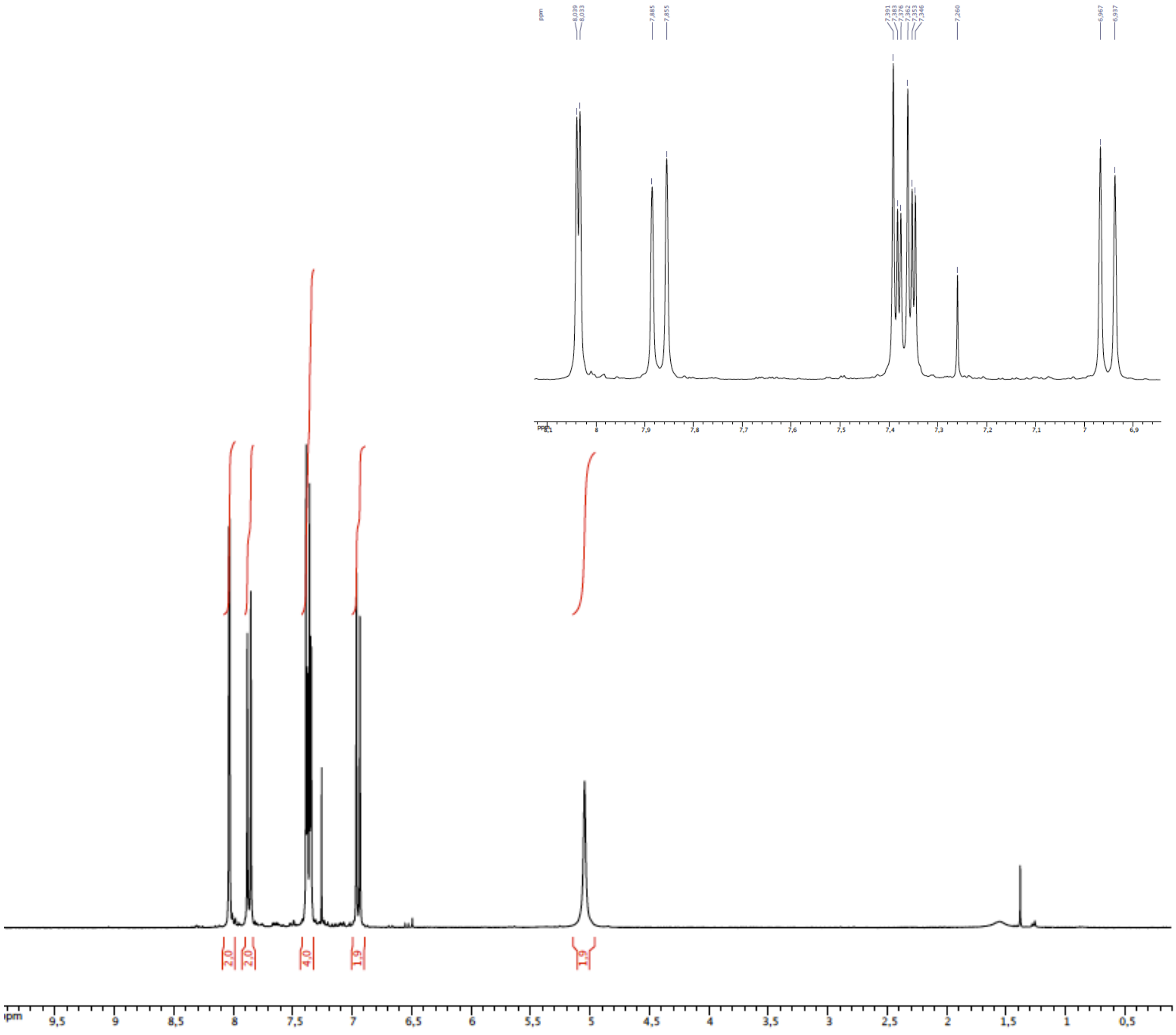

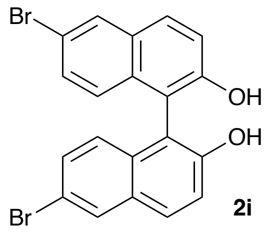

2i

^{1}H-NMR in $CDCl_3$ – list of peaks

Label	Intensity	Frequency(point)	Frequency(ppm)	Frequency(Hz)
1	254180272	26722	8,039	2413,305
2	259524512	26743	8,033	2411,322
3	186421456	27213	7,885	2366,942
4	213030320	27307	7,855	2358,066
5	305851296	28782	7,391	2218,79
6	165339216	28810	7,383	2216,146
7	161255536	28832	7,376	2214,069
8	281273920	28877	7,362	2209,819
9	184620688	28905	7,353	2207,176
10	177870176	28926	7,346	2205,193
11	101360744	29200	7,26	2179,32
12	224496496	30131	6,967	2091,411
13	197355184	30226	6,937	2082,441
14	92200480	36230	5,049	1515,515

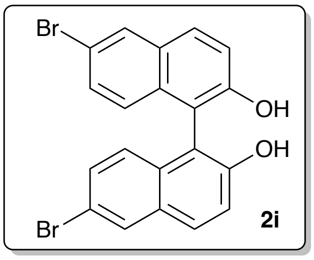

^{13}C NMR in $CDCl_3$

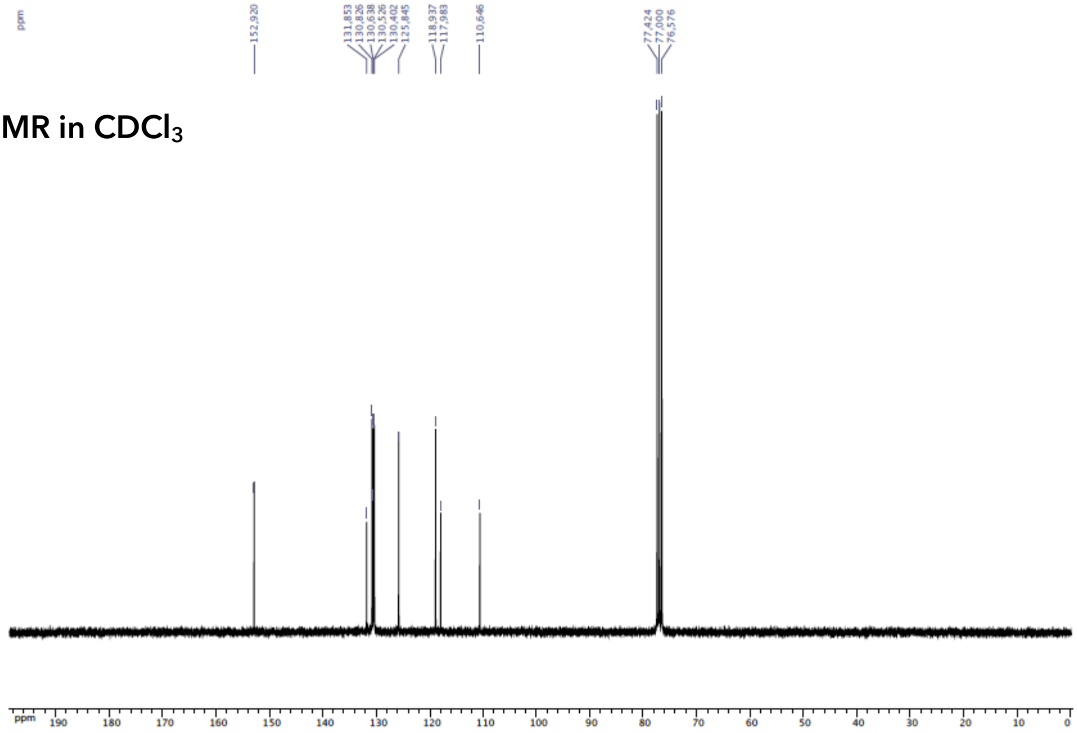

DEPT135 in $CDCl_3$

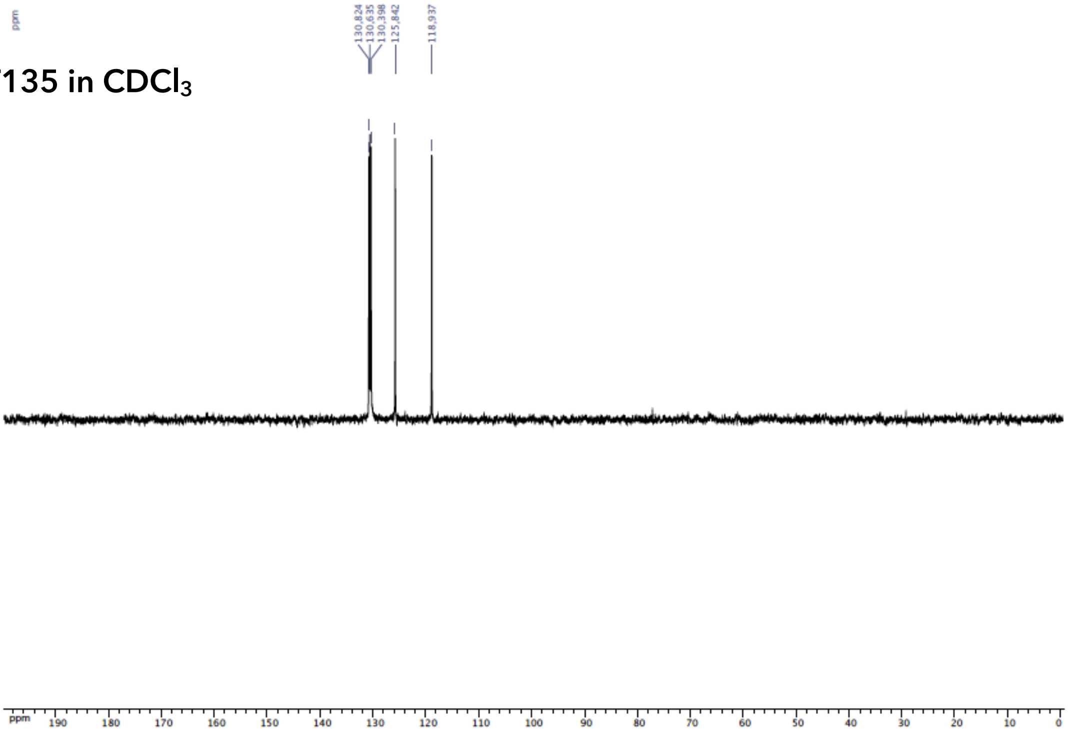

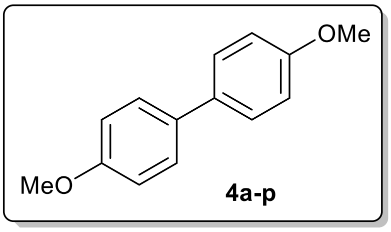

^{1}H-NMR in $CDCl_3$

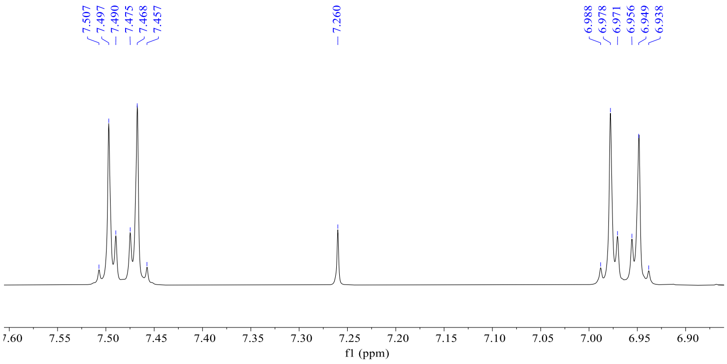

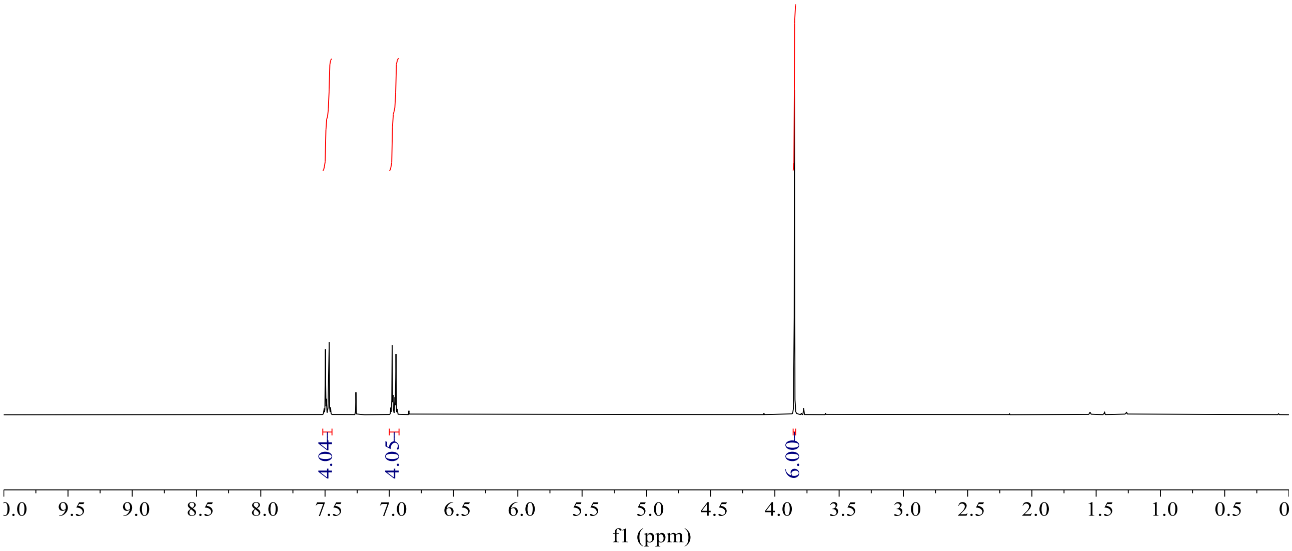

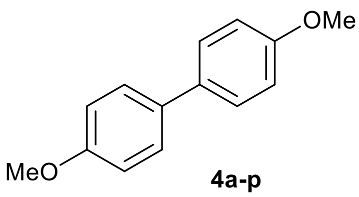

4a-p

^{1}H-NMR in $CDCl_3$ – list of peaks

Label	Intensity	Frequency (ppm)	Frequency (Hz)
1	225	7,507	2253.121
2	2798	7,497	2250.062
3	766	7,490	2247.883
4	807	7,475	2243.419
5	2963	7,468	2241.246
6	262	7,457	2238.214
7	938	7.260	2178.932
8	256	6,988	2097.293
9	2902	6,978	2094.254
10	743	6,971	2092.057
11	702	6,956	2087.583
12	2449	6,949	2085.476
13	206	6.938	2082.366
14	13412	3.848	1154.849

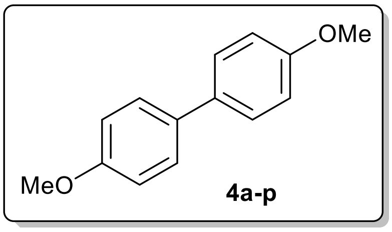
OMe
MeO
4a-p

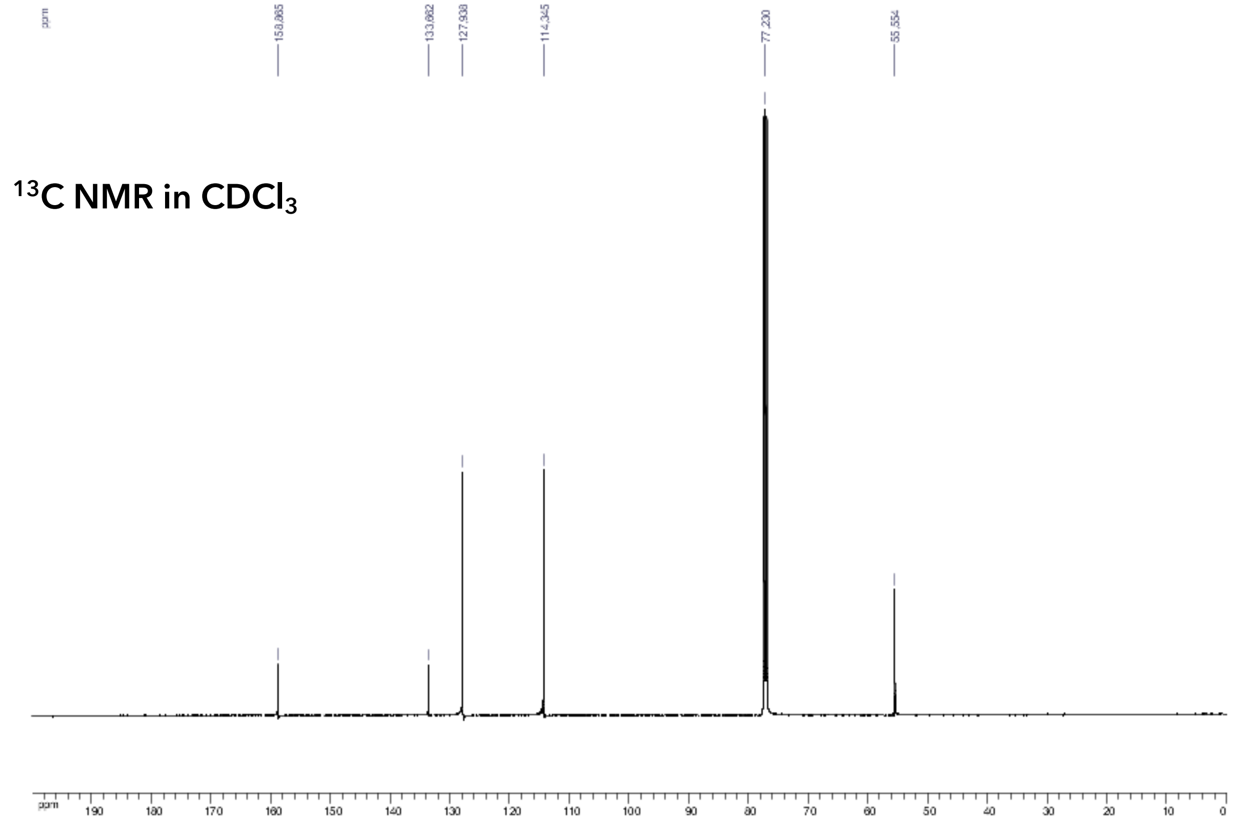
ppm
158.865
133.662
127.938
114.345
77.230
55.554
¹³C NMR in CDCl₃
ppm
190
180
170
160
150
140
130
120
110
100
90
80
70
60
50
40
30
20
10
0

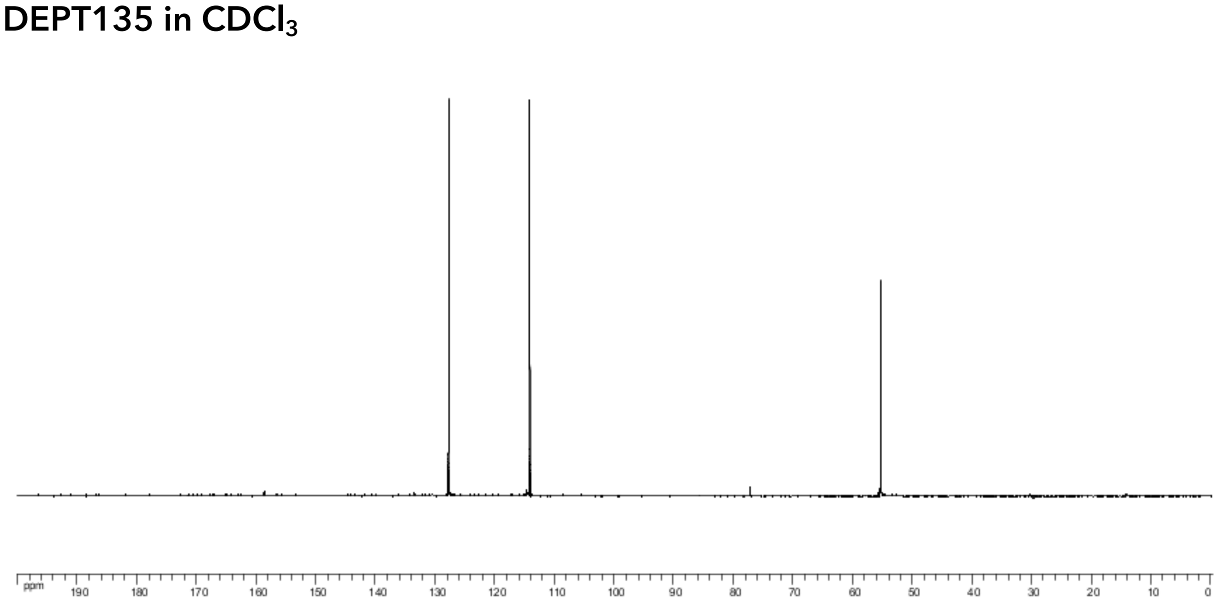
DEPT135 in CDCl₃
ppm
190
180
170
160
150
140
130
120
110
100
90
80
70
60
50
40
30
20
10
0

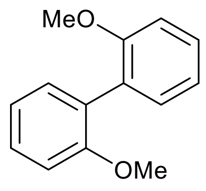

4a-o

^{1}H-NMR in $CDCl_3$

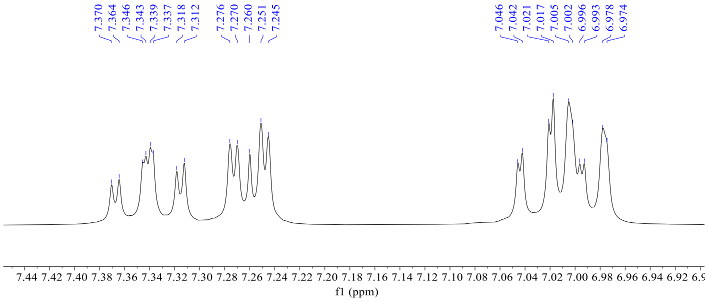

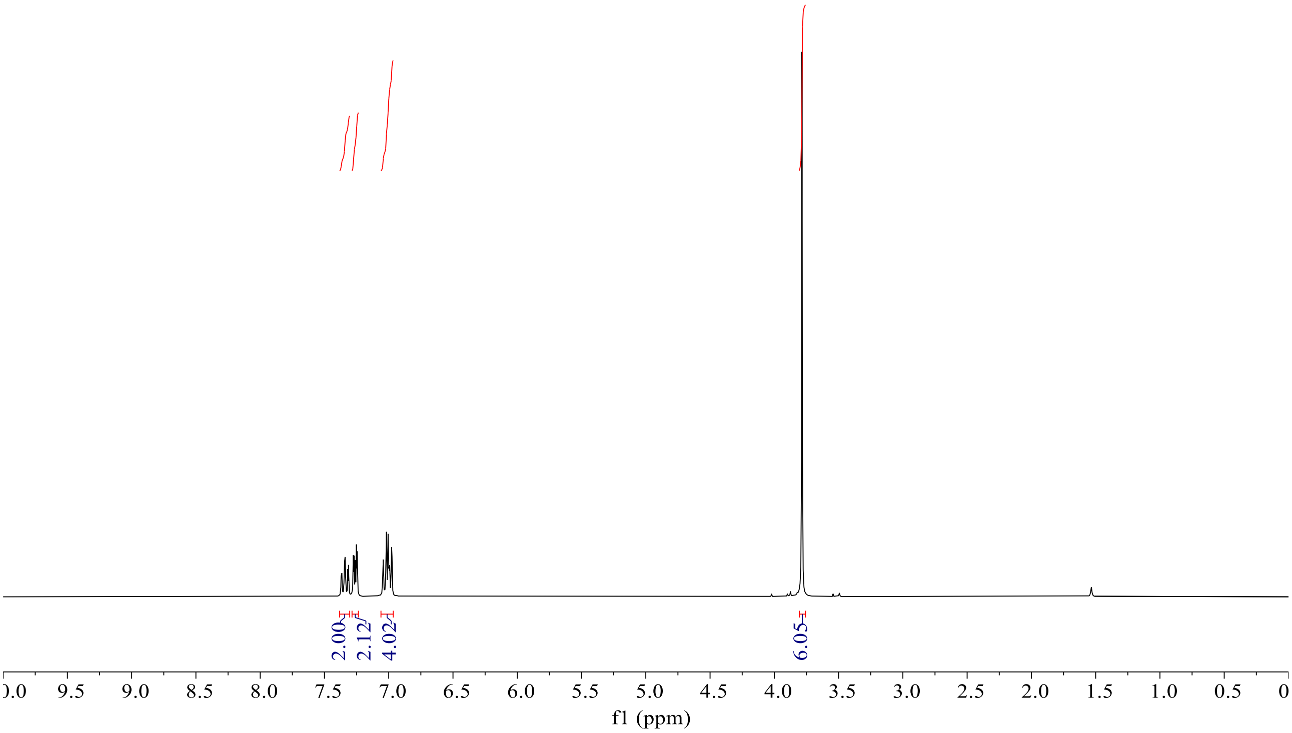

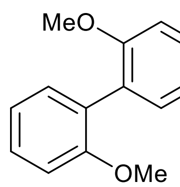

4a-o

^{1}H-NMR in $CDCl_3$ – list of peaks

Label	Intensity	Frequency(ppm)	Frequency(Hz)
1	297	7.370	2212.108
2	338	7.364	2210.281
3	335	7.346	2204.713
4	369	7.343	2203.856
5	370	7.339	2202.801
6	423	7.337	2202.076
7	400	7.318	2196.465
8	470	7.312	2194.662
9	600	7.276	2183.728
10	572	7.270	2181.919
11	503	7.260	2178.958
12	785	7.251	2176.264
13	640	7.245	2174.448
14	388	7.046	2114.713
15	516	7.042	2113.553
16	671	7.021	2107.249
17	880	7.017	2106.144
18	796	7.005	2102.540
19	494	7.002	2101.469
20	252	6.996	2099.784
21	375	6.993	2098.698
22	650	6.978	2094.374
23	498	6.974	2093.244
24	8988	3.785	1135.884

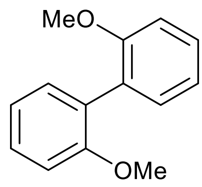

4a-o

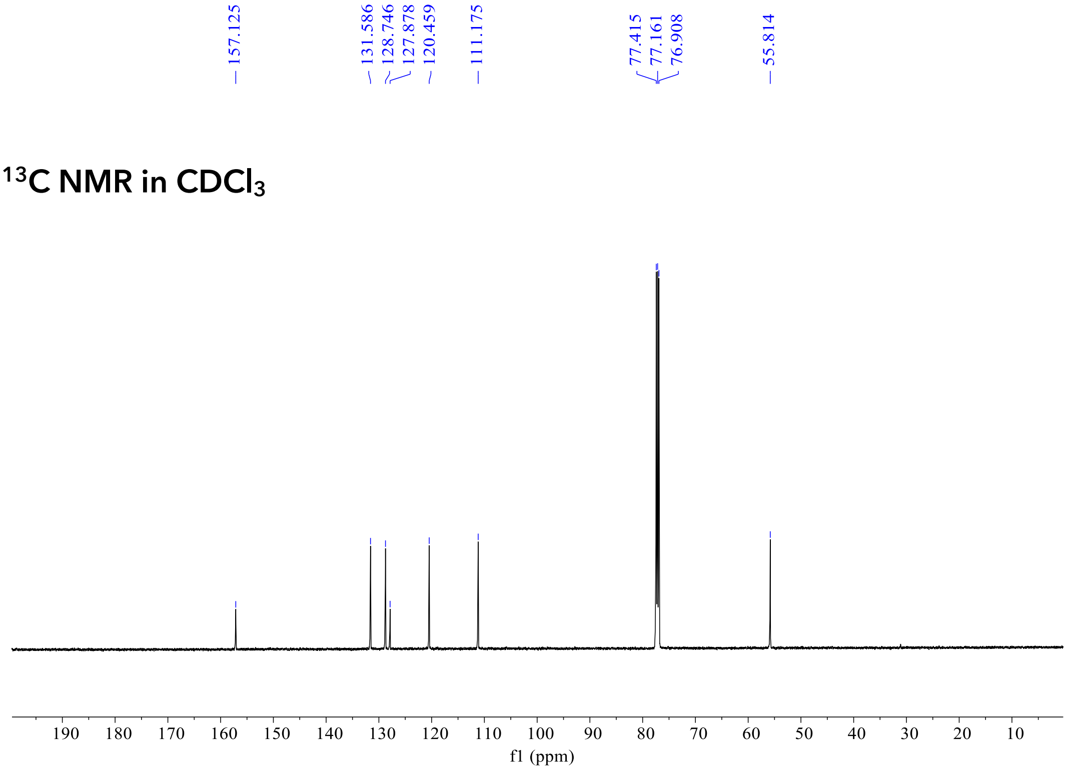

^{13}C NMR in $CDCl_3$

DEPT135 in $CDCl_3$

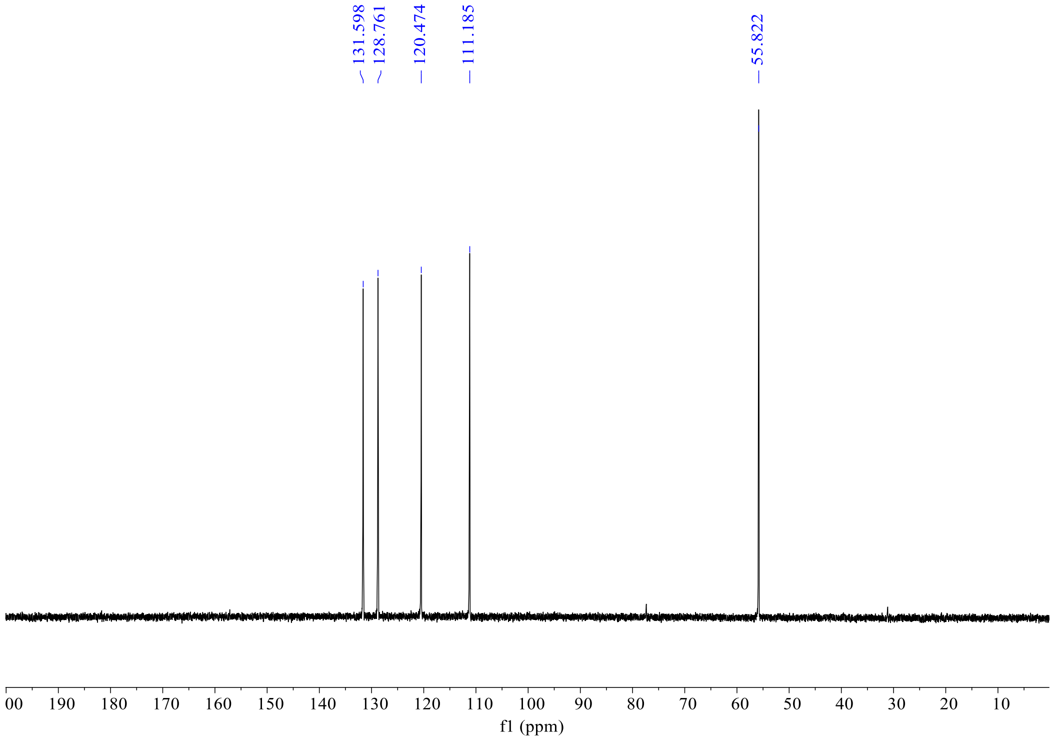

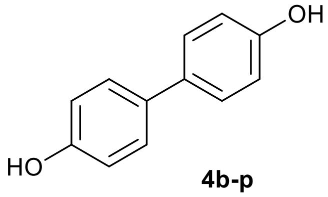

4b-p

^{1}H-NMR in DMSO-d$_6$

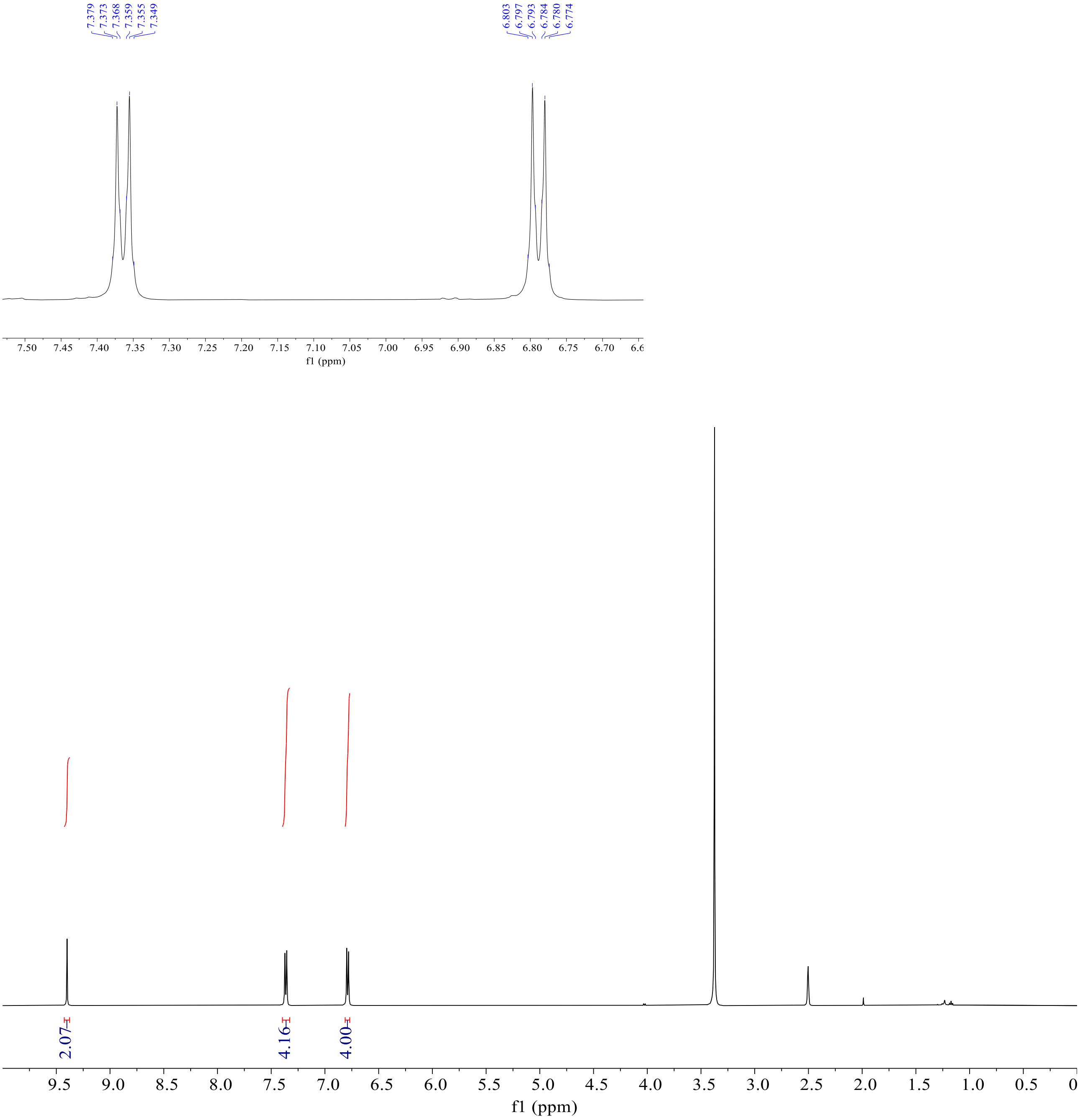

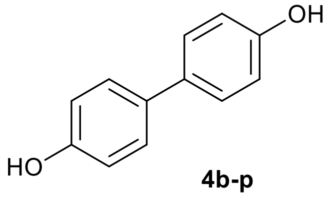

4b-p

^{1}H-NMR in DMSO-d_6– list of peaks

Label	Intensity	Frequency(ppm)	Frequency(Hz)
1	10735	9.396	4699.445
2	751	7.376	3688.856
3	7665	7.370	3685.804
4	2562	7.365	3683.668
5	3383	7.357	3679.243
6	8161	7.352	3677.107
7	717	7.346	3674.055
8	818	6.800	3401.075
9	8454	6.794	3398.023
10	2755	6.790	3395.887
11	3261	6.781	3391.462
12	7985	6.777	3389.326
13	652	6.771	3386.274
14	5186	2.500	1250.398

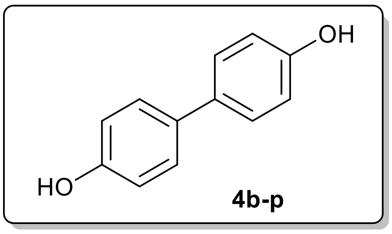

4b-p

^{13}C NMR in DMSO-d$_6$

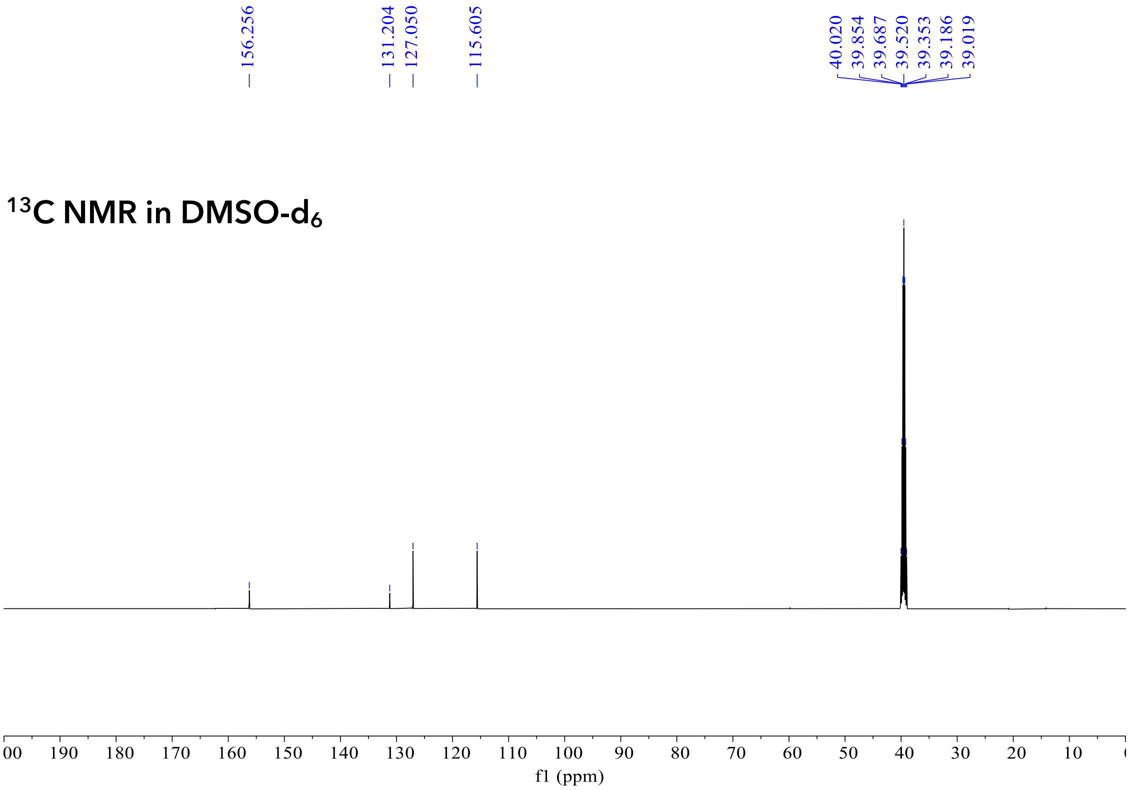

DEPT135 in DMSO-d$_6$

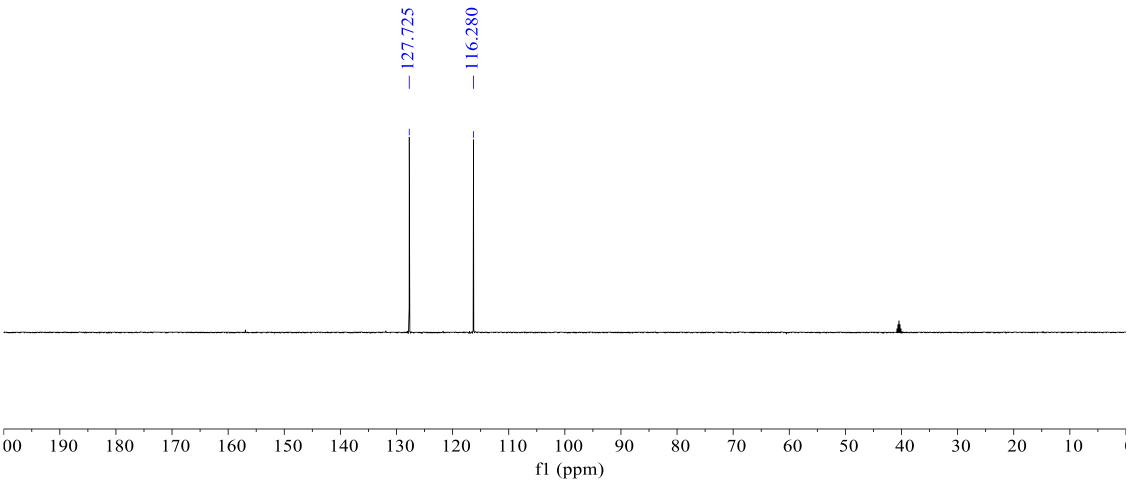

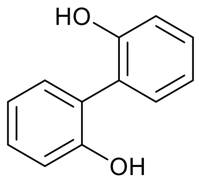

4b-o

^{1}H-NMR in CDCl$_3$

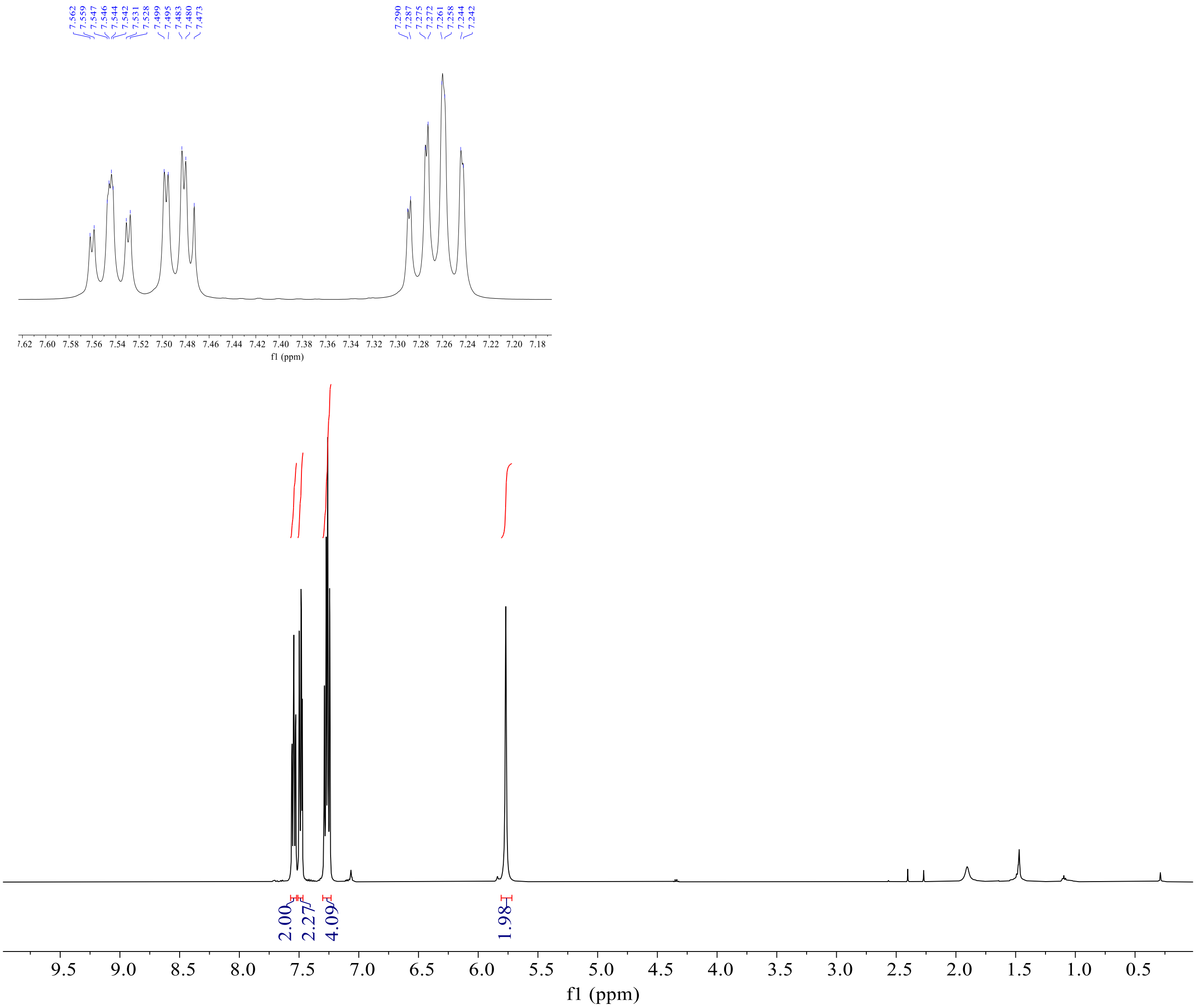

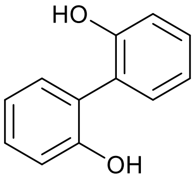

4b-o

^{1}H-NMR in $CDCl_3$ – list of peaks

Label	Intensity	Frequency(ppm)	Frequency(Hz)
1	1905	7.562	3782.013
2	2219	7.559	3780.239
3	2255	7.547	3774.667
4	1811	7.546	3773.870
5	2815	7.544	3772.850
6	2233	7.542	3772.104
7	2332	7.531	3766.454
8	2611	7.528	3764.776
9	4003	7.499	3750.267
10	3985	7.495	3748.499
11	4895	7.483	3742.650
12	4156	7.480	3740.972
13	3208	7.473	3737.375
14	2105	7.290	3645.855
15	3146	7.287	3644.637
16	3779	7.275	3638.398
17	5435	7.272	3637.136
18	6378	7.261	3631.252
19	5495	7.258	3629.981
20	4445	7.244	3623.174
21	3641	7.242	3622.001
22	5229	5.768	2884.801

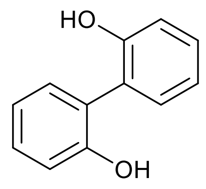

4b-o

^{13}C NMR in $CDCl_3$

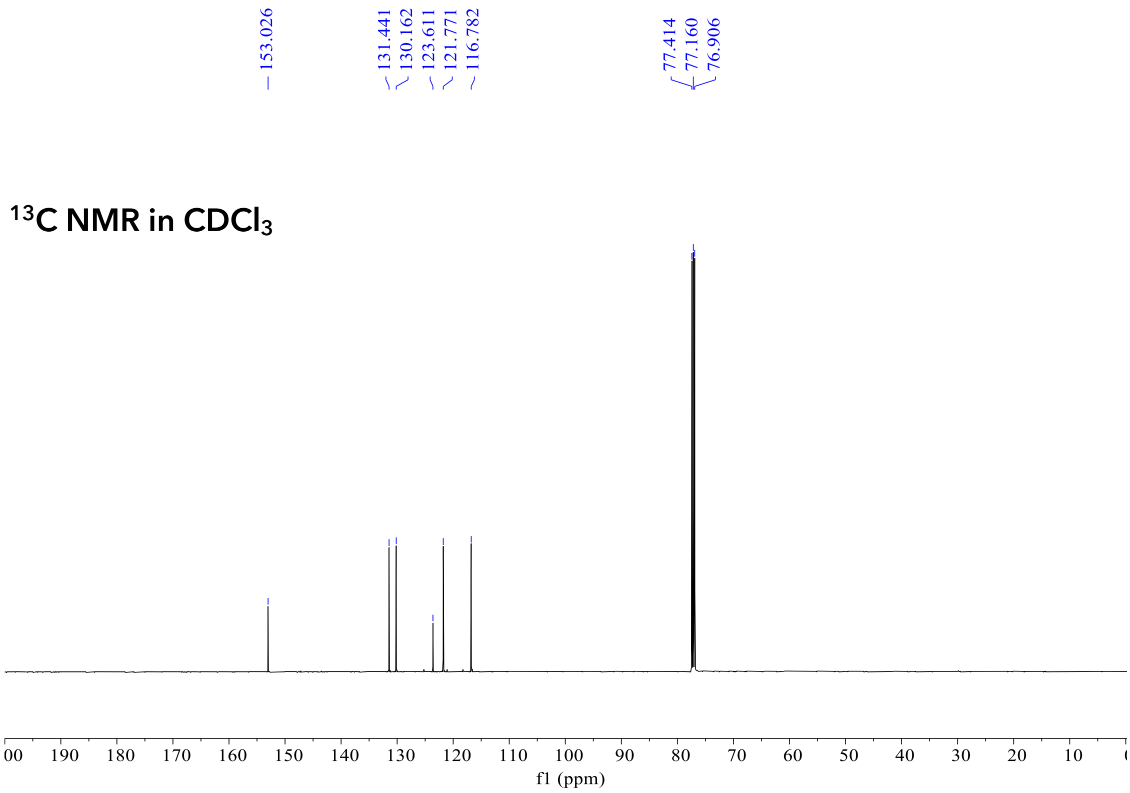

DEPT135 in $CDCl_3$

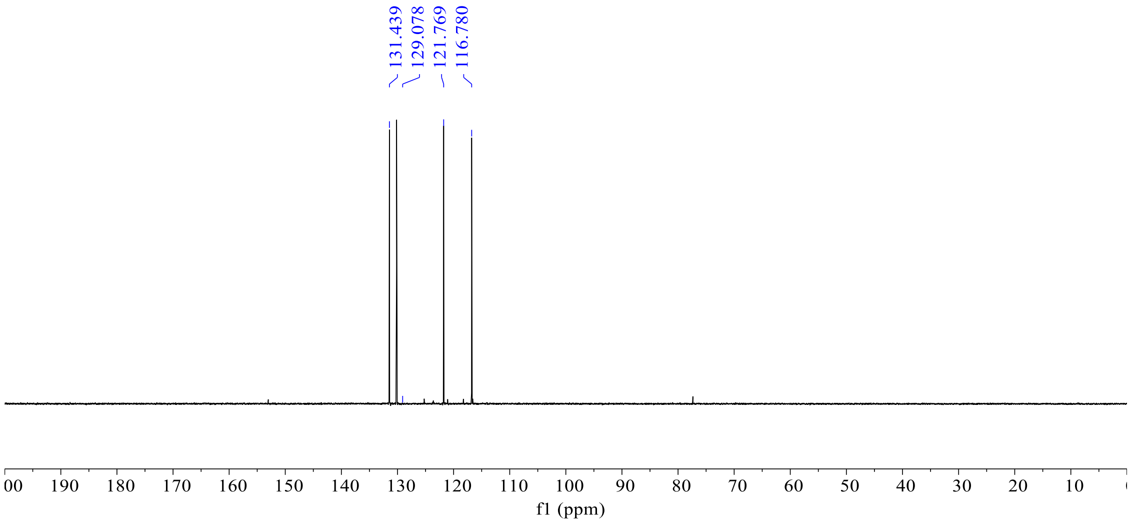

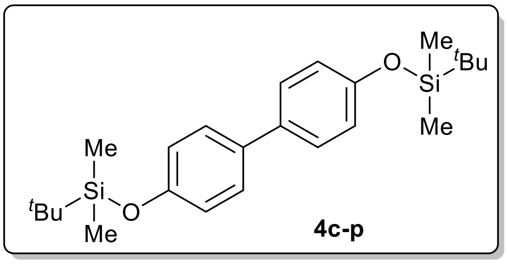

^{1}H-NMR in $CDCl_3$

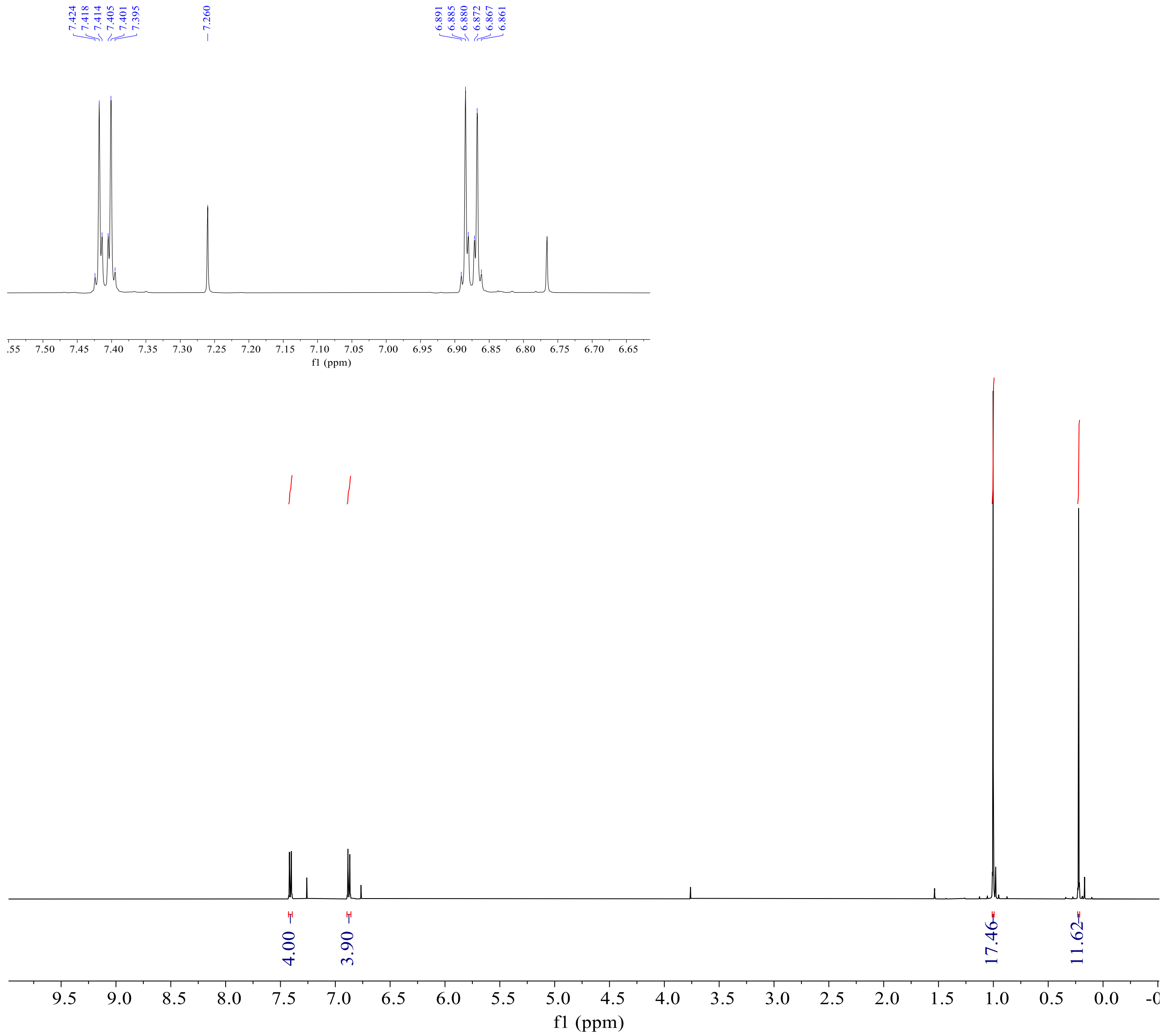

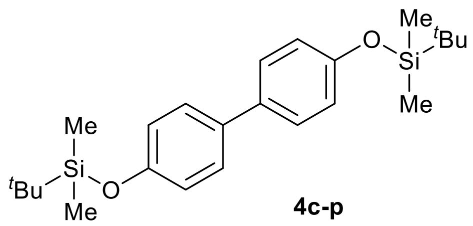

4c-p

^{1}H-NMR in $CDCl_3$ – list of peaks

Label	Intensity	Frequency(ppm)	Frequency(ppm)
1	124359	7.424	3713.066
2	2178390	7.418	3709.973
3	529825	7.414	3707.895
4	552534	7.405	3703.494
5	2276106	7.401	3701.424
6	197767	7.395	3698.419
7	1007399	7.260	3630.953
8	146030	6.891	3446.239
9	2348760	6.885	3443.153
10	541732	6.880	3441.095
11	505033	6.872	3436.658
12	2135250	6.867	3434.642
13	165746	6.861	3431.582
14	23574805	1.002	501.183
15	17801655	0.222	111.088

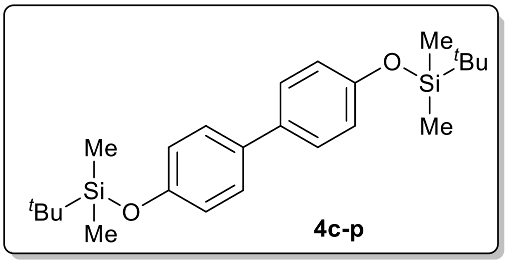

4c-p

^{13}C NMR in $CDCl_3$

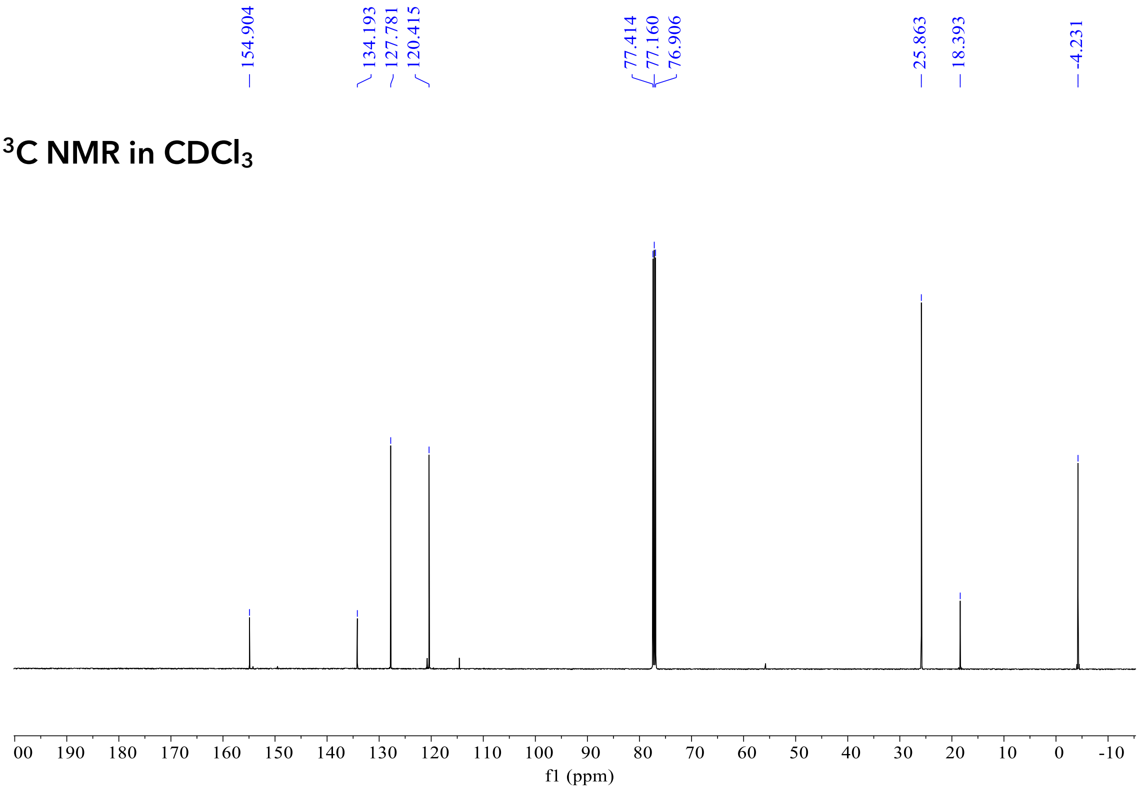

DEPT135 in $CDCl_3$

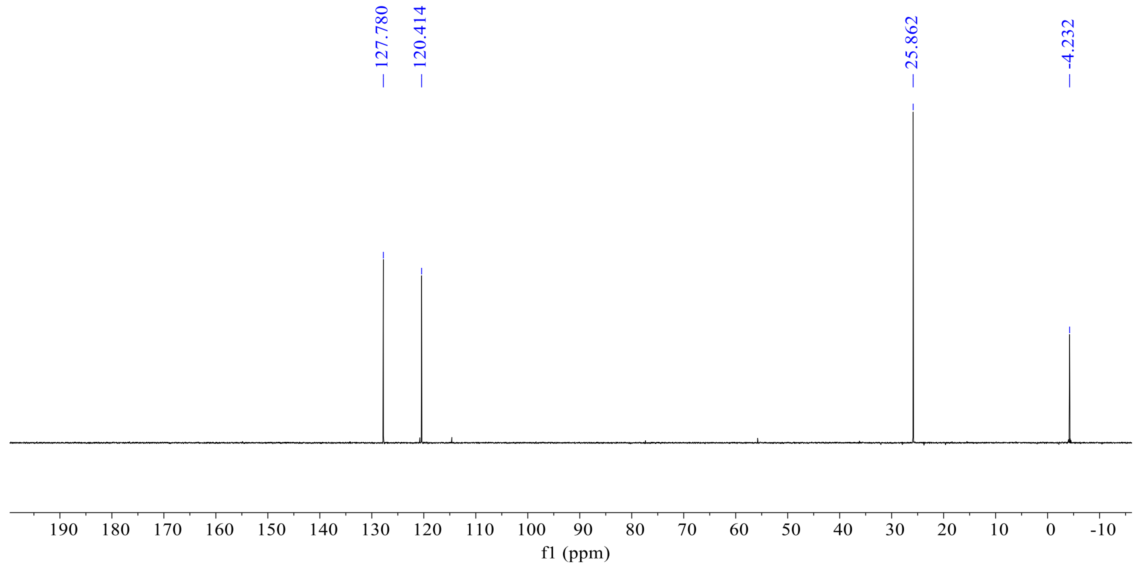

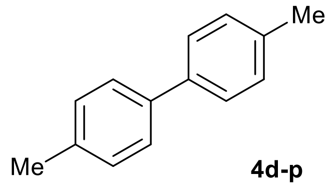

4d-p

^{1}H-NMR in $CDCl_3$

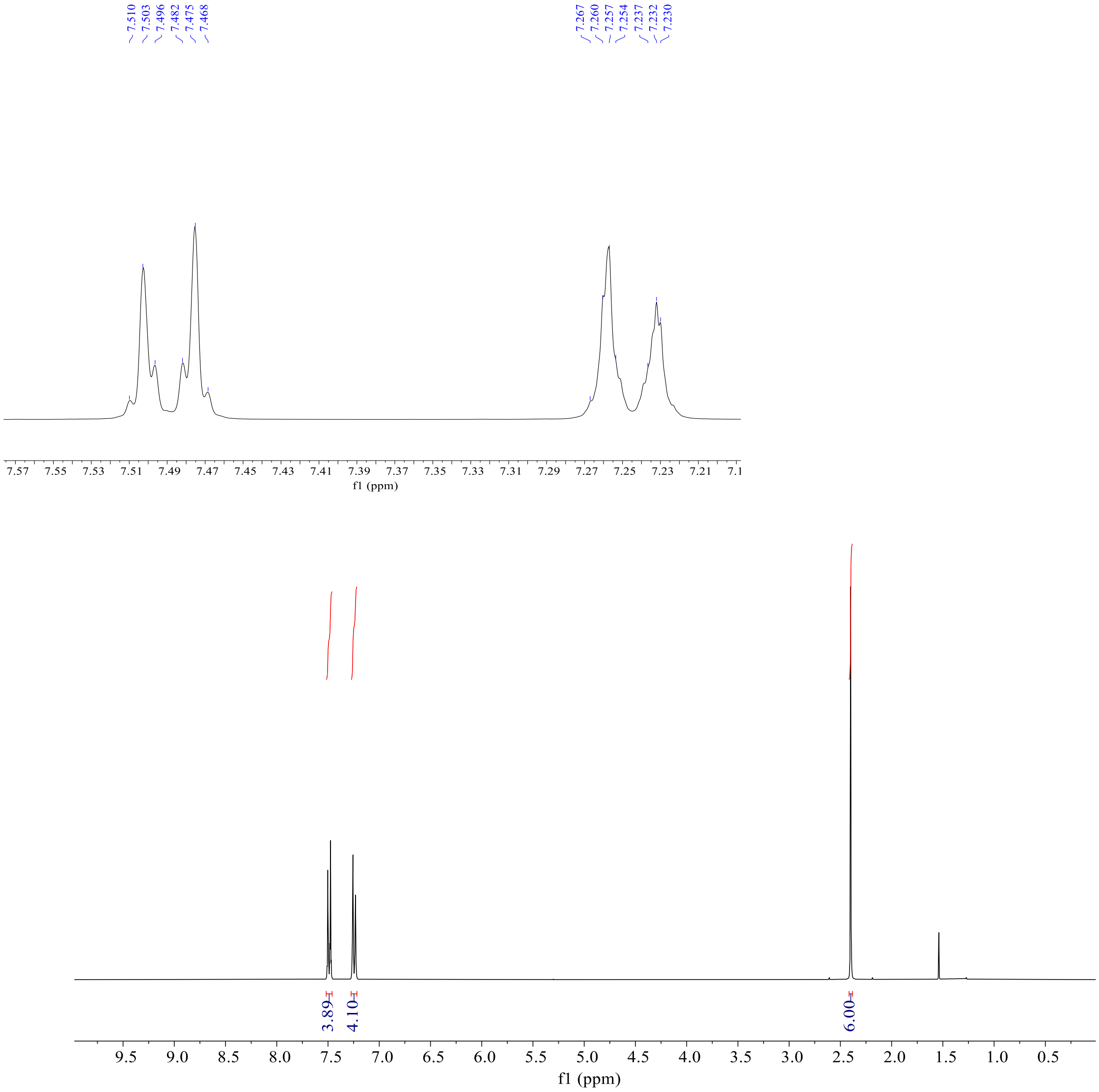

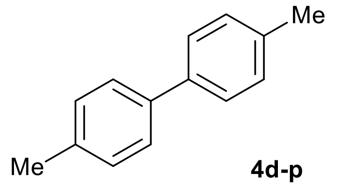

4d-p

^{1}H-NMR in $CDCl_3$ – list of peaks

Label	Intensity	Frequency(ppm)	Frequency(Hz)
1	541	7.510	2253.942
2	5373	7.503	2251.823
3	1825	7.496	2249.888
4	1902	7.482	2245.557
5	6888	7.475	2243.530
6	851	7.468	2241.503
7	316	7.267	2181.058
8	2789	7.261	2179.100
9	6006	7.257	2178.017
10	1519	7.254	2177.003
11	1542	7.237	2171.936
12	4084	7.232	2170.553
13	3332	7.230	2169.908
14	19961	2.400	720.231

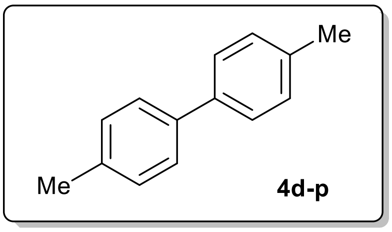

^{13}C NMR in $CDCl_3$

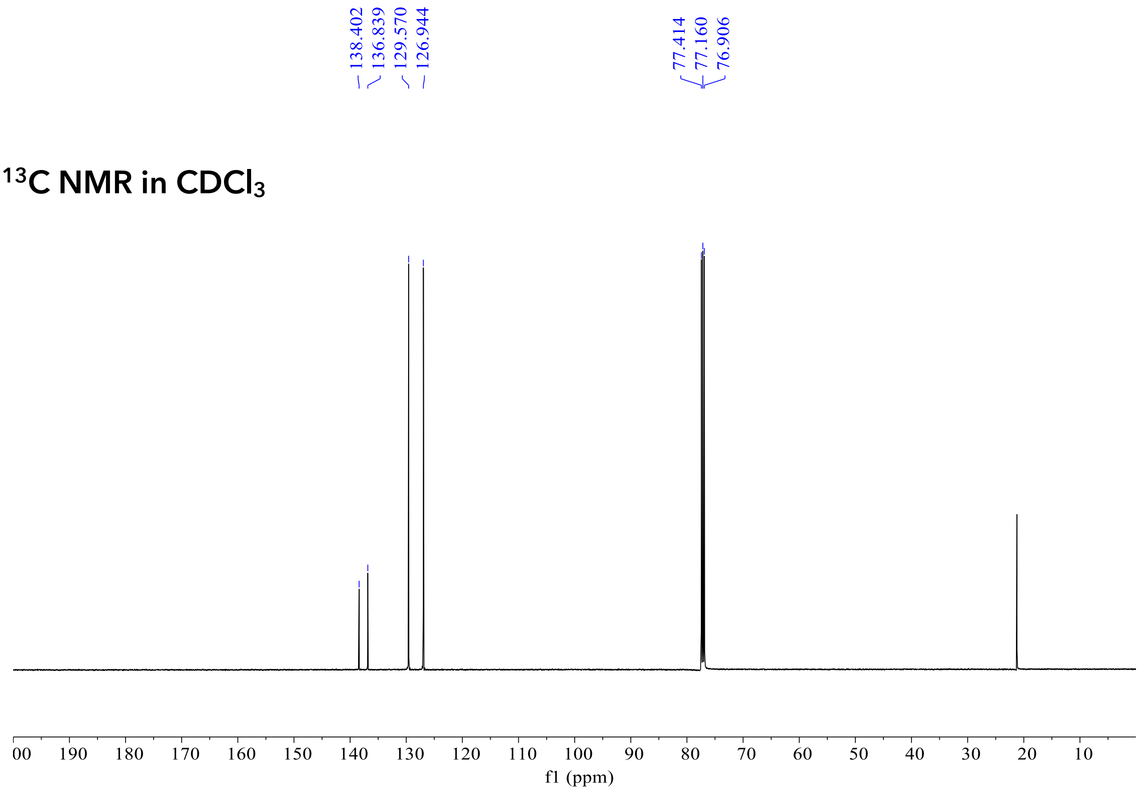

DEPT135 in $CDCl_3$

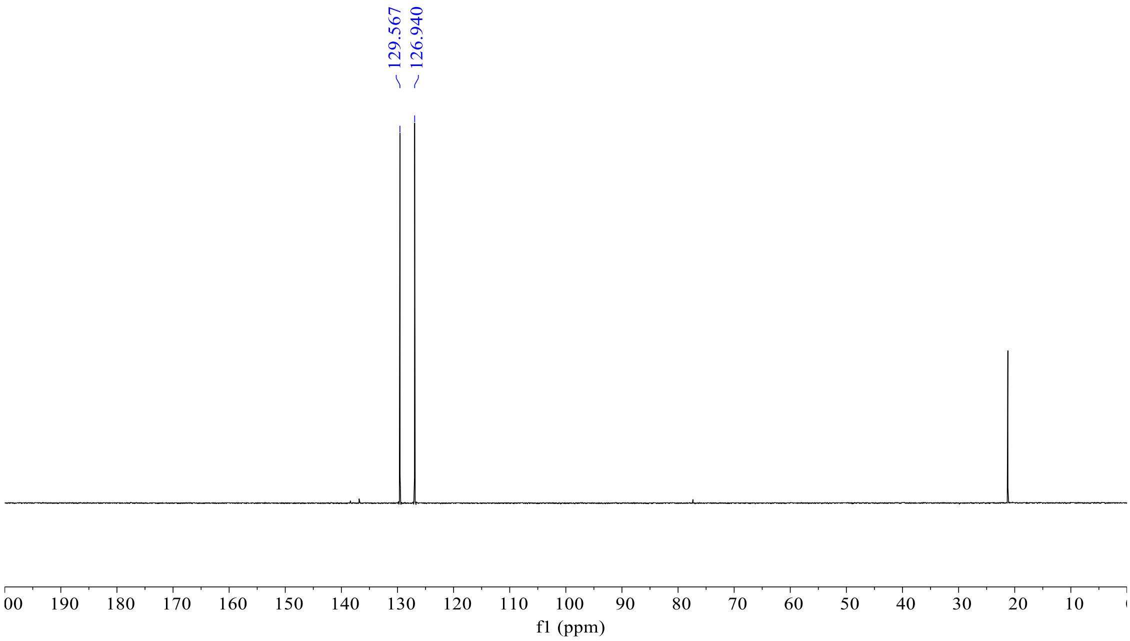

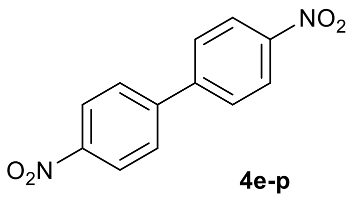

4e-p

^{1}H-NMR in $CDCl_3$

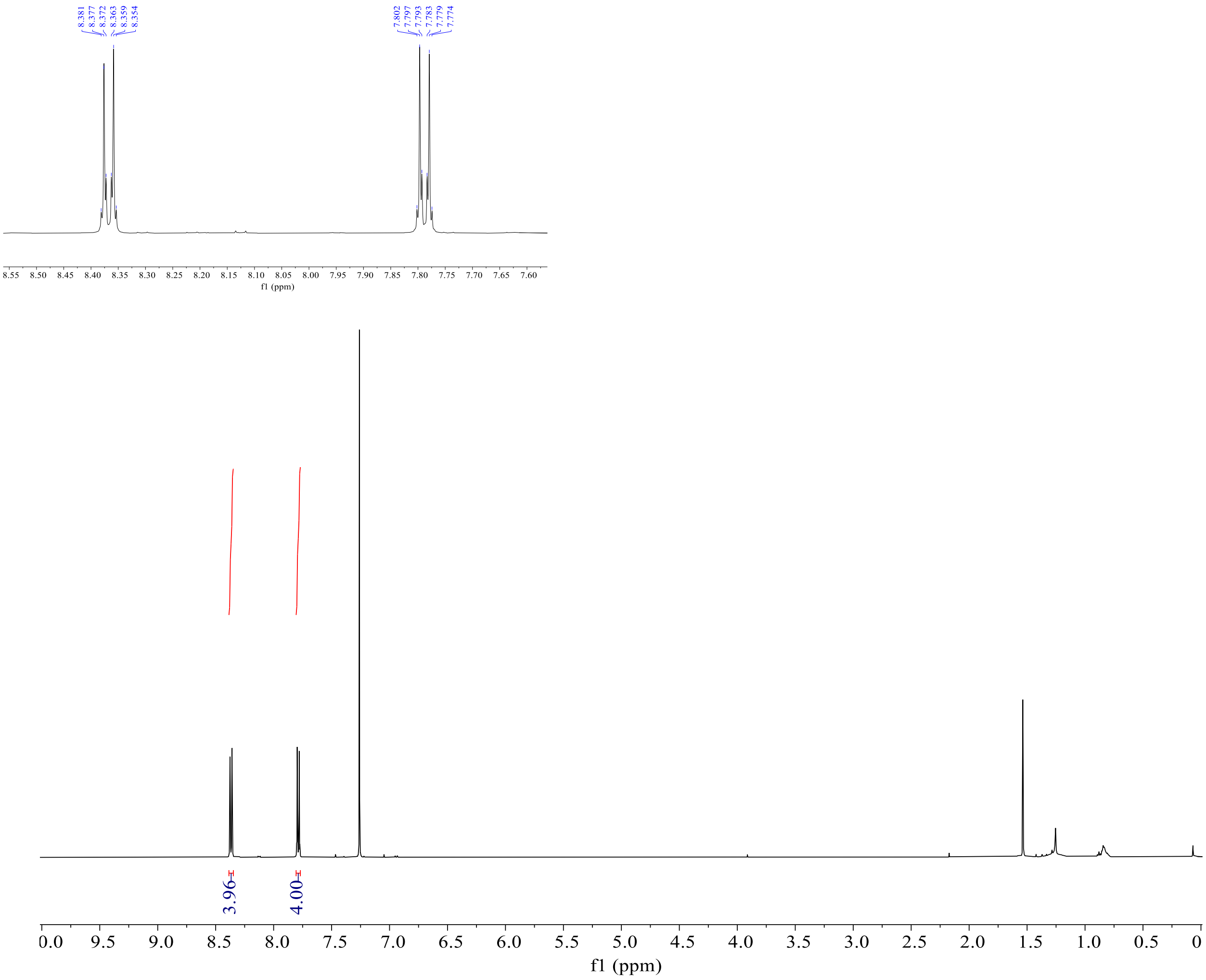

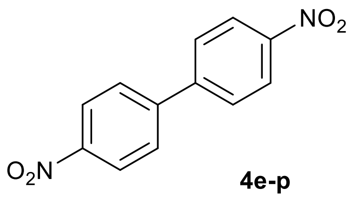

4e-p

^{1}H-NMR in $CDCl_3$ – list of peaks

Label	Intensity	Frequency(ppm)	Frequency(Hz)
1	23	8.381	4192.704
2	289	8.377	4190.263
3	80	8.372	4188.160
4	89	8.363	4183.363
5	327	8.359	4181.259
6	30	8.354	4178.819
7	29	7.802	3902.815
8	329	7.797	3900.374
9	95	7.793	3898.271
10	83	7.783	3893.558
11	317	7.779	3891.455
12	28	7.774	3889.014
13	1616	7.260	3631.735

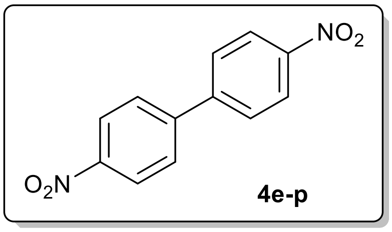
NO2
O2N

4e-p

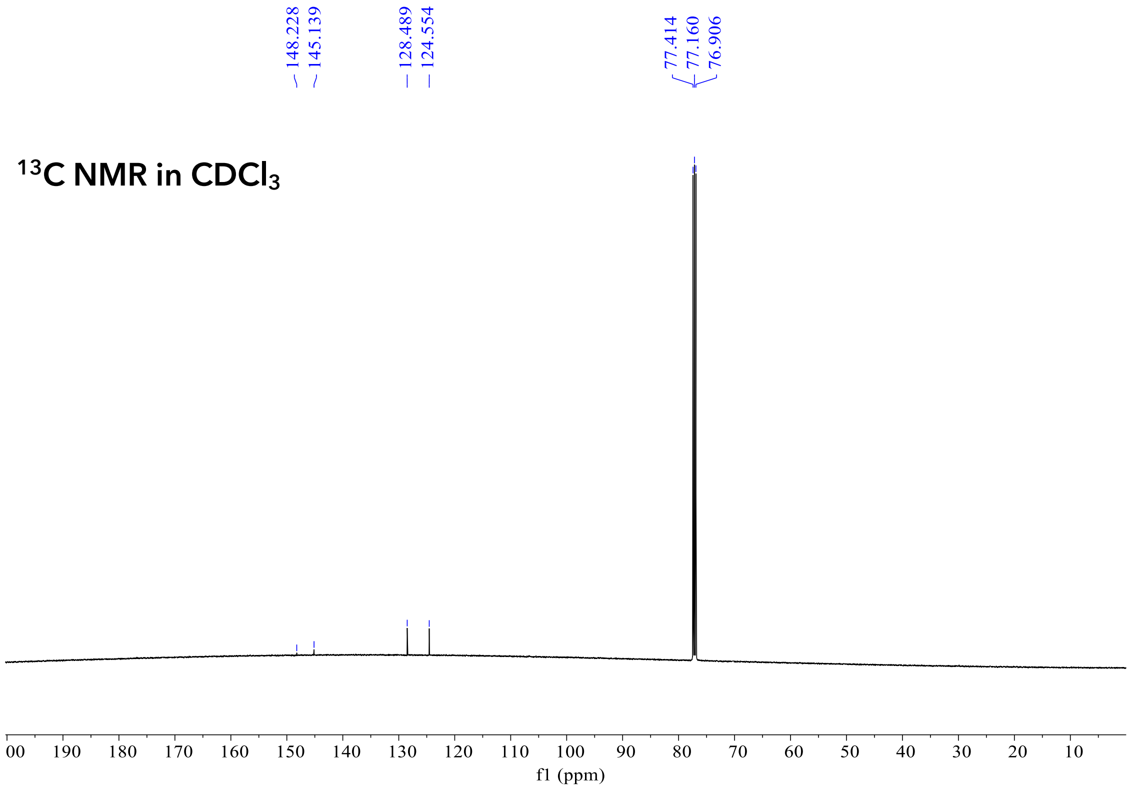
13C NMR in CDCl3
148.228
145.139
128.489
124.554
77.414
77.160
76.906
f1 (ppm)

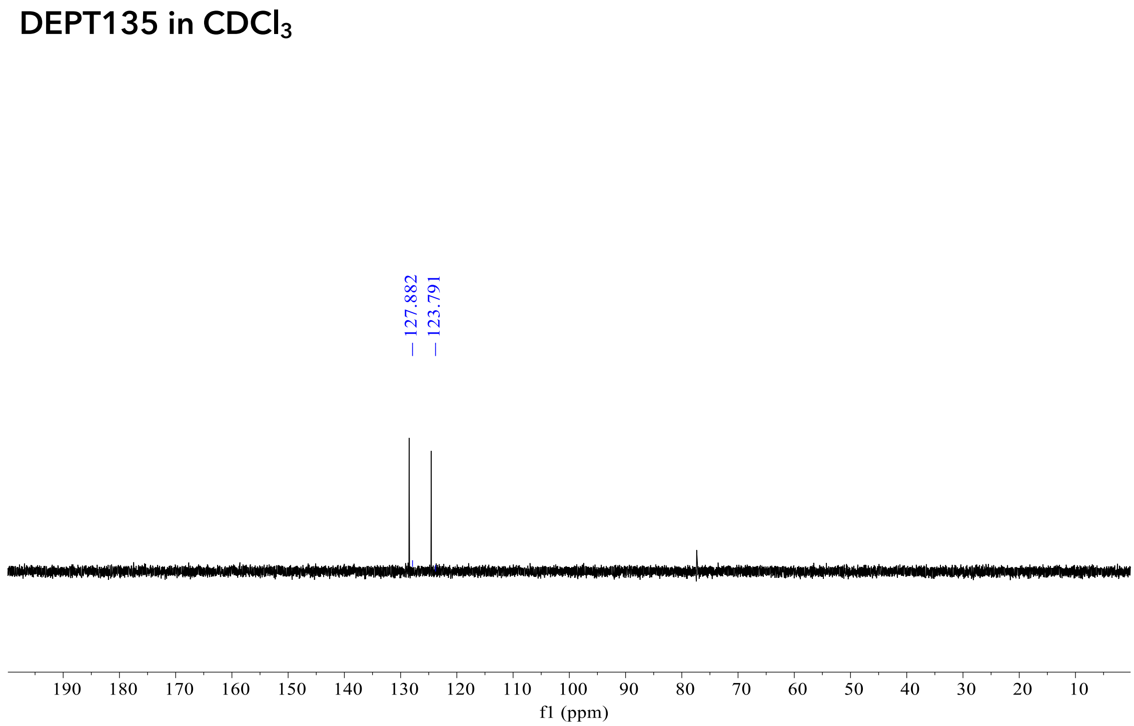
DEPT135 in CDCl3
127.882
123.791
f1 (ppm)

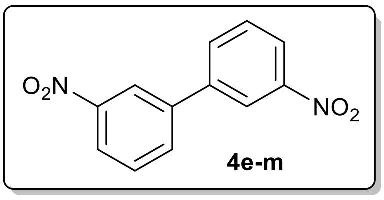

^{1}H-NMR in $CDCl_3$

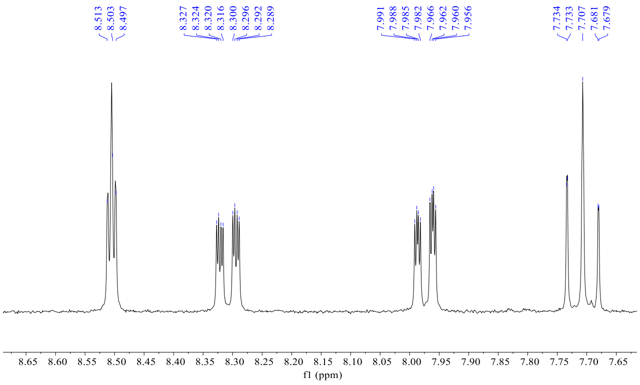

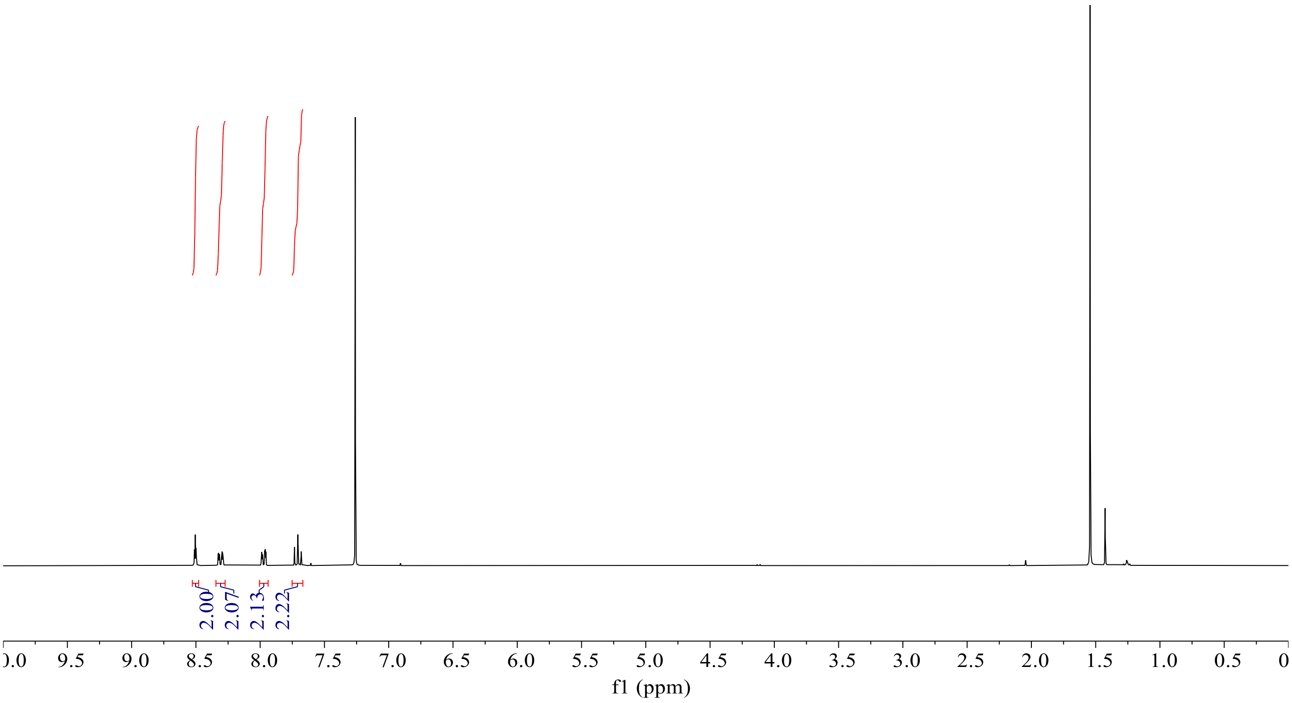

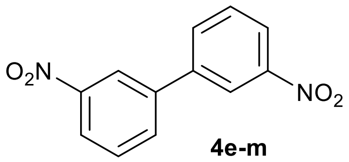

4e-m

^{1}H-NMR in $CDCl_3$ – list of peaks

Label	Intensity	Frequency(ppm)	Frequency(Hz)
1	137	8.513	2554.877
2	234	8.503	2552.126
3	153	8.497	2550.292
4	138	8.327	2499.215
5	148	8.324	2498.207
6	132	8.320	2497.015
7	131	8.316	2495.914
8	150	8.300	2491.962
9	162	8.296	2490.954
10	152	8.292	2488.762
11	114	8.289	2487.753
12	143	7.991	2398.438
13	163	7.988	2397.429
14	154	7.985	2396.604
15	125	7.982	2395.595
16	153	7.966	2390.735
17	192	7.962	2389.635
18	192	7.960	2388.901
19	163	7.956	2387.801
20	206	7.734	2321.318
21	217	7.733	2320.768
22	380	7.707	2313.066
23	171	7.681	2305.271
24	169	7.679	2304.813
25	5763	7.260	2178.923

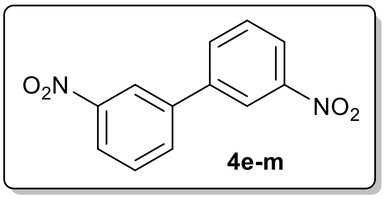

4e-m

^{13}C NMR in CDCl$_3$

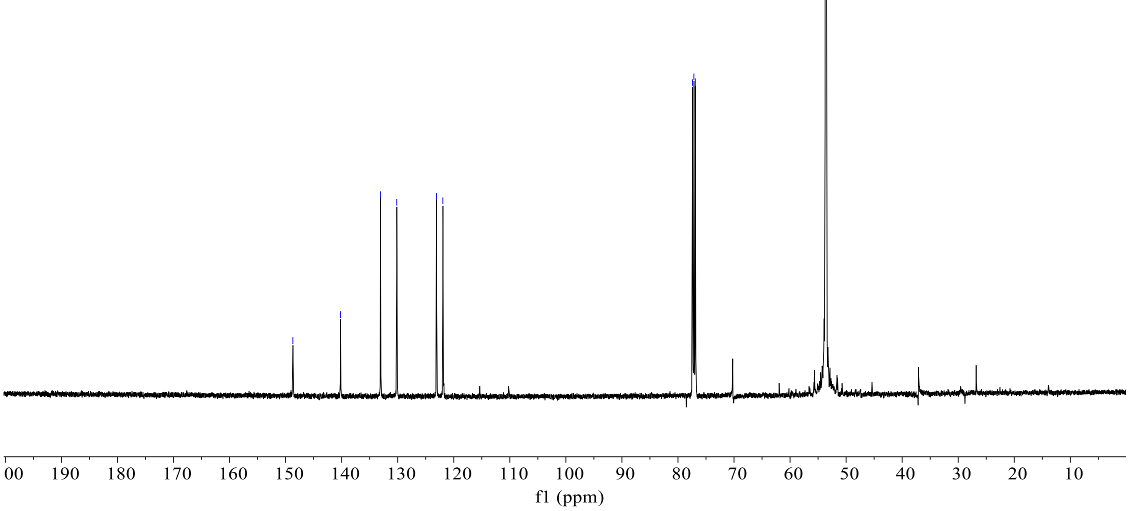

DEPT135 in CDCl$_3$

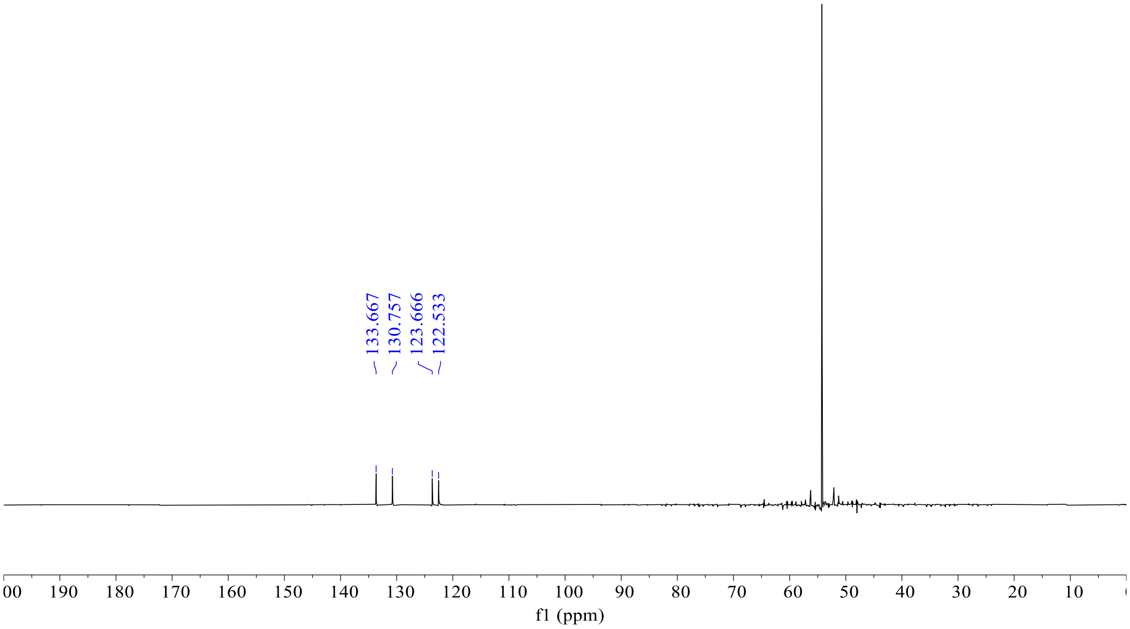

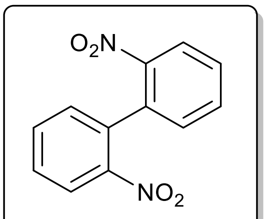

4e-o

^{1}H-NMR in $CDCl_3$

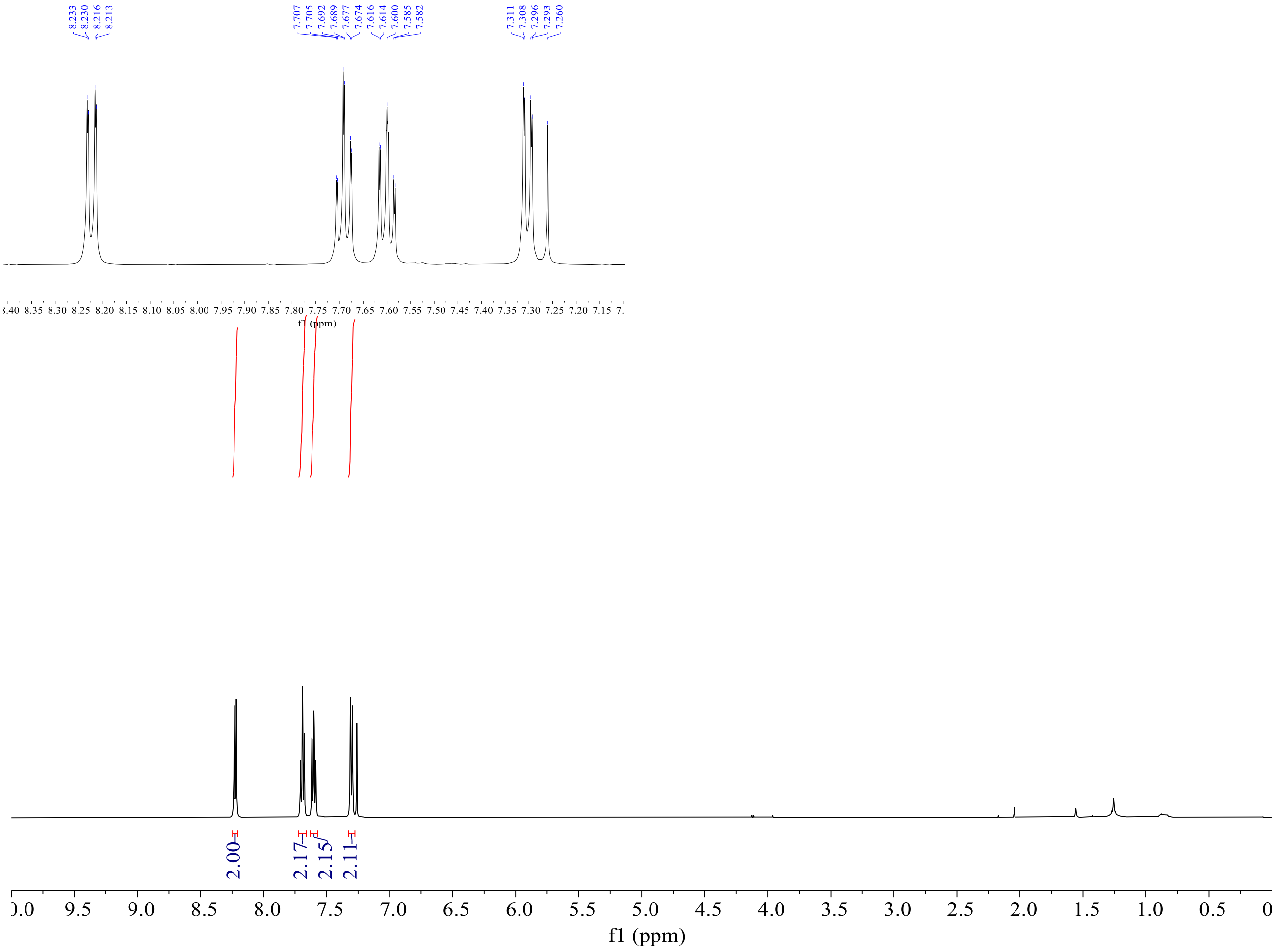

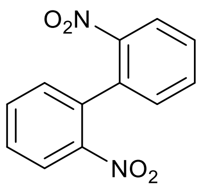

4e-o

^{1}H-NMR in $CDCl_3$ – list of peaks

Label	Intensity	Frequency(ppm)	Frequency(Hz)
1	5721	8.233	4117.407
2	5098	8.230	4116.034
3	6102	8.216	4109.167
4	5337	8.213	4107.794
5	2649	7.707	3854.498
6	2503	7.705	3853.277
7	6252	7.692	3847.021
8	5710	7.689	3845.648
9	4061	7.677	3839.392
10	3581	7.674	3838.171
11	3843	7.616	3809.179
12	3676	7.614	3807.806
13	5024	7.600	3800.940
14	2703	7.585	3793.463
15	2385	7.582	3792.089
16	6106	7.311	3656.591
17	5618	7.296	3648.962
18	4858	7.293	3647.436
19	5036	7.260	3630.957

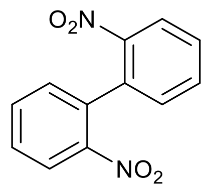

4e-o

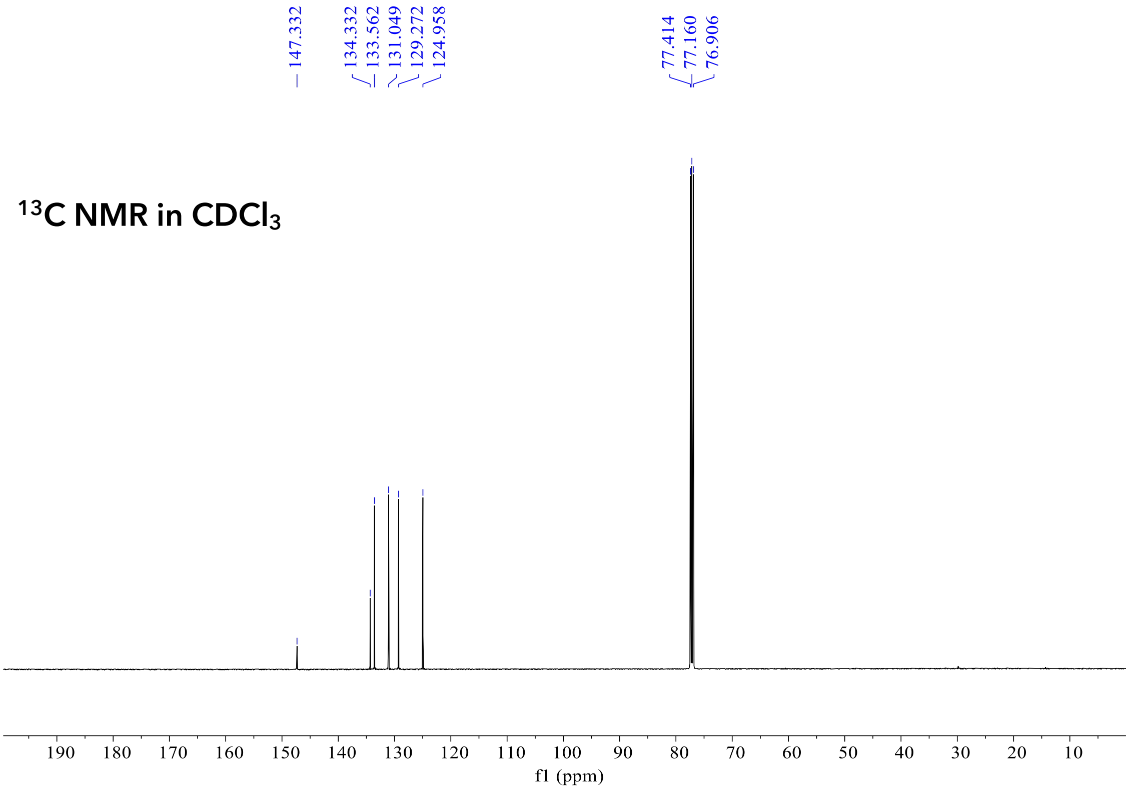

^{13}C NMR in $CDCl_3$

DEPT135 in $CDCl_3$

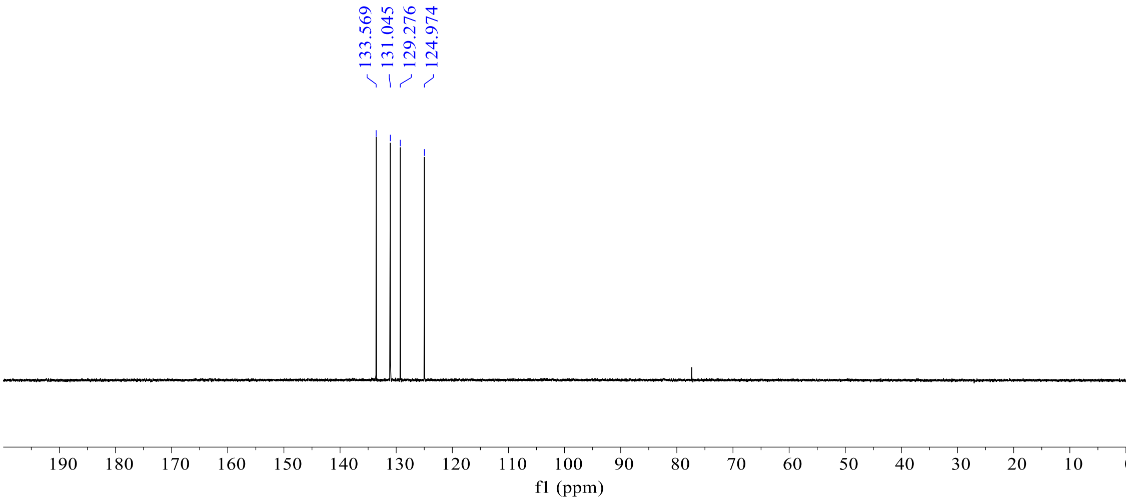

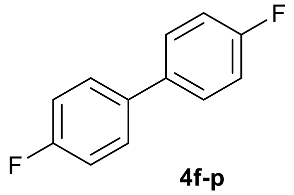

4f-p

^{1}H-NMR in $CDCl_3$

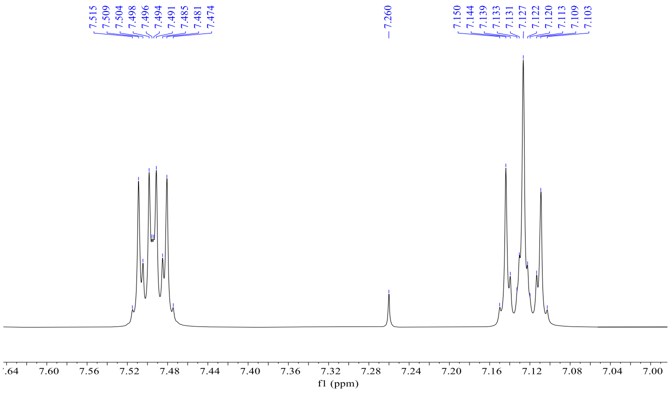

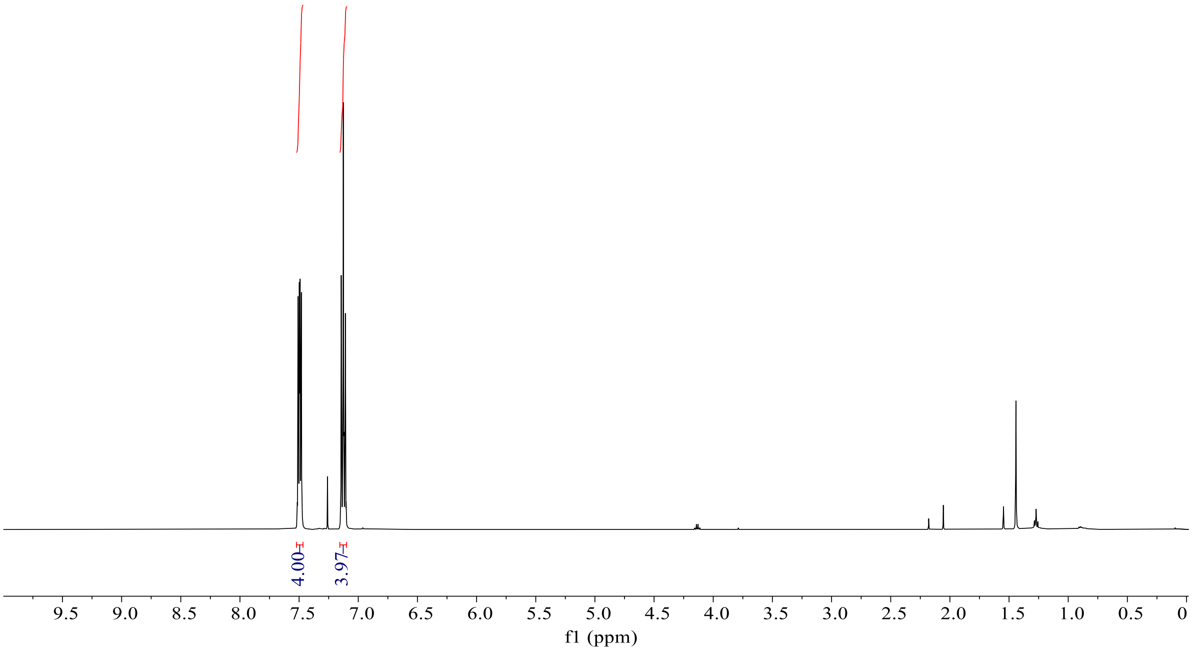

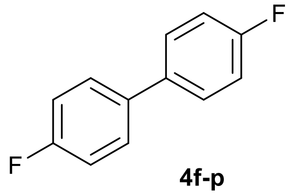

4f-p

^{1}H-NMR in $CDCl_3$ – list of peaks

Label	Intensity	Frequency(ppm)	Frequency(Hz)
1	248	7.515	3759.305
2	3214	7.509	3756.247
3	1073	7.504	3754.045
4	3132	7.498	3750.979
5	1034	7.496	3749.550
6	746	7.494	3748.638
7	3148	7.491	3747.398
8	1187	7.485	3744.294
9	3261	7.481	3742.153
10	284	7.474	3738.980
11	739	7.260	3631.805
12	309	7.150	3576.708
13	3569	7.144	3573.650
14	828	7.139	3571.446
15	305	7.133	3568.130
16	916	7.131	3566.992
17	6078	7.127	3565.003
18	805	7.122	3562.822
19	227	7.120	3561.626
20	914	7.113	3558.351
21	2910	7.109	3556.272
22	266	7.103	3553.078

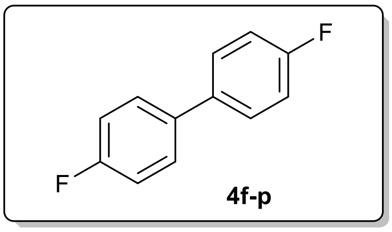

^{13}C NMR in $CDCl_3$

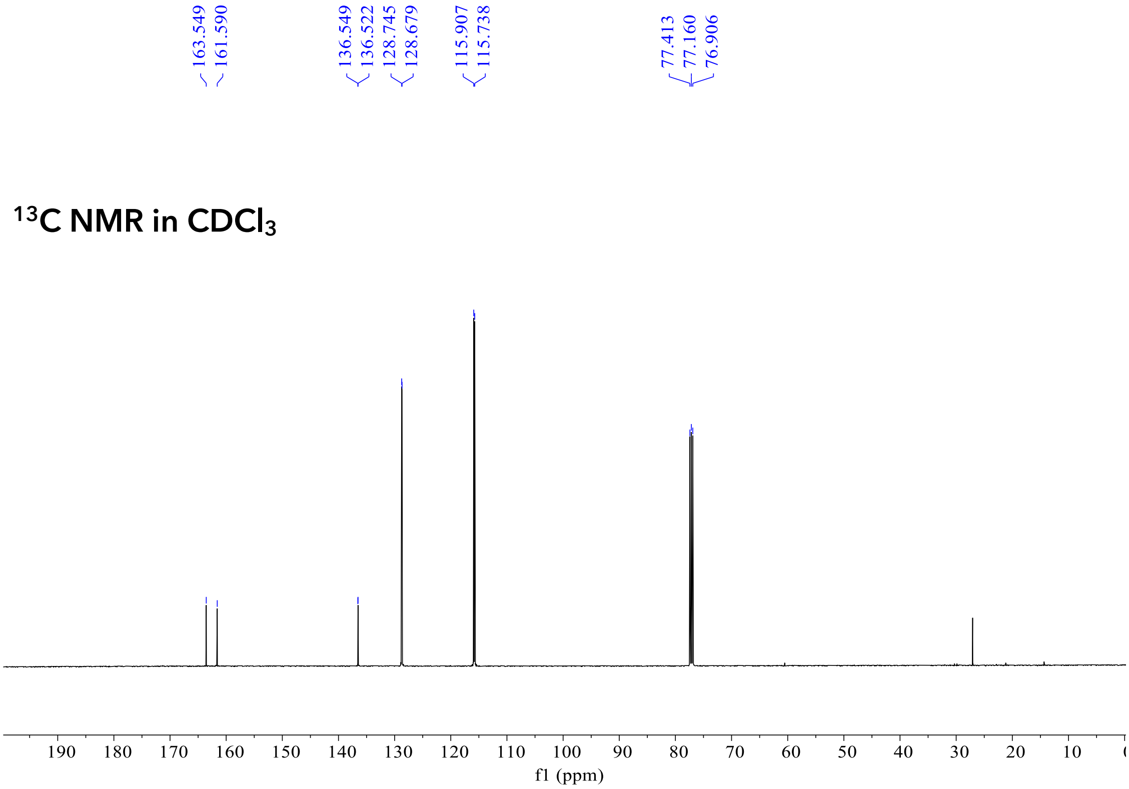

DEPT135 in $CDCl_3$

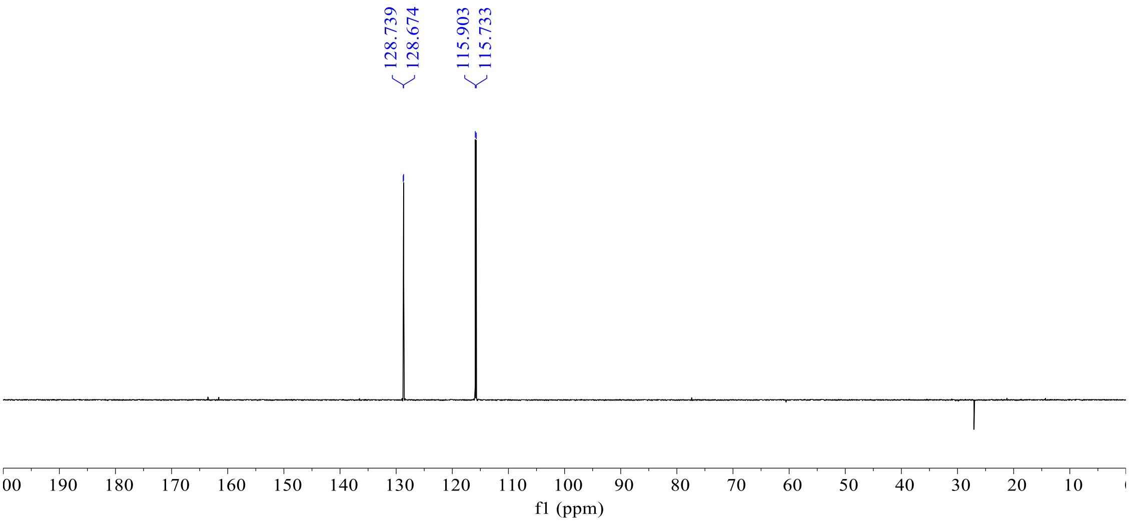

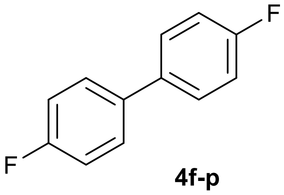

4f-p

^{19}F NMR in $CDCl_3$

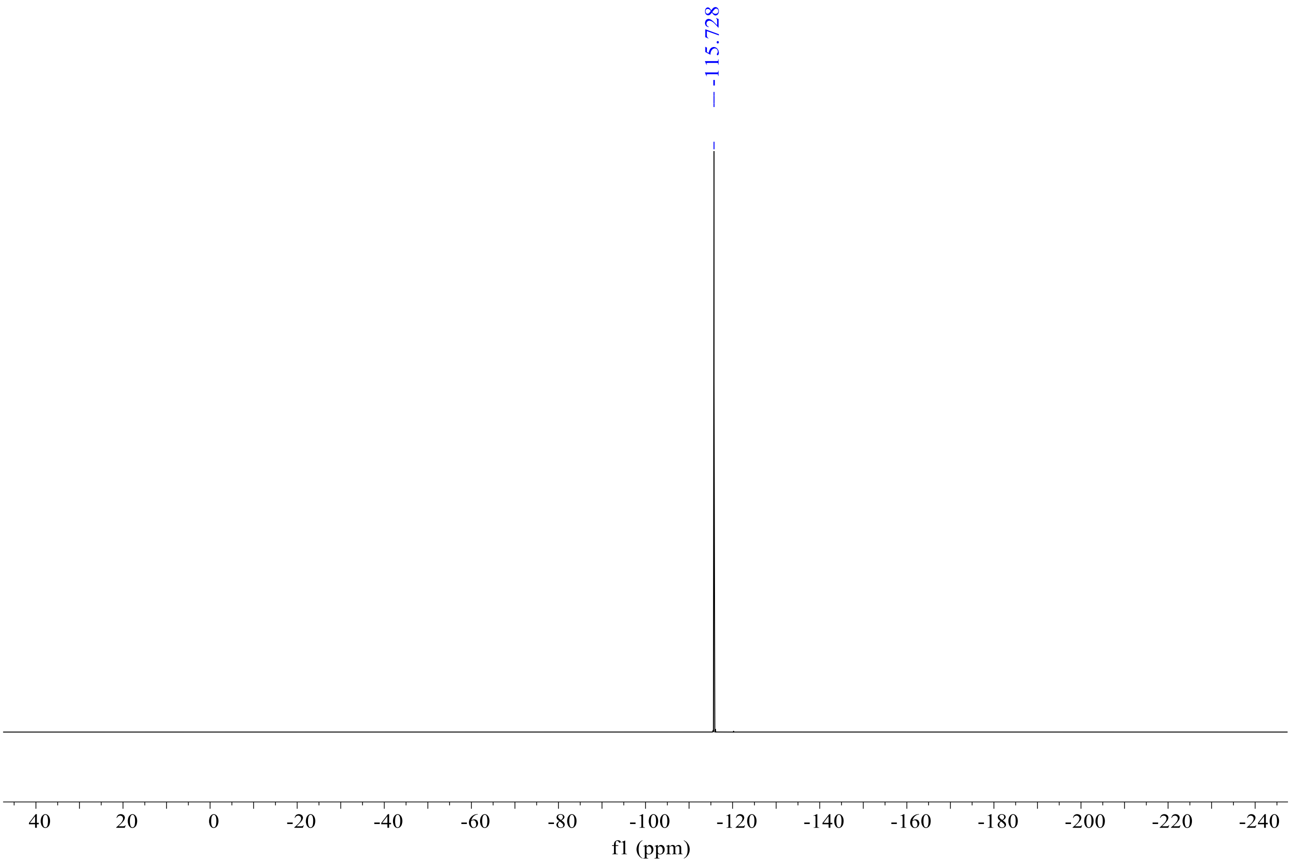

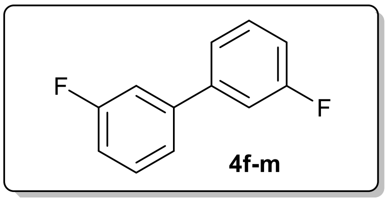

^{1}H-NMR in $CDCl_3$

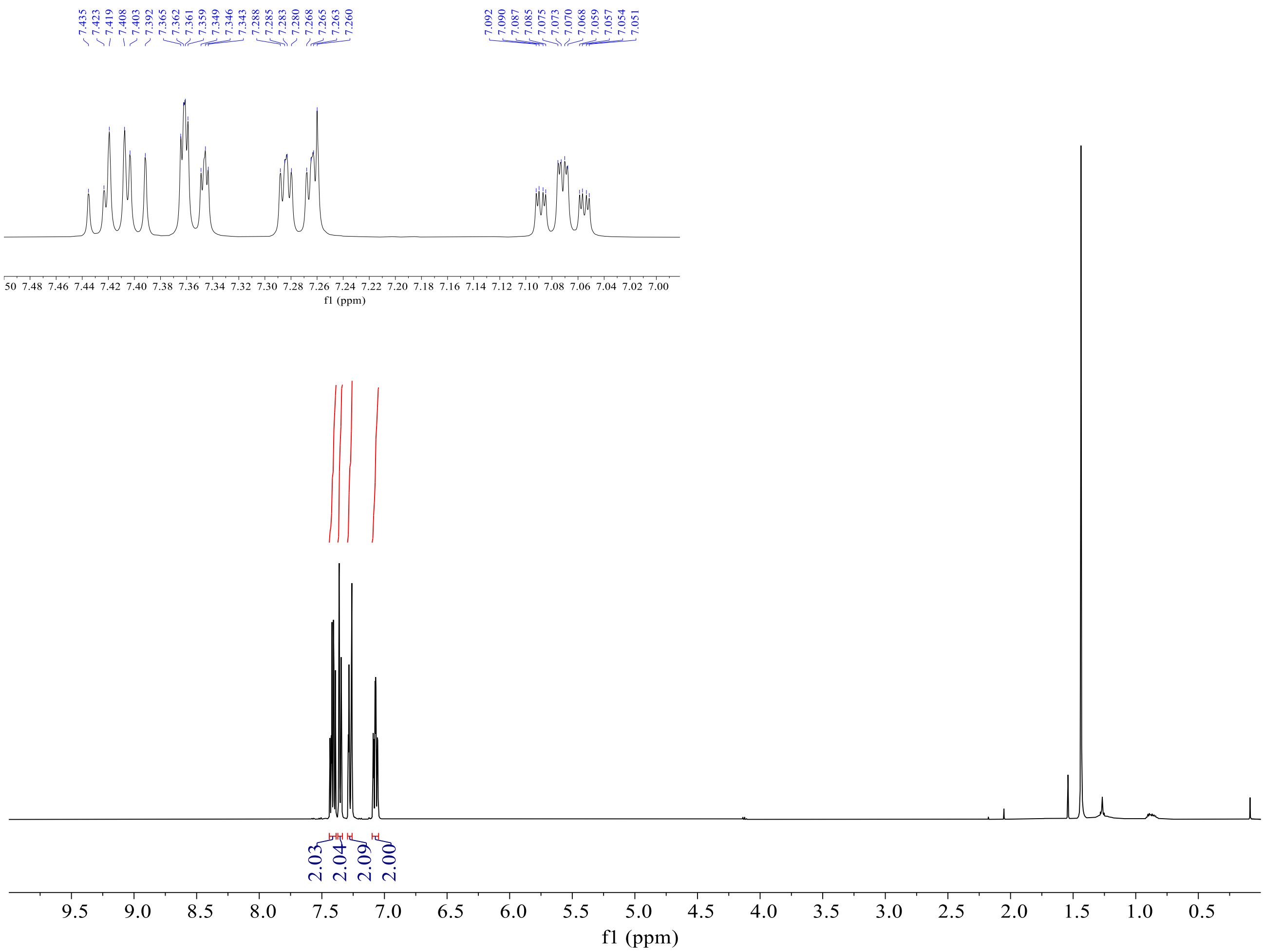

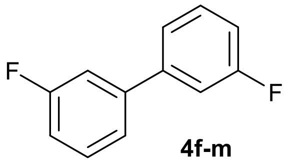

4f-m

^{1}H-NMR in $CDCl_3$ – list of peaks

Label	Intensity	Frequency(ppm)	Frequency(Hz)
1	469	7.435	3719.386
2	473	7.423	3713.452
3	1101	7.419	3711.418
4	1106	7.408	3705.569
5	858	7.403	3703.449
6	857	7.392	3697.600
7	1032	7.365	3684.037
8	1384	7.362	3682.935
9	1410	7.361	3682.257
10	1205	7.359	3681.239
11	636	7.349	3676.238
12	887	7.346	3674.542
13	669	7.343	3673.440
14	689	7.288	3645.805
15	780	7.285	3644.194
16	845	7.283	3643.177
17	680	7.280	3641.566
18	669	7.268	3635.717
19	782	7.265	3634.107
20	879	7.263	3633.089
21	1315	7.260	3631.713
22	446	7.092	3547.725
23	472	7.090	3546.623
24	457	7.087	3545.097
25	419	7.085	3544.080
26	734	7.075	3539.417
27	741	7.073	3538.146
28	780	7.070	3536.789
29	674	7.068	3535.603
30	400	7.059	3531.110
31	429	7.057	3530.008
32	420	7.054	3528.482
33	388	7.051	3635.717

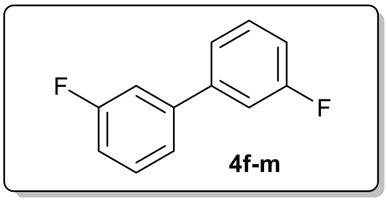

^{13}C NMR in $CDCl_3$

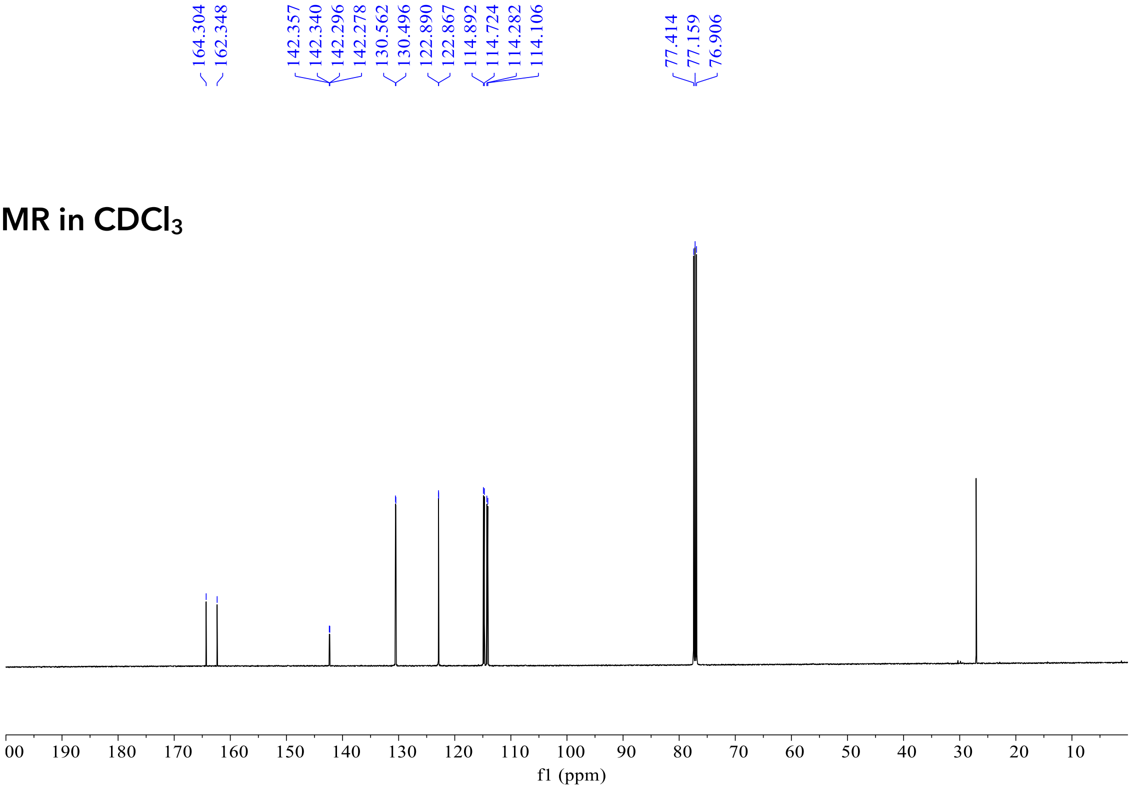

DEPT135 in $CDCl_3$

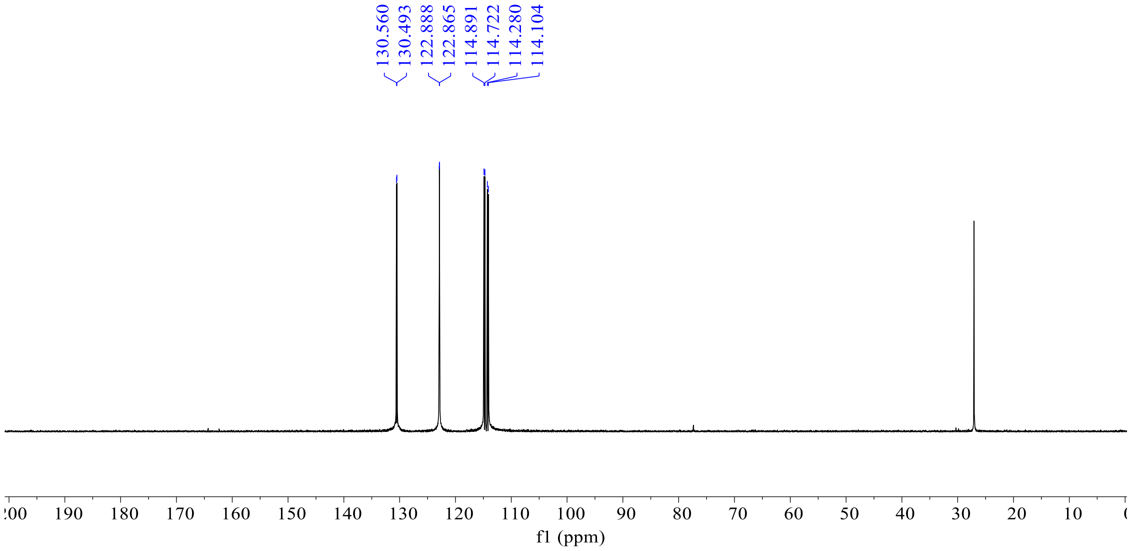

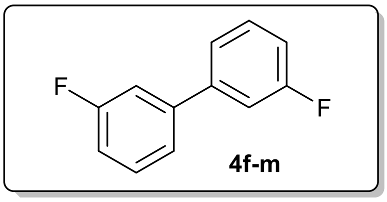

^{19}F NMR in $CDCl_3$

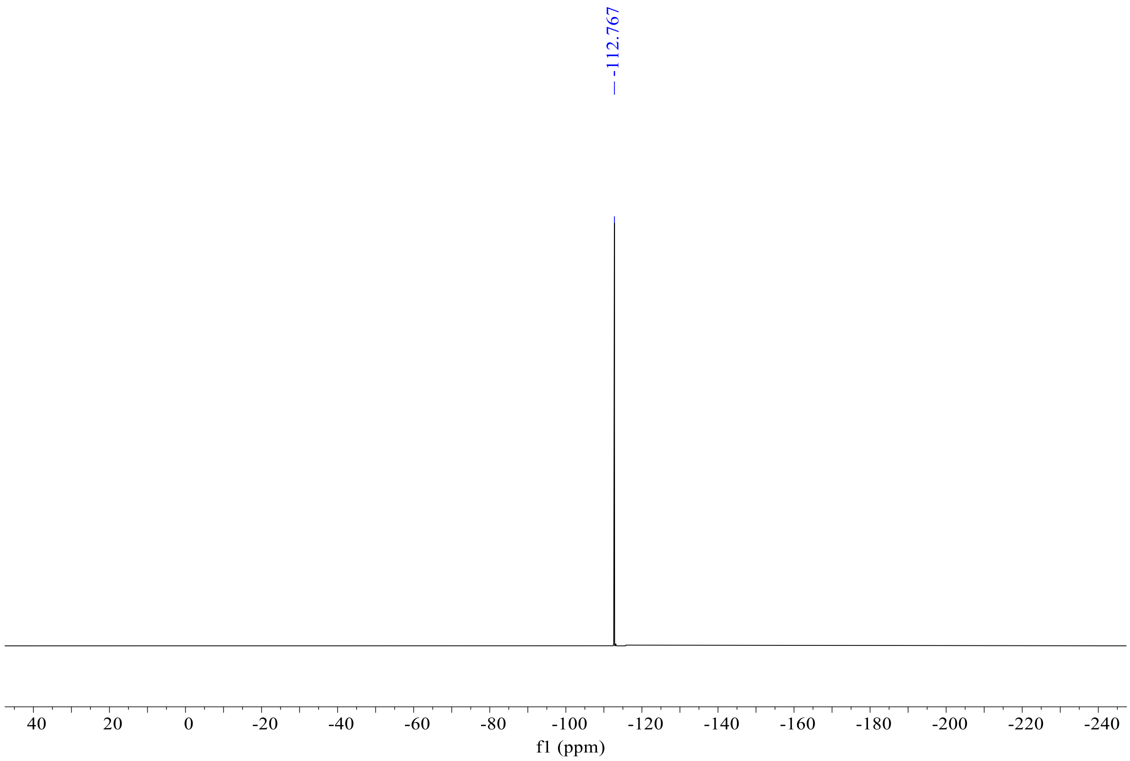

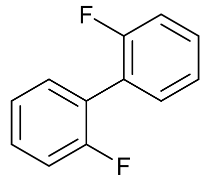

4f-o

^{1}H-NMR in $CDCl_3$

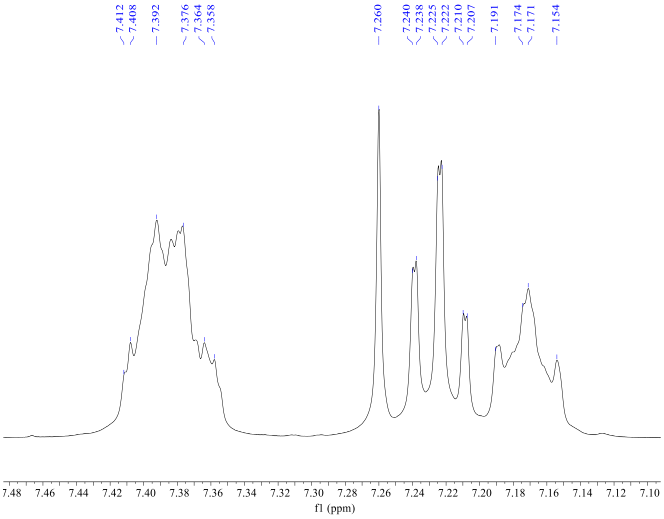

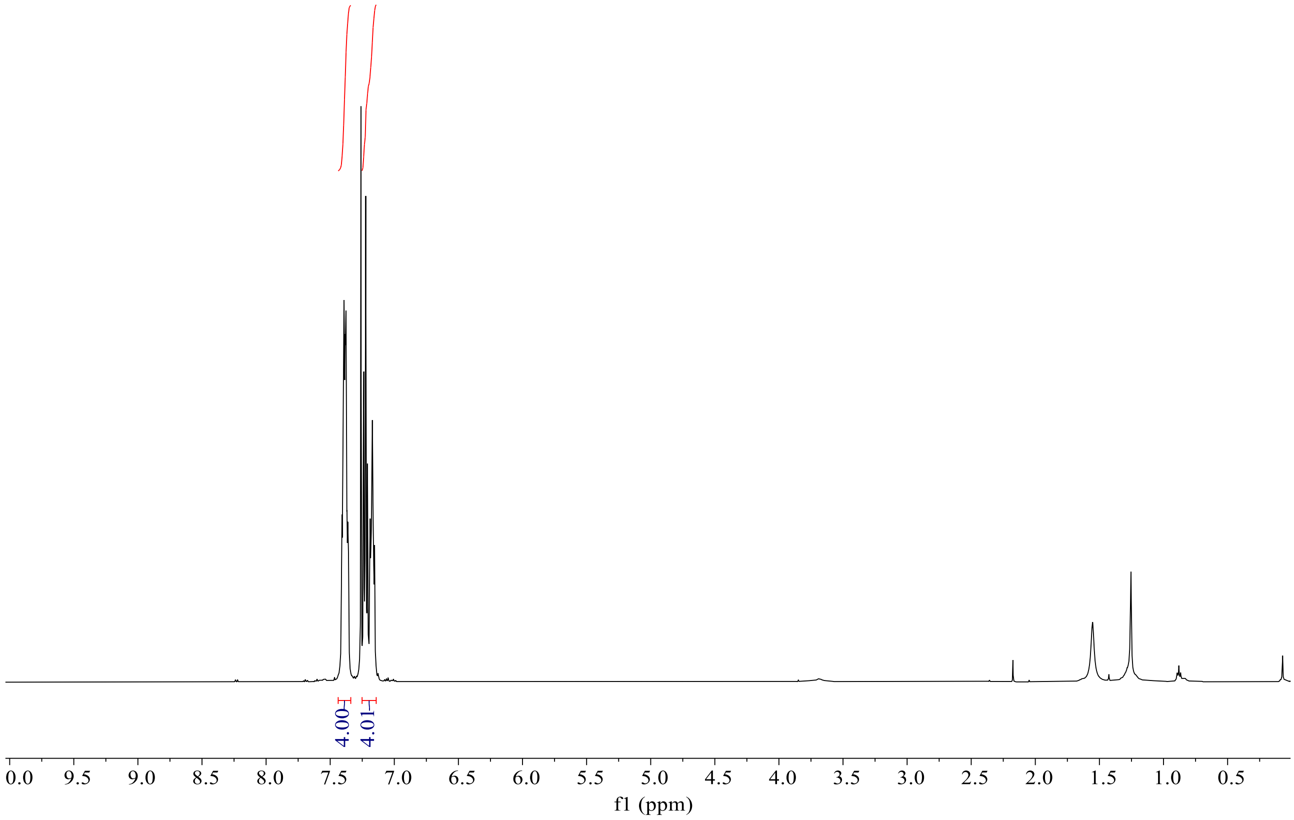

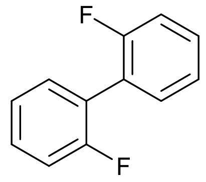

4f-o

^{1}H-NMR in $CDCl_3$ – list of peaks

Label	Intensity	Frequency(ppm)	Frequency(Hz)
1	878	7.412	3706.881
2	1570	7.408	3704.897
3	4823	7.392	3697.115
4	3885	7.376	3689.181
5	1606	7.364	3682.925
6	1181	7.358	3679.873
7	9711	7.260	3630.958
8	3945	7.240	3620.974
9	4327	7.238	3619.753
10	6714	7.225	3613.497
11	7333	7.222	3612.124
12	2781	7.210	3605.868
13	2668	7.207	3604.647
14	1198	7.191	3596.255
15	2684	7.174	3588.167
16	2721	7.171	3586.489
17	1471	7.154	3577.944

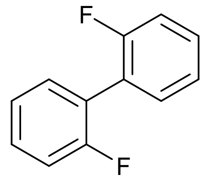

4f-o

^{13}C NMR in $CDCl_3$

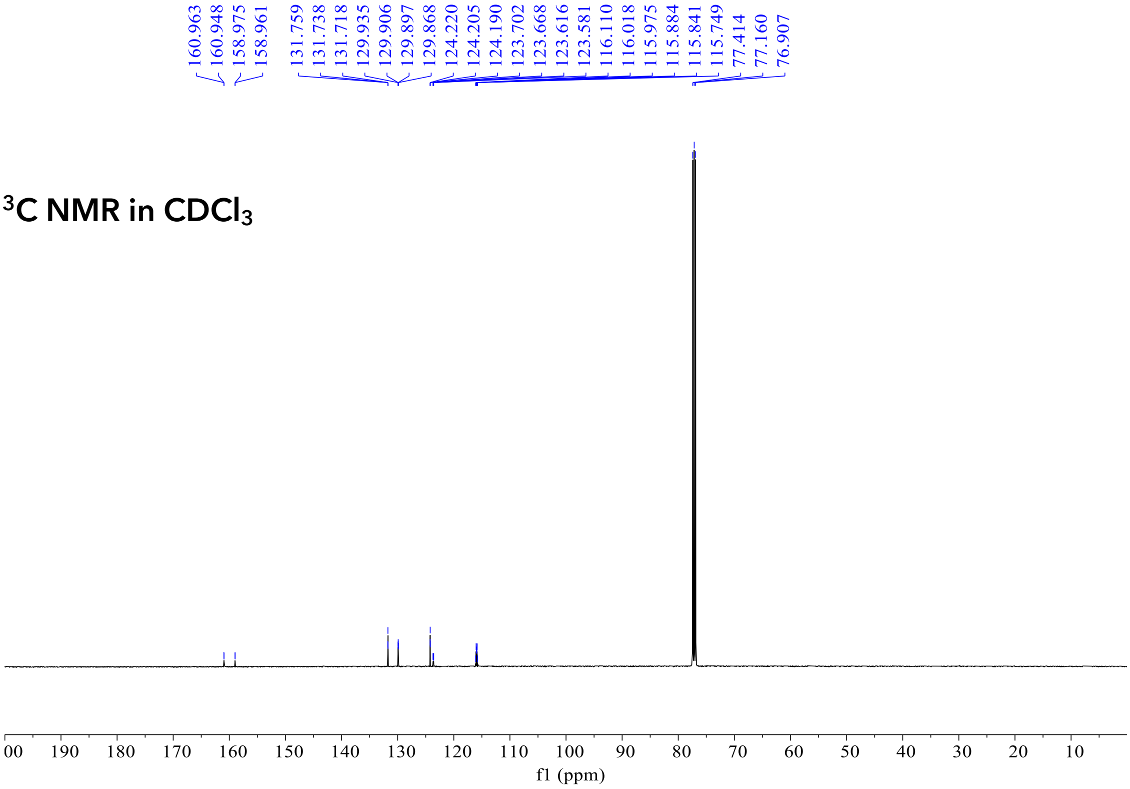

DEPT135 in $CDCl_3$

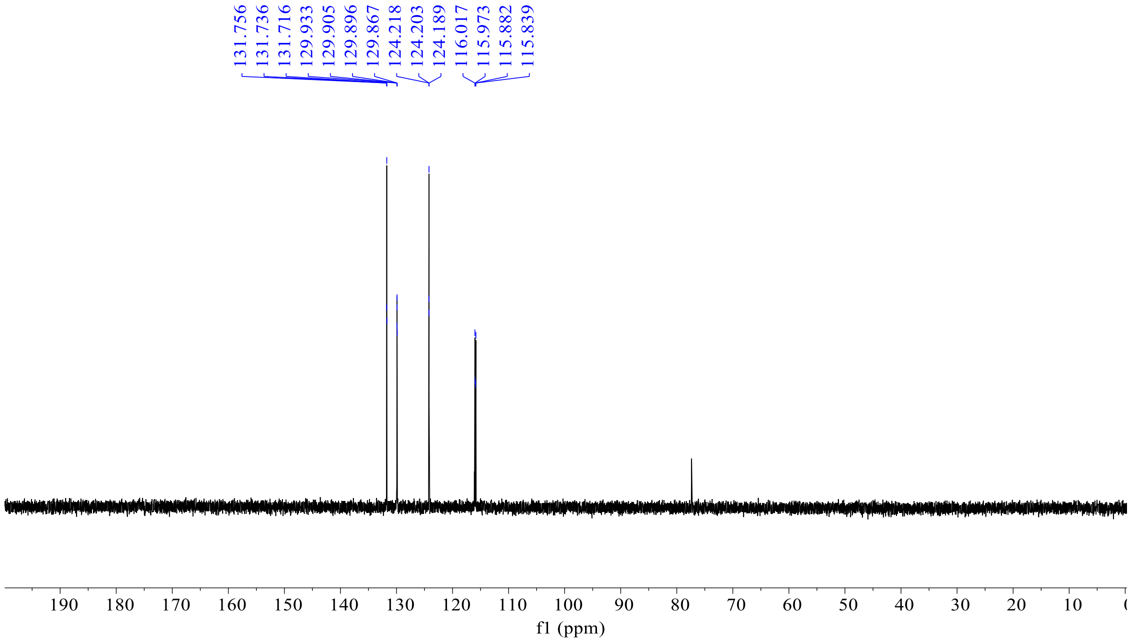

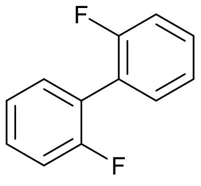

4f-o

^{19}F NMR in $CDCl_3$

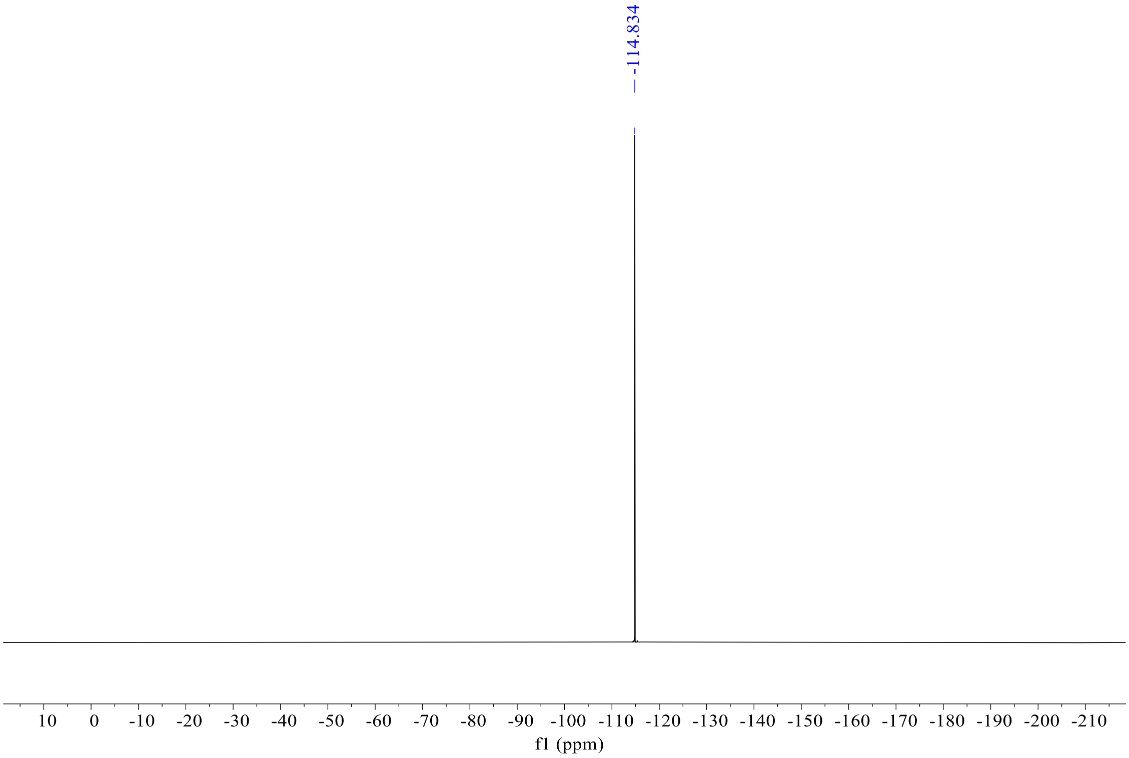

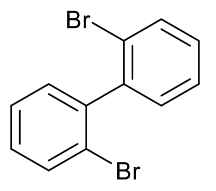

4h-o

^{1}H-NMR in $CDCl_3$

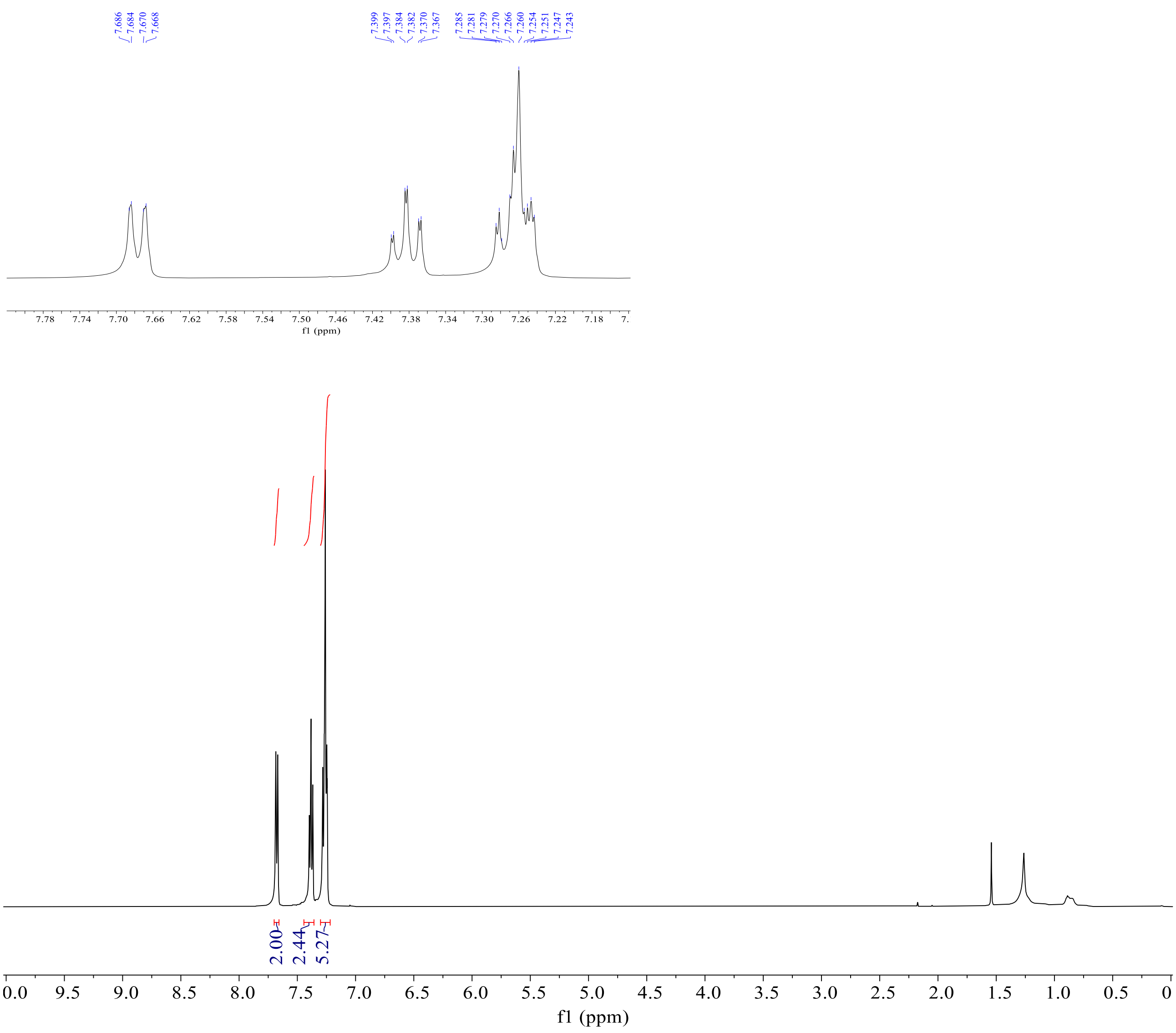

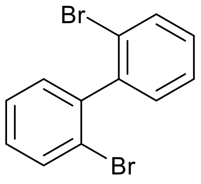

4h-o

^{1}H-NMR in $CDCl_3$ – list of peaks

Label	Intensity	Frequency(ppm)	Frequency(Hz)
1	2094	7.686	3844.022
2	1956	7.684	3842.816
3	2093	7.670	3836.213
4	2465	7.668	3834.752
5	1145	7.399	3700.689
6	1170	7.397	3699.413
7	2672	7.384	3693.209
8	2989	7.382	3691.902
9	1734	7.370	3685.761
10	2046	7.367	3684.396
11	1639	7.285	3643.401
12	2045	7.281	3641.664
13	438	7.279	3640.290
14	1874	7.270	3635.865
15	4262	7.266	3633.890
16	8918	7.260	3630.950
17	1114	7.254	3627.988
18	1828	7.251	3626.252
19	2671	7.247	3624.268
20	1927	7.243	3622.465

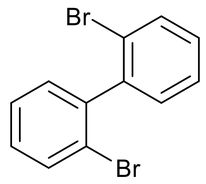

4h-o

^{13}C NMR in $CDCl_3$

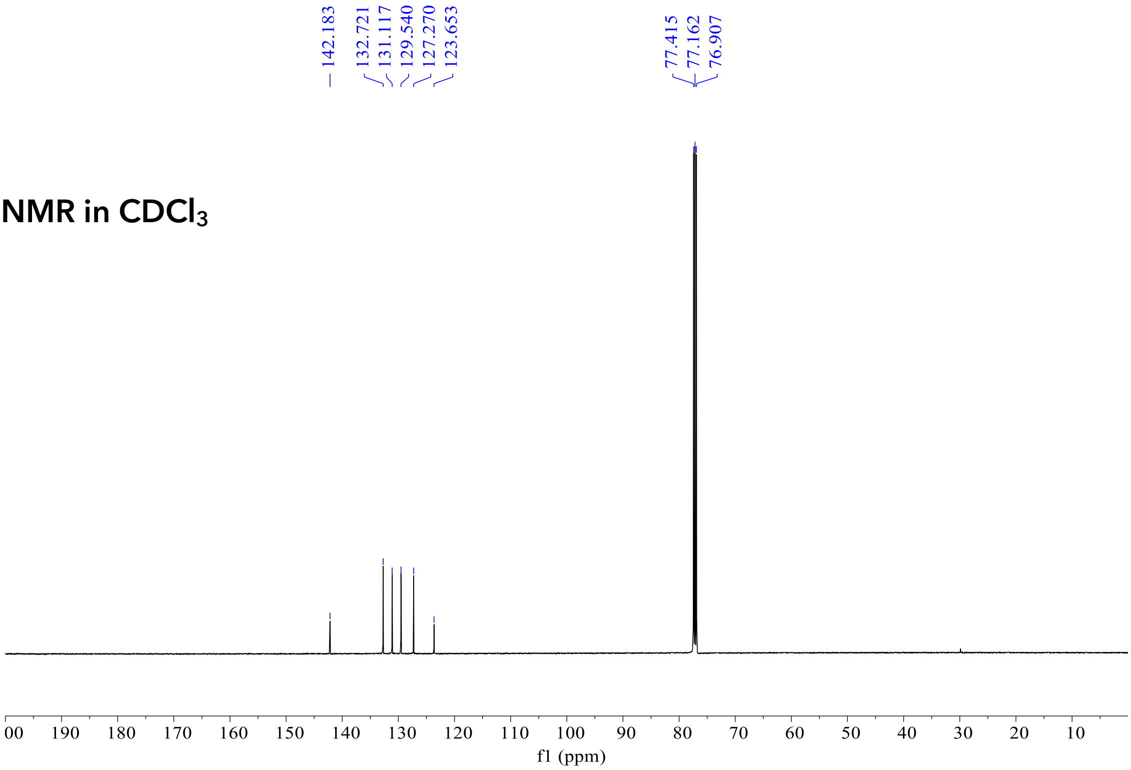

DEPT135 in $CDCl_3$

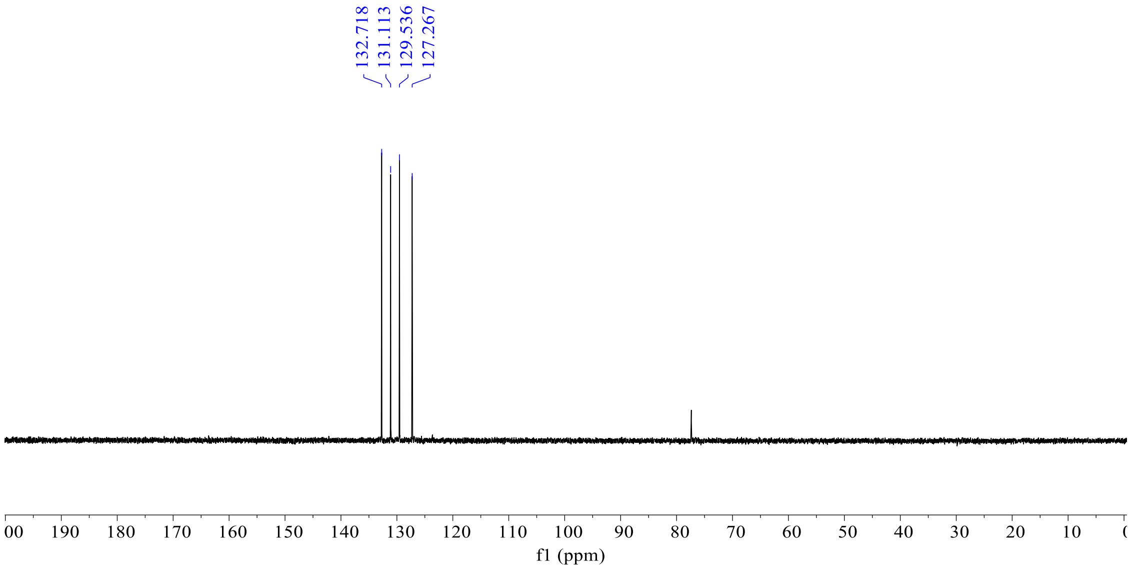

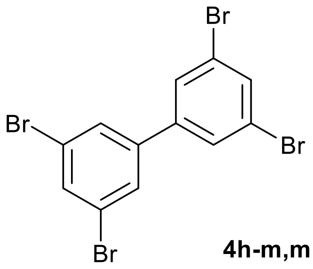

4h-m,m

^{1}H-NMR in $CDCl_3$

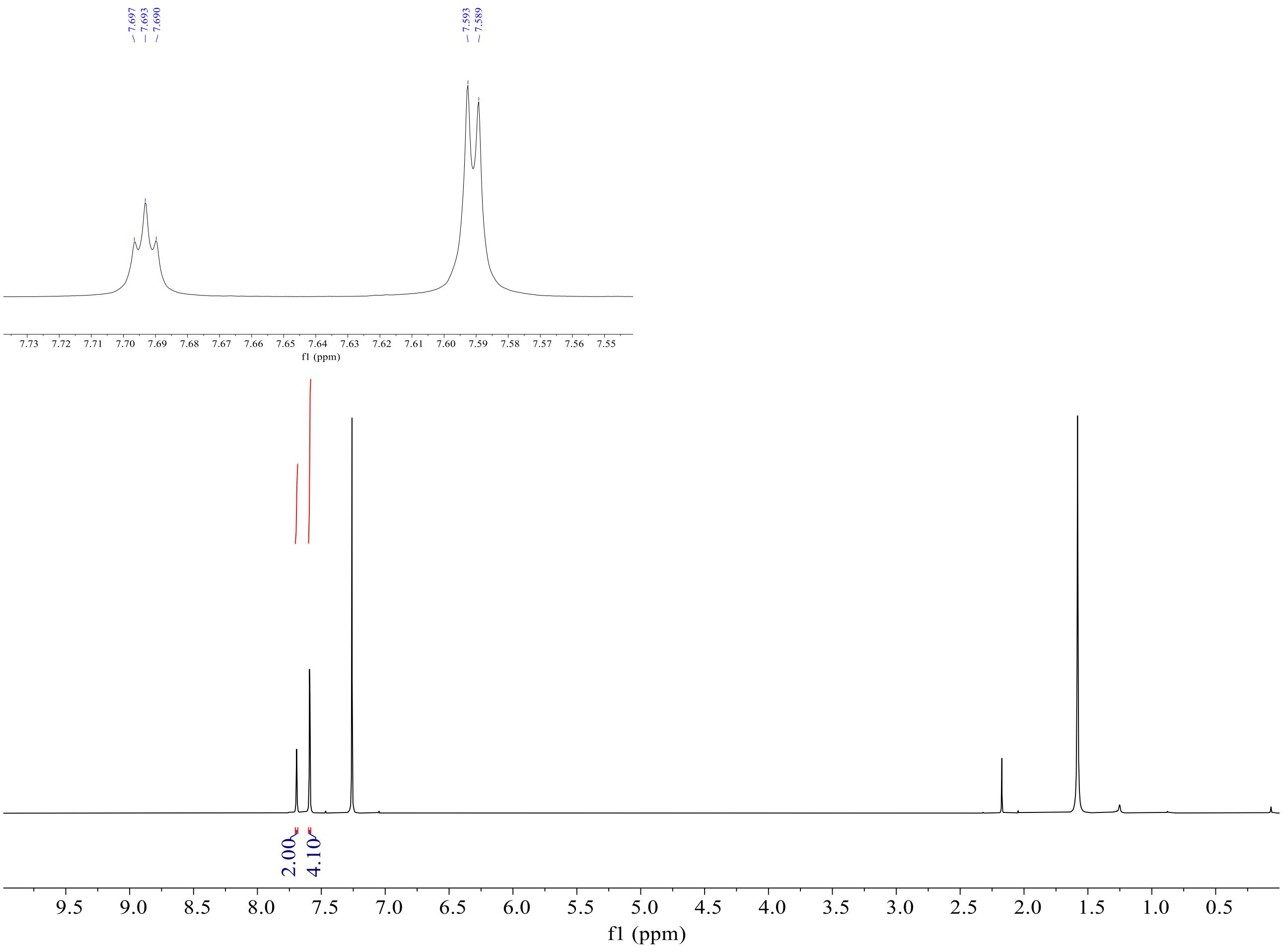

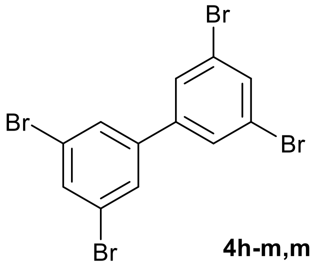

4h-m,m

^{1}H-NMR in $CDCl_3$ – list of peaks

Label	Intensity	Frequency(ppm)	Frequency(ppm)
1	3860	7.697	3849.293
2	7291	7.693	3847.582
3	3966	7.690	3845.869
4	18496	7.593	3797.261
5	14892	7.589	3795.583
6	52319	7.260	3630.961

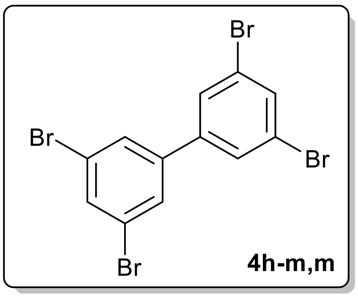

^{13}C NMR in $CDCl_3$

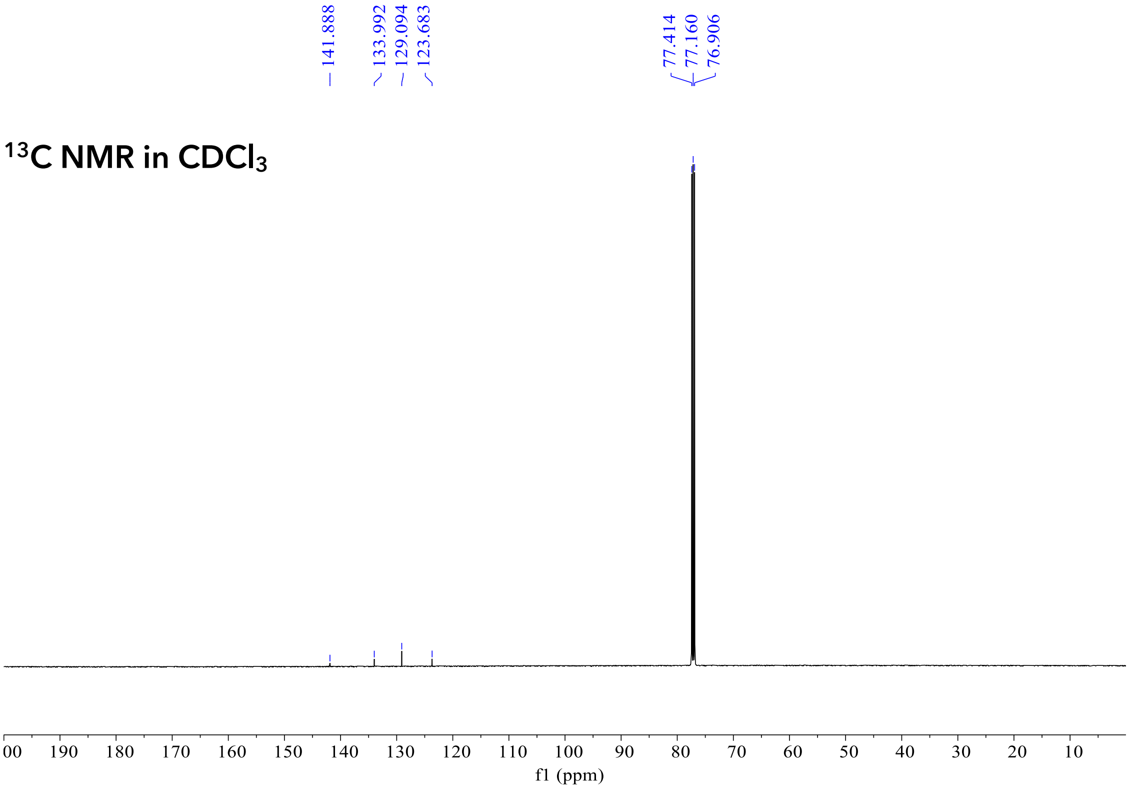

DEPT135 in $CDCl_3$

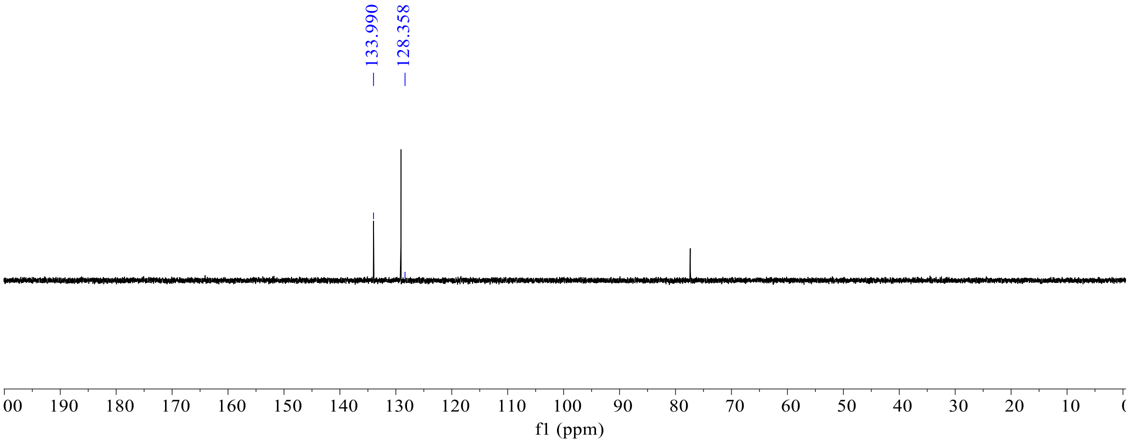

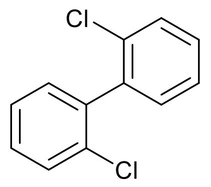

4i-o

^{1}H-NMR in $CDCl_3$

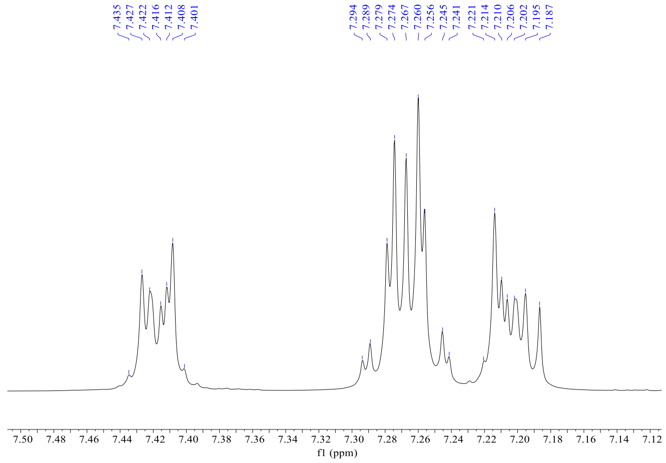

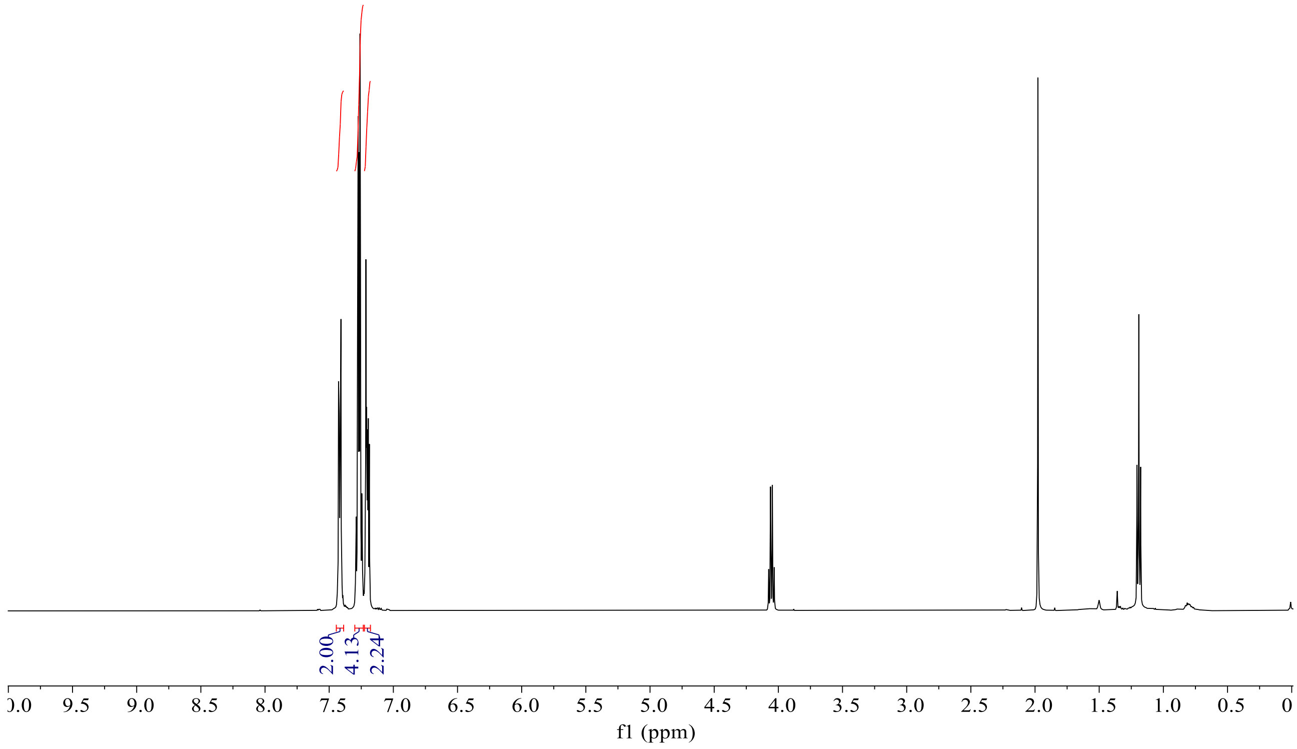

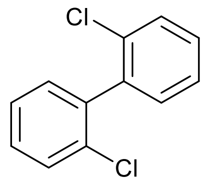

4i-o

^{1}H-NMR in $CDCl_3$ – list of peaks

Label	Intensity	Frequency(ppm)	Frequency(ppm)
1	675	7.435	3718.346
2	7710	7.427	3714.379
3	6649	7.422	3712.090
4	5966	7.416	3708.733
5	6484	7.412	3706.902
6	9958	7.408	3705.071
7	878	7.401	3701.562
8	1570	7.294	3647.851
9	2893	7.289	3645.409
10	8250	7.279	3640.374
11	16201	7.274	3638.085
12	16936	7.267	3634.576
13	22009	7.260	3630.949
14	10564	7.256	3628.930
15	3440	7.245	3623.589
16	1444	7.241	3621.606
17	900	7.221	3611.230
18	12894	7.214	3607.873
19	7516	7.210	3605.737
20	6009	7.206	3604.058
21	5256	7.202	3601.922
22	5679	7.195	3598.565
23	5717	7.187	3594.292

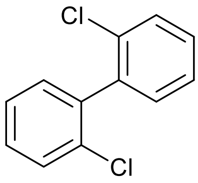

4i-o

^{13}C NMR in $CDCl_3$

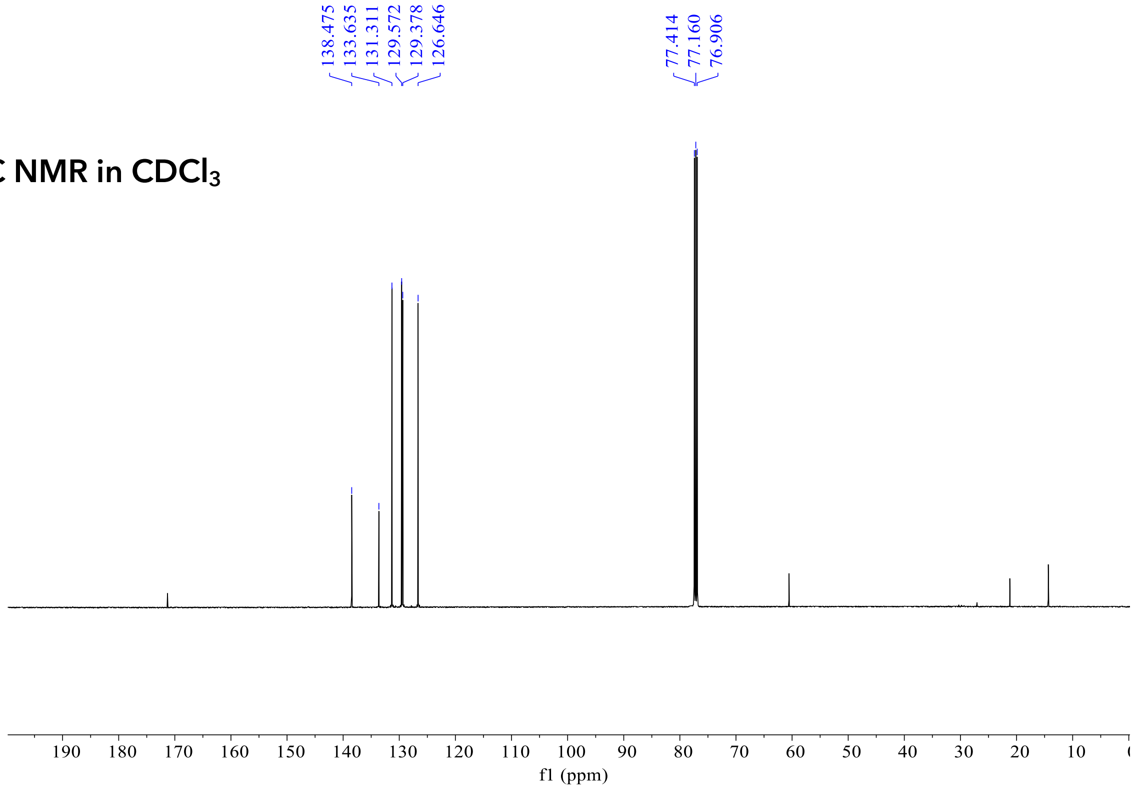

DEPT135 in $CDCl_3$

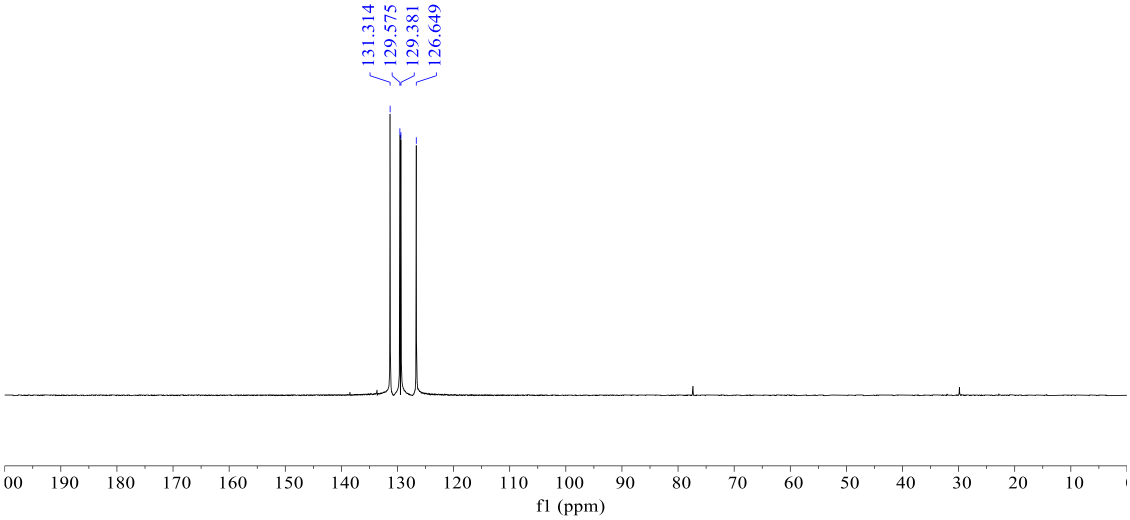

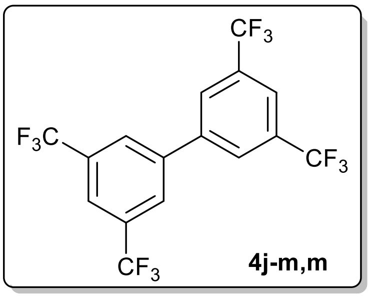

^{1}H-NMR in $CDCl_3$

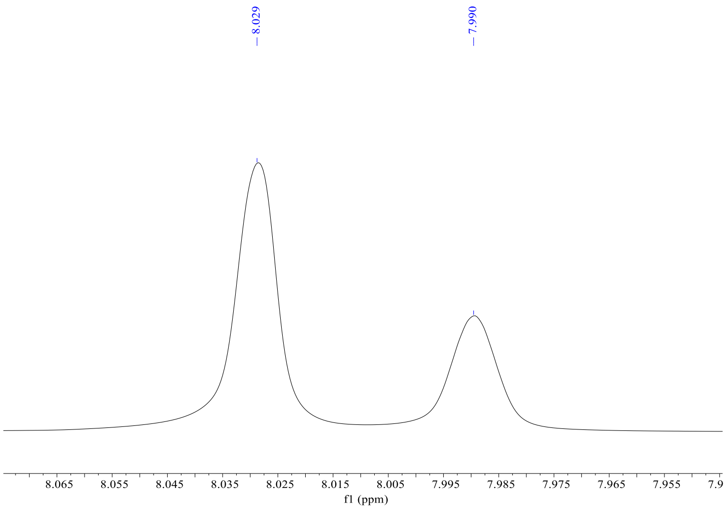

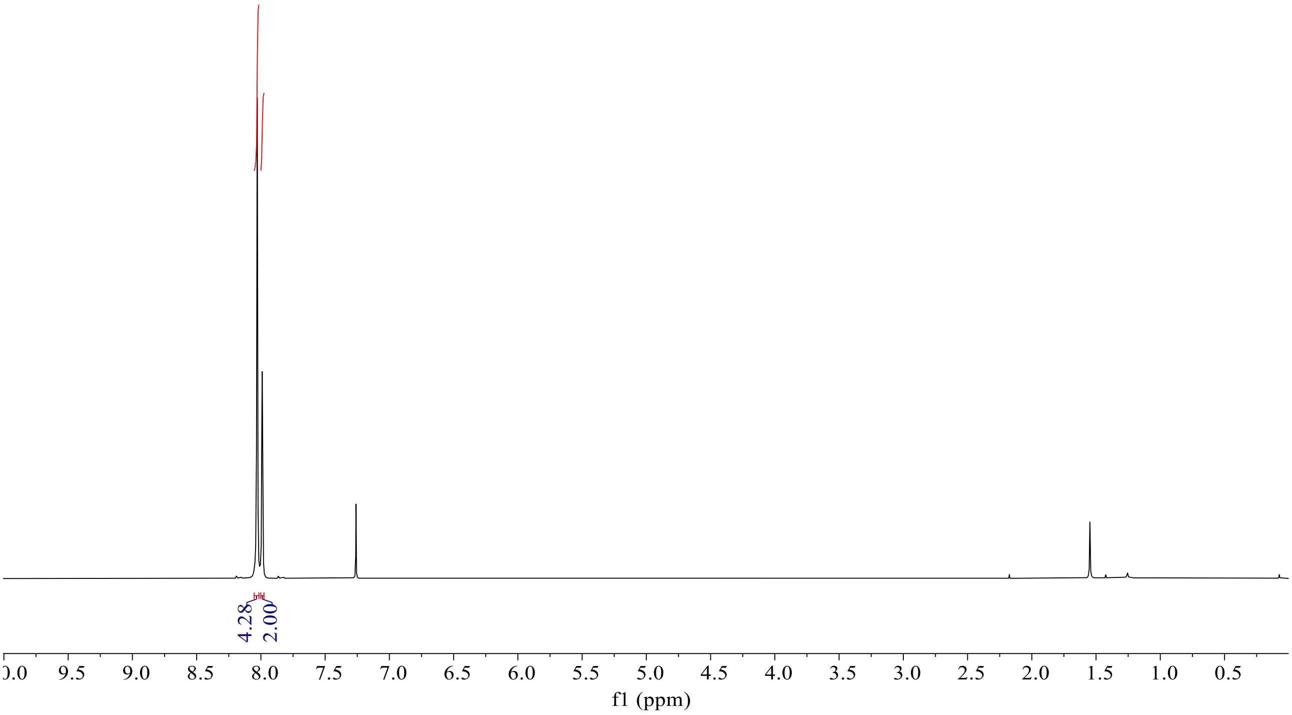

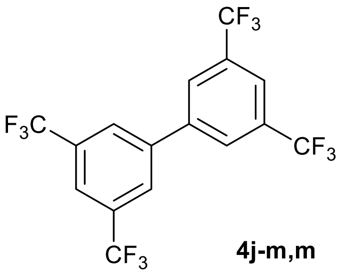

4j-m,m

^{1}H-NMR in $CDCl_3$ – list of peaks

Label	Intensity	Frequency(ppm)	Frequency(ppm)
1	26822	8.029	4015.428
2	11709	7.990	3995.812
3	3976	7.260	3630.982

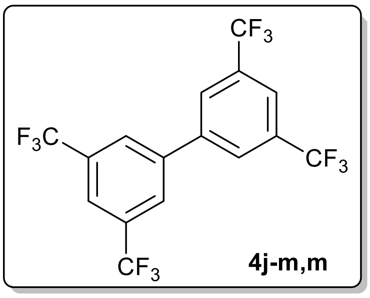

4j-m,m

^{13}C NMR in CDCl$_3$

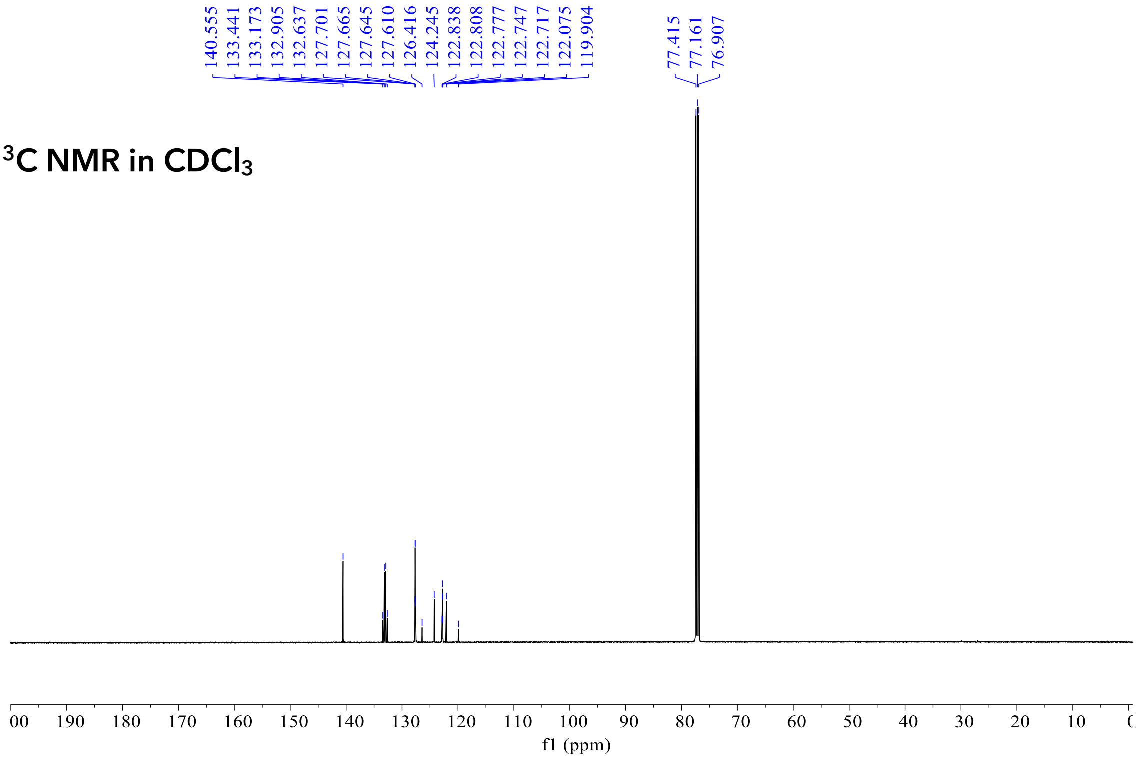

DEPT135 in CDCl$_3$

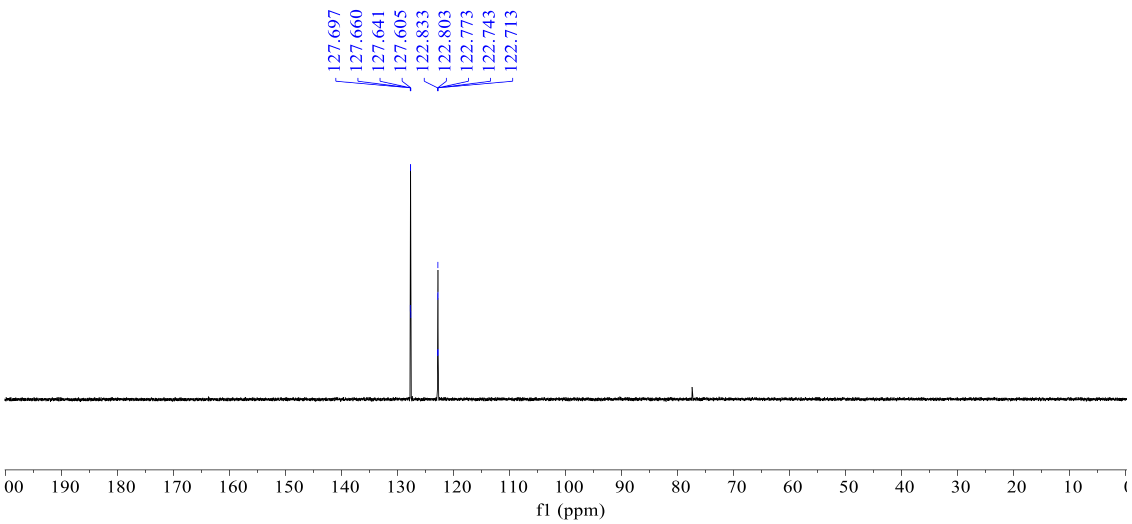

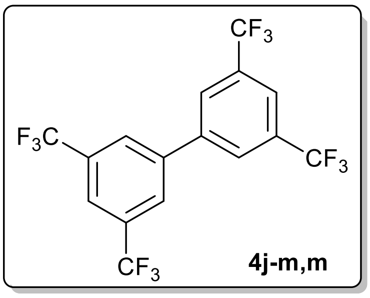

4j-m,m

^{19}F NMR in $CDCl_3$

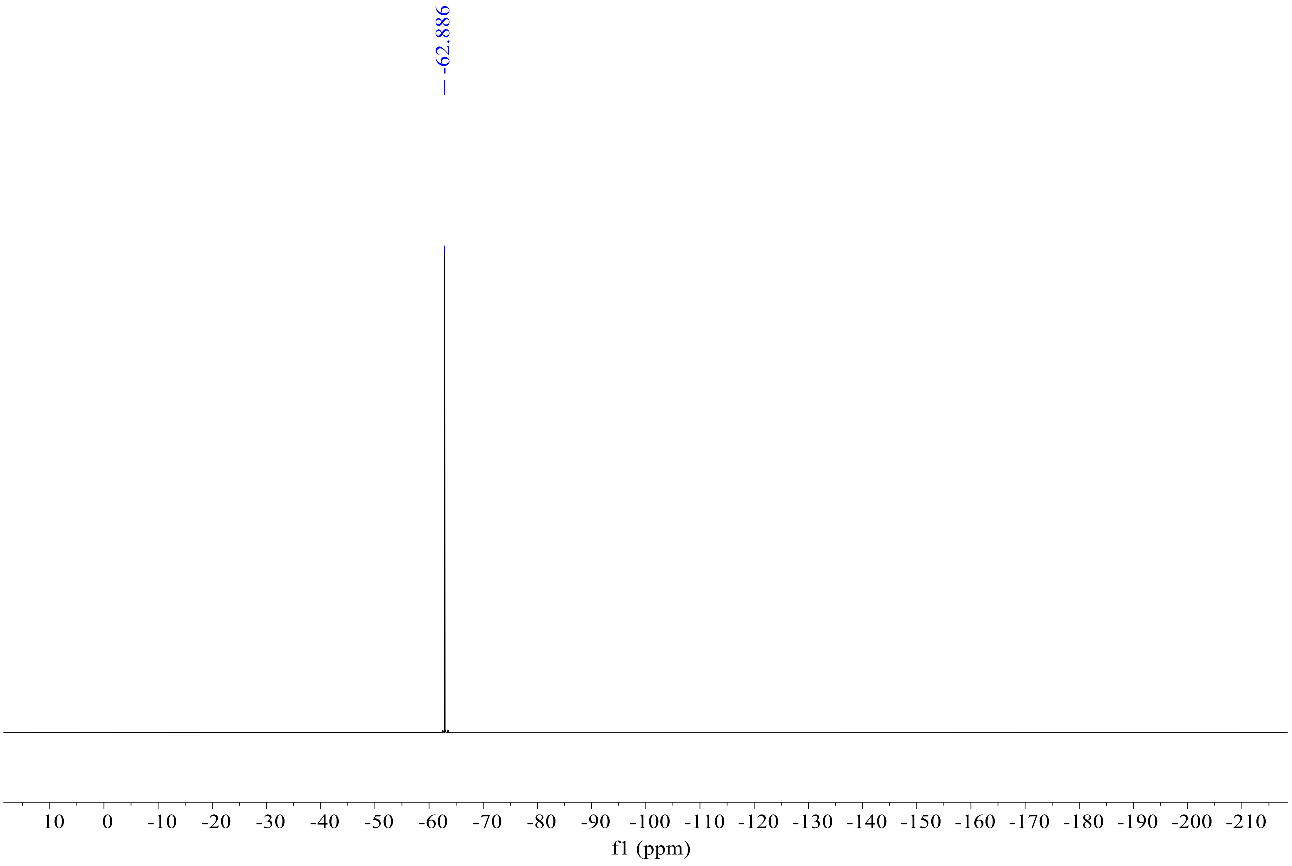

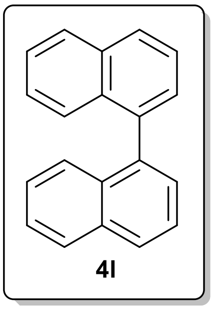

4l

^{1}H-NMR in $CDCl_3$

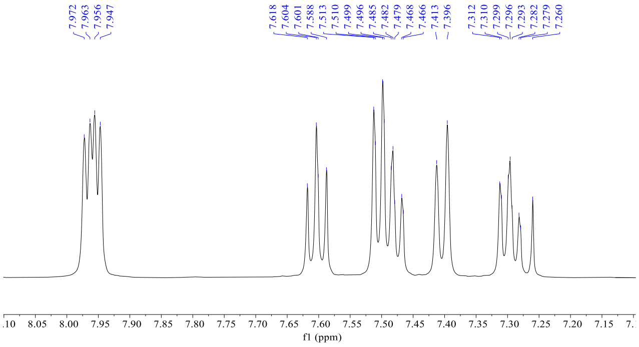

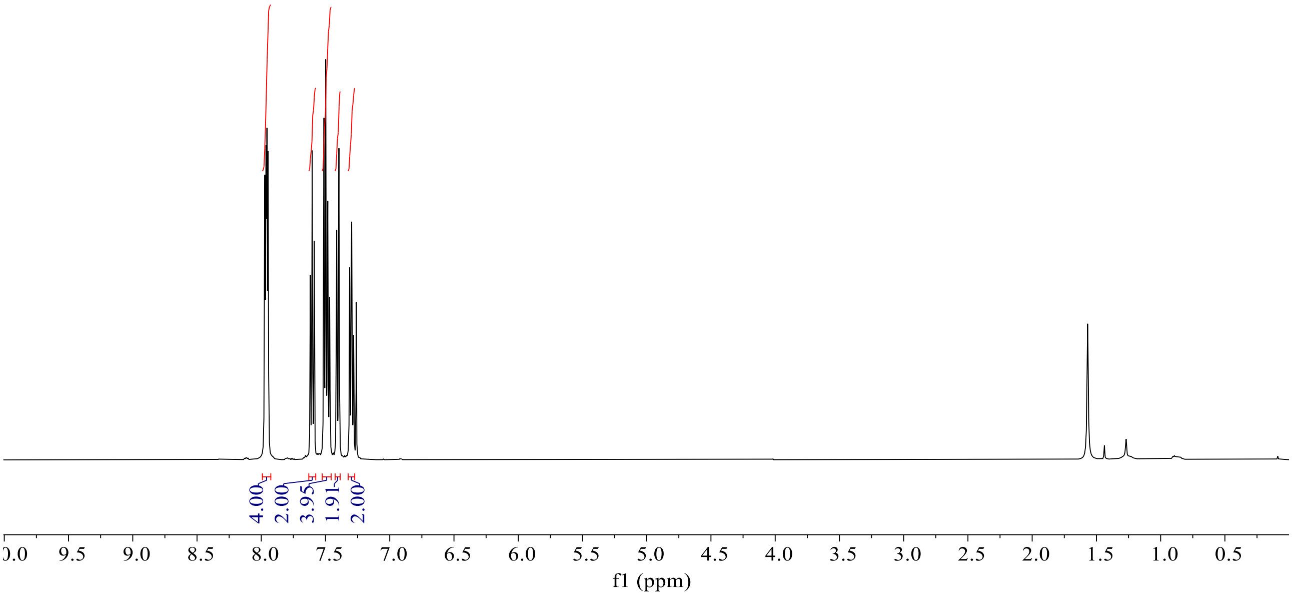

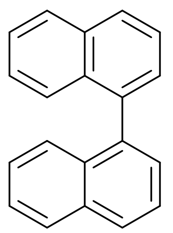

4l

^{1}H-NMR in $CDCl_3$ – list of peaks

Label	Intensity	Frequency(ppm)	Frequency(ppm)
1	7045	7.972	3987.269
2	7311	7.963	3982.676
3	7513	7.956	3978.898
4	7655	7.947	3974.499
5	4899	7.618	3809.979
6	7797	7.604	3802.857
7	2248	7.601	3801.389
8	5676	7.588	3794.824
9	8412	7.513	3757.261
10	2982	7.510	3755.957
11	9546	7.499	3750.243
12	3693	7.496	3748.932
13	4051	7.485	3743.339
14	4768	7.482	3741.974
15	1555	7.479	3740.573
16	3964	7.468	3735.014
17	1437	7.466	3733.735
18	6117	7.413	3707.279
19	8393	7.396	3698.805
20	4367	7.312	3657.166
21	2294	7.310	3655.797
22	3907	7.299	3650.382
23	4634	7.296	3648.889
24	1811	7.293	3647.333
25	2892	7.282	3641.877
26	1314	7.279	3640.503
27	4217	7.260	3630.995

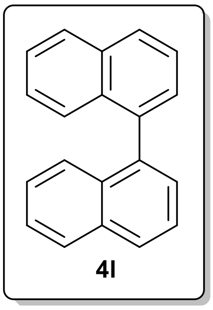

4l

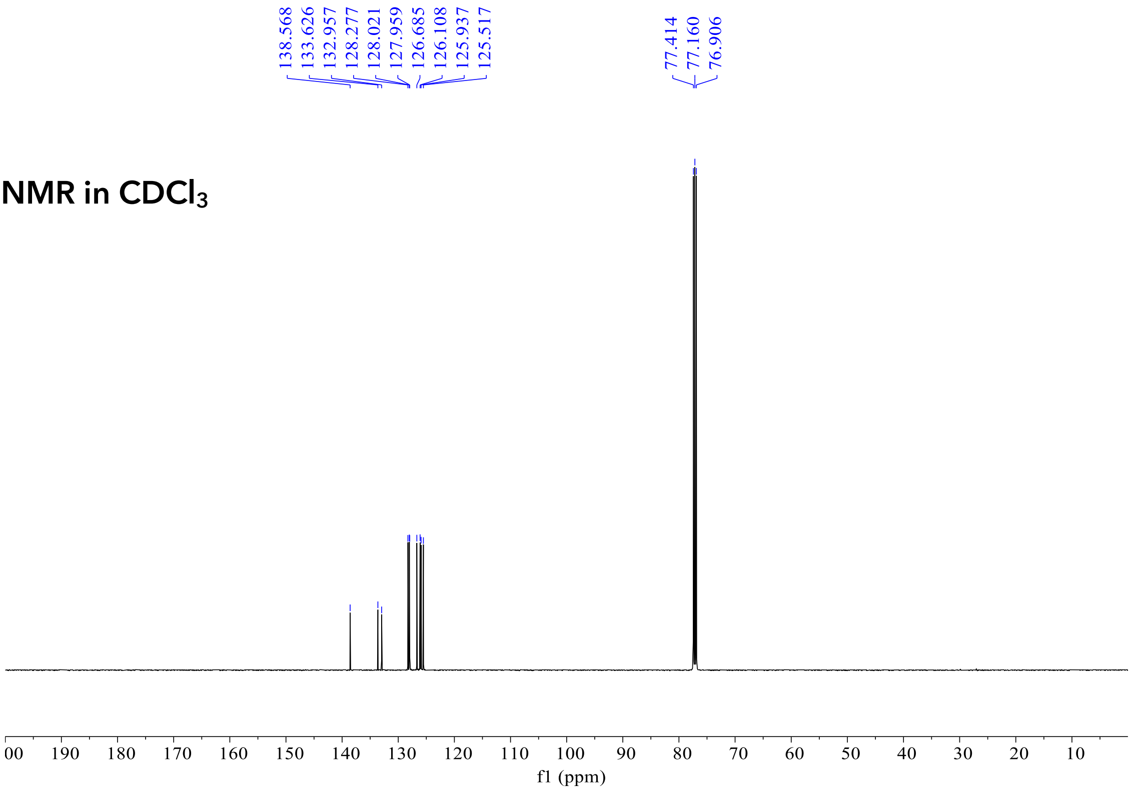

^{13}C NMR in $CDCl_3$

DEPT135 in $CDCl_3$

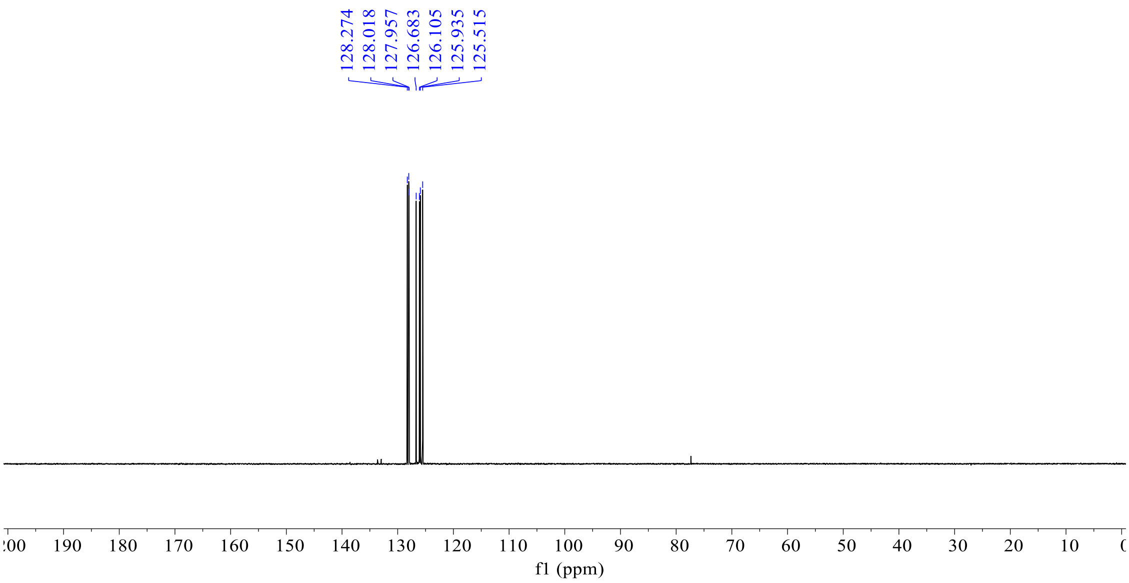

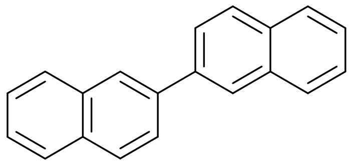

4m

^{1}H-NMR in $CDCl_3$

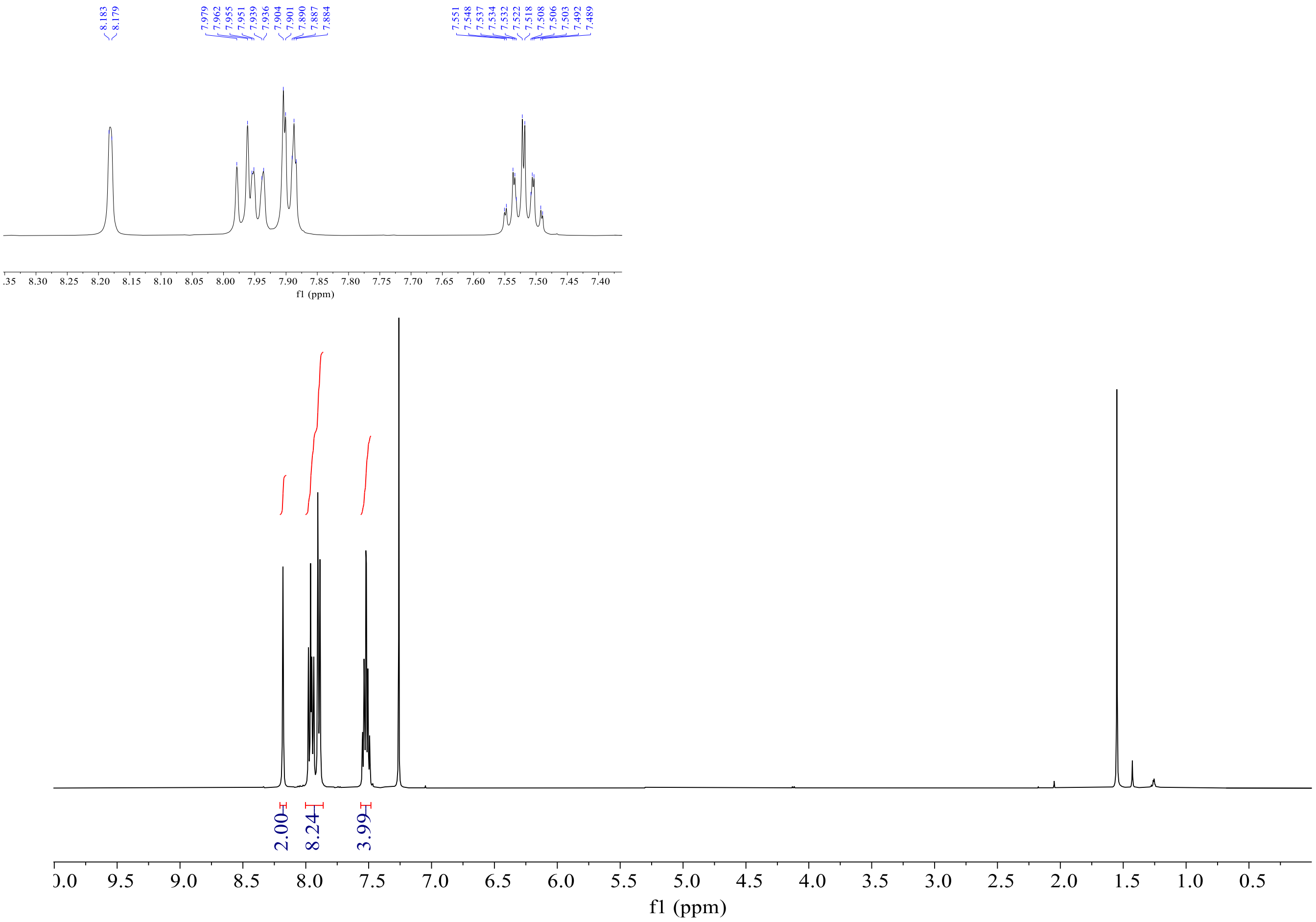

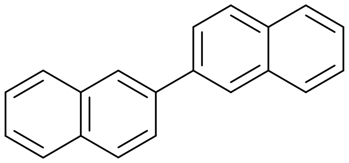

4m

^{1}H-NMR in $CDCl_3$ – list of peaks

Label	Intensity	Frequency(ppm)	Frequency(ppm)
1	12666	8.183	4092.636
2	11895	8.179	4090.347
3	8670	7.979	3990.395
4	12211	7.962	3981.832
5	3252	7.955	3978.302
6	4546	7.951	3976.707
7	3762	7.939	3970.331
8	4769	7.936	3968.887
9	16347	7.904	3953.204
10	9579	7.901	3951.339
11	5744	7.890	3946.150
12	10511	7.887	3944.626
13	5144	7.884	3942.846
14	2247	7.551	3776.289
15	2498	7.548	3774.745
16	5809	7.537	3769.452
17	4905	7.534	3767.929
18	1035	7.532	3766.734
19	12467	7.522	3761.978
20	11157	7.518	3760.008
21	1578	7.508	3755.130
22	4625	7.506	3754.033
23	4728	7.503	3752.622
24	2557	7.492	3747.210
25	1687	7.489	3745.716
26	30011	7.260	3630.905

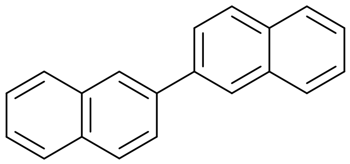

4m

^{13}C NMR in $CDCl_3$

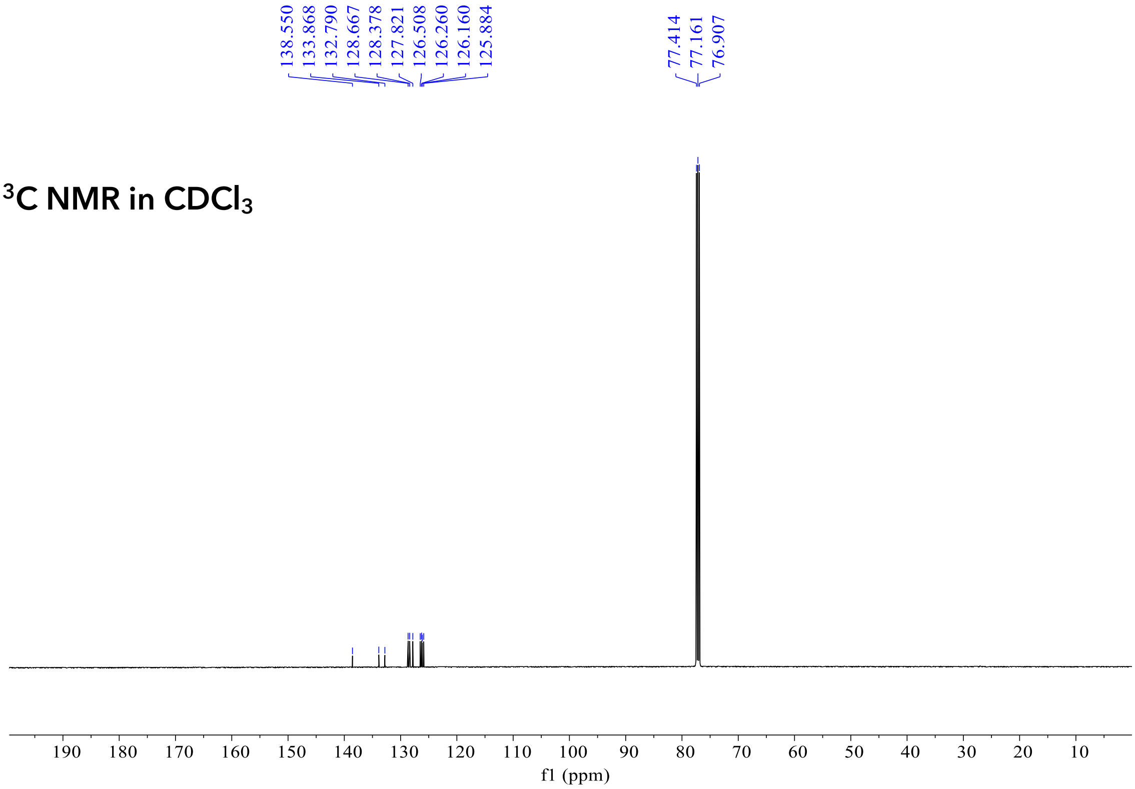

DEPT135 in $CDCl_3$

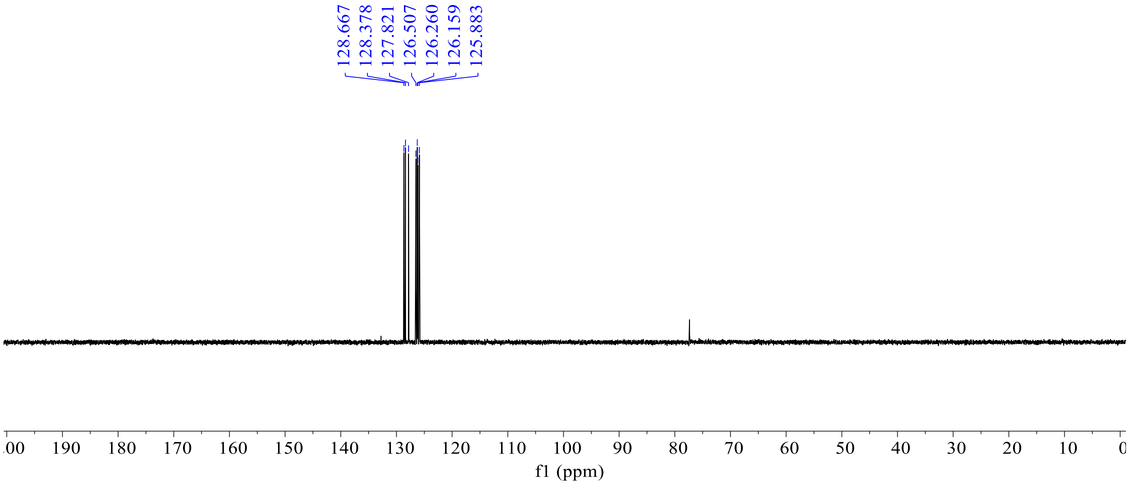

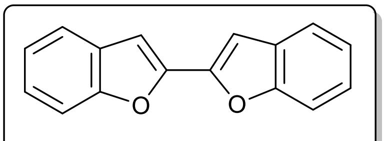

4n

^{1}H-NMR in $CDCl_3$

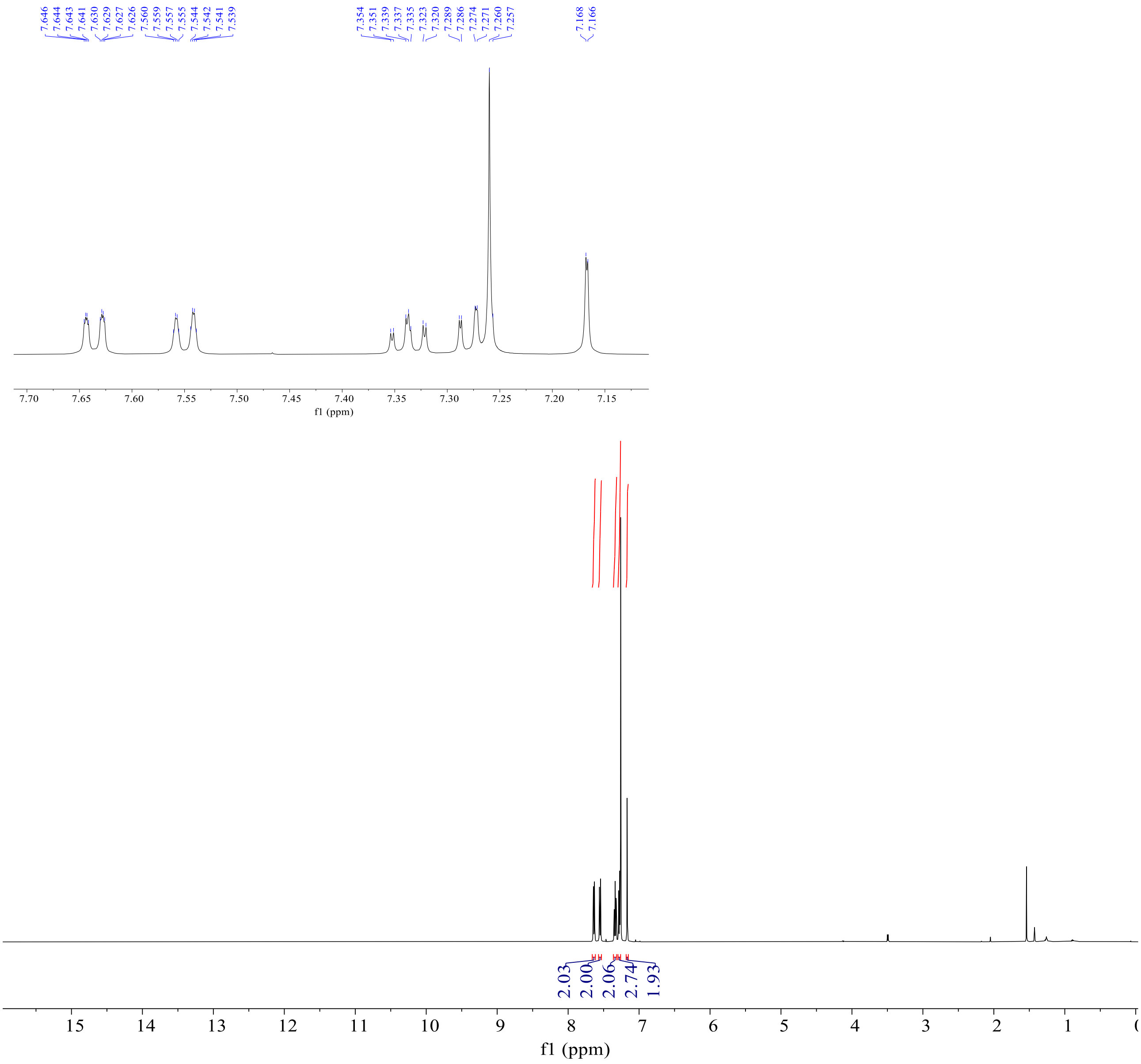

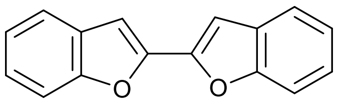

4n

^{1}H-NMR in $CDCl_3$ – list of peaks

Label	Intensity	Frequency(ppm)	Frequency(ppm)
1	2929	7.646	3823.762
2	3016	7.644	3823.026
3	2776	7.643	3822.391
4	3016	7.641	3821.663
5	2582	7.630	3816.094
6	3861	7.629	3815.369
7	3102	7.627	3814.696
8	2712	7.626	3814.004
9	1328	7.560	3781.227
10	4211	7.559	3780.279
11	2832	7.557	3779.472
12	1786	7.555	3778.715
13	1191	7.544	3773.065
14	4562	7.542	3772.183
15	4200	7.541	3771.269
16	1212	7.539	3770.362
17	2615	7.354	3677.884
18	2723	7.351	3676.531
19	3674	7.339	3670.658
20	5200	7.337	3669.380
21	1763	7.335	3668.281
22	3725	7.323	3662.474
23	3279	7.320	3661.091
24	4142	7.289	3645.231
25	4090	7.286	3644.186
26	5428	7.274	3637.709
27	4350	7.271	3636.686
28	41663	7.260	3630.926
29	1547	7.257	3629.226
30	12039	7.168	3584.958
31	10497	7.166	3584.043

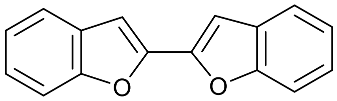

4n

^{13}C NMR in $CDCl_3$

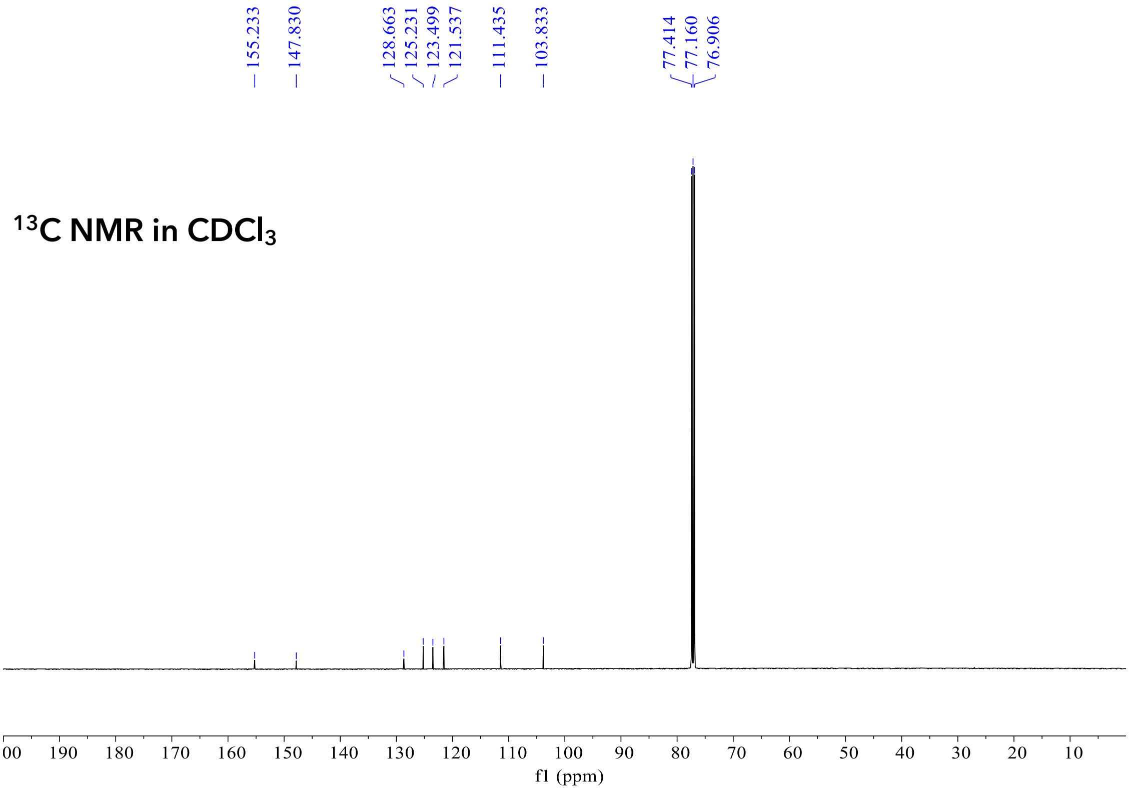

DEPT135 in $CDCl_3$

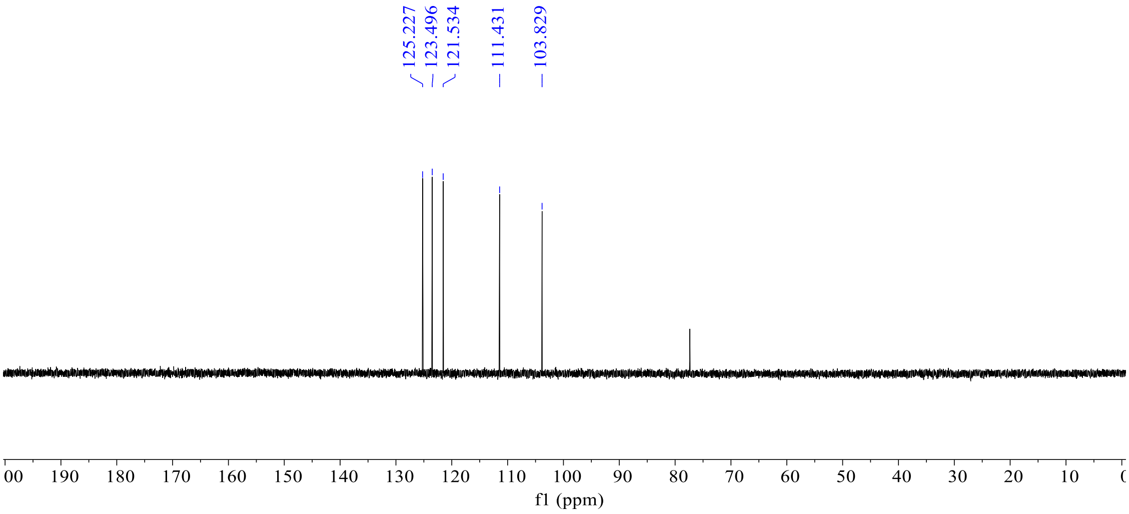

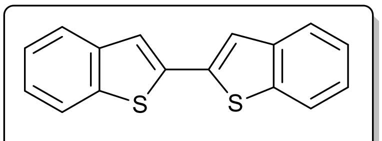

4o

^{1}H-NMR in $CDCl_3$

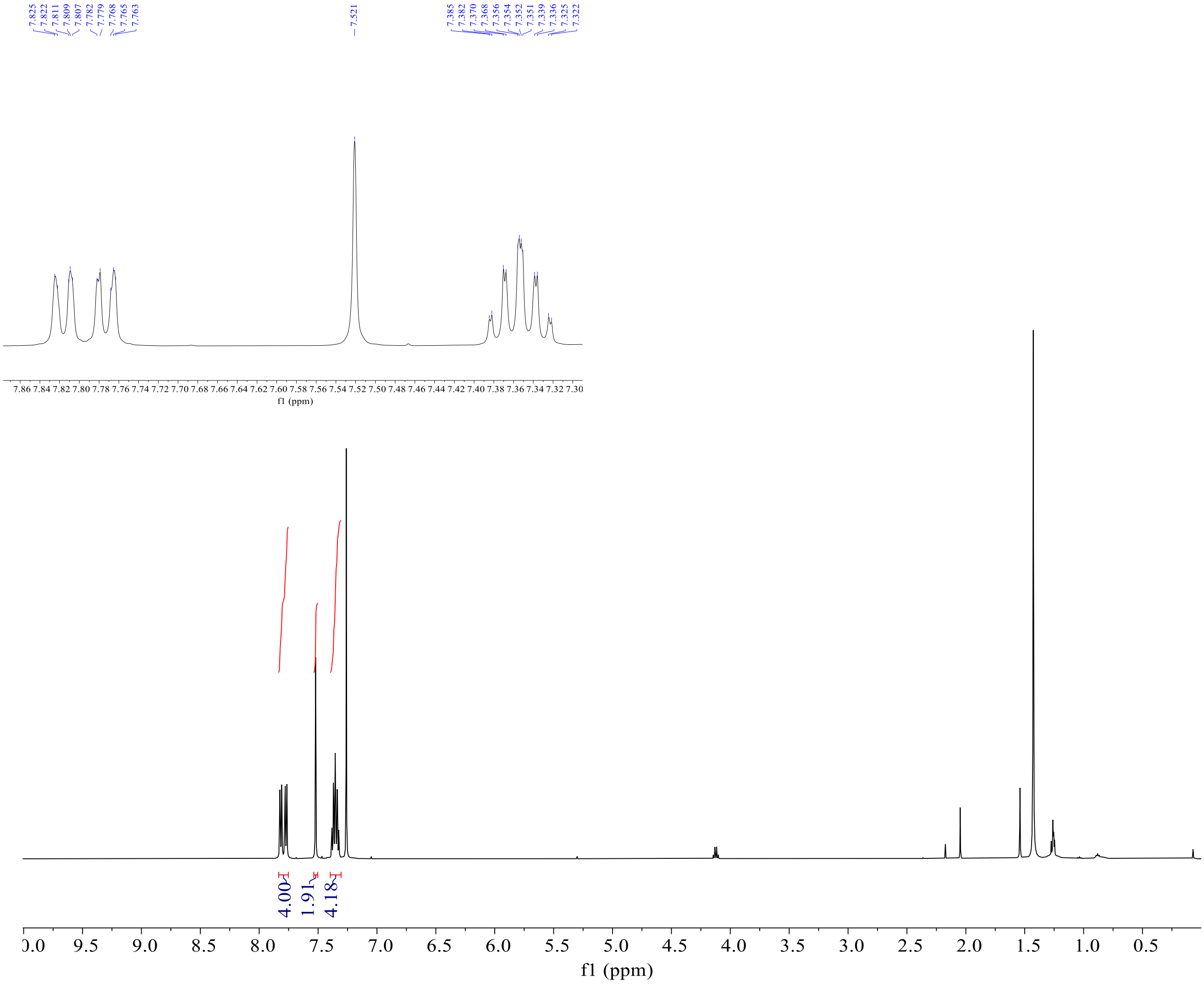

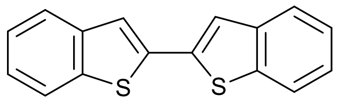

4o

^{1}H-NMR in $CDCl_3$ – list of peaks

Label	Intensity	Frequency(ppm)	Frequency(ppm)
1	5432	7.825	3913.521
2	2701	7.822	3912.081
3	3301	7.811	3906.435
4	3495	7.809	3905.638
5	4614	7.807	3904.479
6	4702	7.782	3892.134
7	5925	7.779	3890.483
8	3499	7.768	3885.086
9	4385	7.765	3883.721
10	4098	7.763	3882.654
11	19219	7.521	3761.476
12	1835	7.385	3693.275
13	2370	7.382	3691.955
14	5873	7.370	3686.115
15	5400	7.368	3684.721
16	6668	7.356	3678.839
17	4549	7.354	3678.011
18	4774	7.352	3677.095
19	6129	7.351	3676.235
20	5587	7.339	3670.366
21	4569	7.336	3668.855
22	2069	7.325	3663.210
23	1654	7.322	3661.712
24	39435	7.260	3630.967

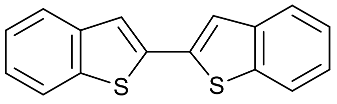

4o

^{13}C NMR in CDCl$_3$

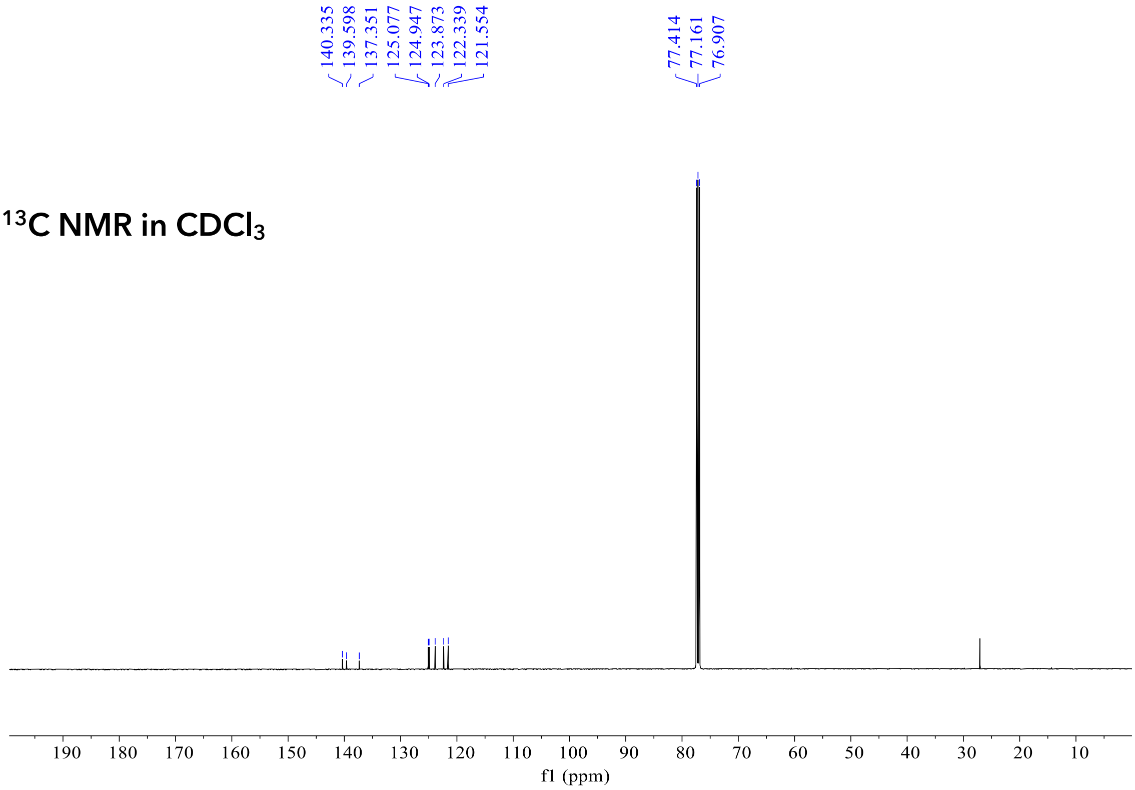

DEPT135 in CDCl$_3$

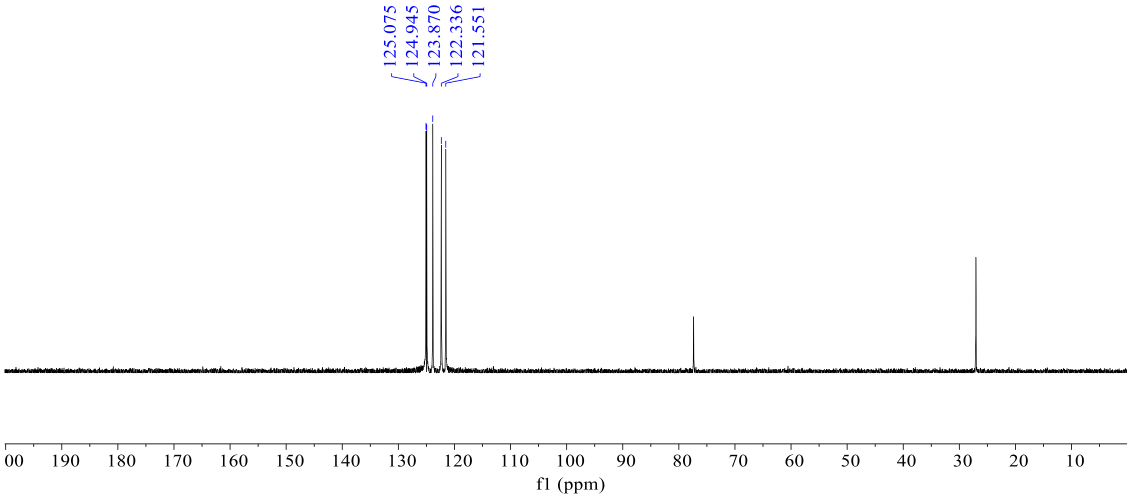

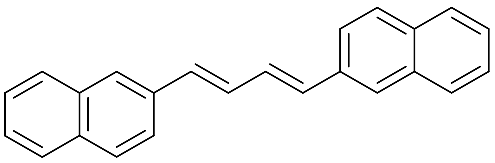

4q

^{1}H-NMR in $CDCl_3$

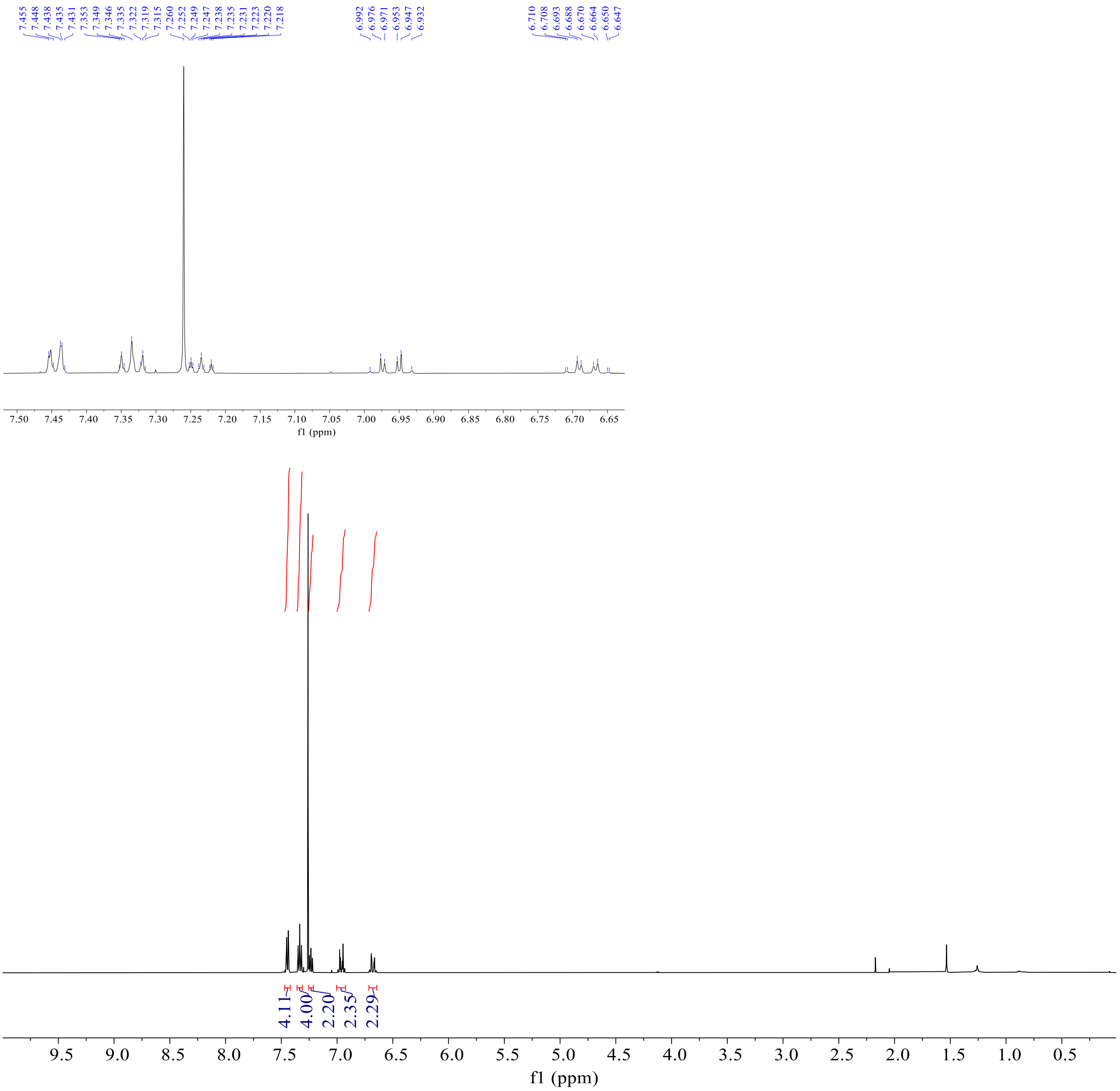

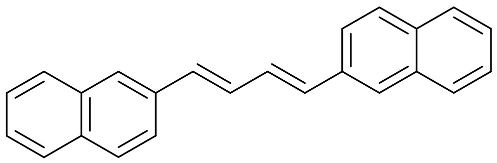

4q

^{1}H-NMR in $CDCl_3$ – list of peaks

Label	Intensity	Frequency(ppm)	Frequency(ppm)
1	402278	7.455	3728.264
2	112984	7.448	3724.781
3	657982	7.438	3719.805
4	623788	7.435	3718.644
5	41568	7.431	3716.654
6	86558	7.353	3677.346
7	418137	7.349	3675.687
8	118546	7.346	3673.863
9	773886	7.335	3668.390
10	163376	7.322	3662.087
11	439251	7.319	3660.428
12	48104	7.315	3658.604
13	7450069	7.260	3630.949
14	132052	7.252	3626.964
15	261053	7.249	3625.679
16	117293	7.247	3624.420
17	87418	7.238	3620.149
18	413387	7.235	3618.338
19	67428	7.231	3616.470
20	81431	7.223	3612.249
21	227059	7.220	3611.114
22	67652	7.218	3609.858
23	49735	6.992	3496.728
24	380603	6.976	3489.113
25	251340	6.971	3486.300
26	309547	6.953	3477.227
27	472676	6.947	3474.402
28	74197	6.932	3466.749
29	33818	6.710	3356.044
30	45222	6.708	3354.623
31	318070	6.693	3347.620
32	204089	6.688	3344.794
33	180471	6.670	3335.806
34	252102	6.664	3332.862
35	30794	6.650	3325.806
36	23825	6.647	3324.569

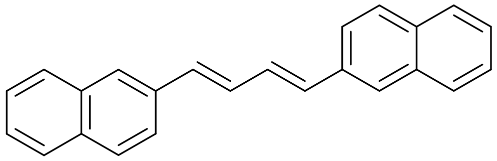

4q

^{13}C NMR in $CDCl_3$

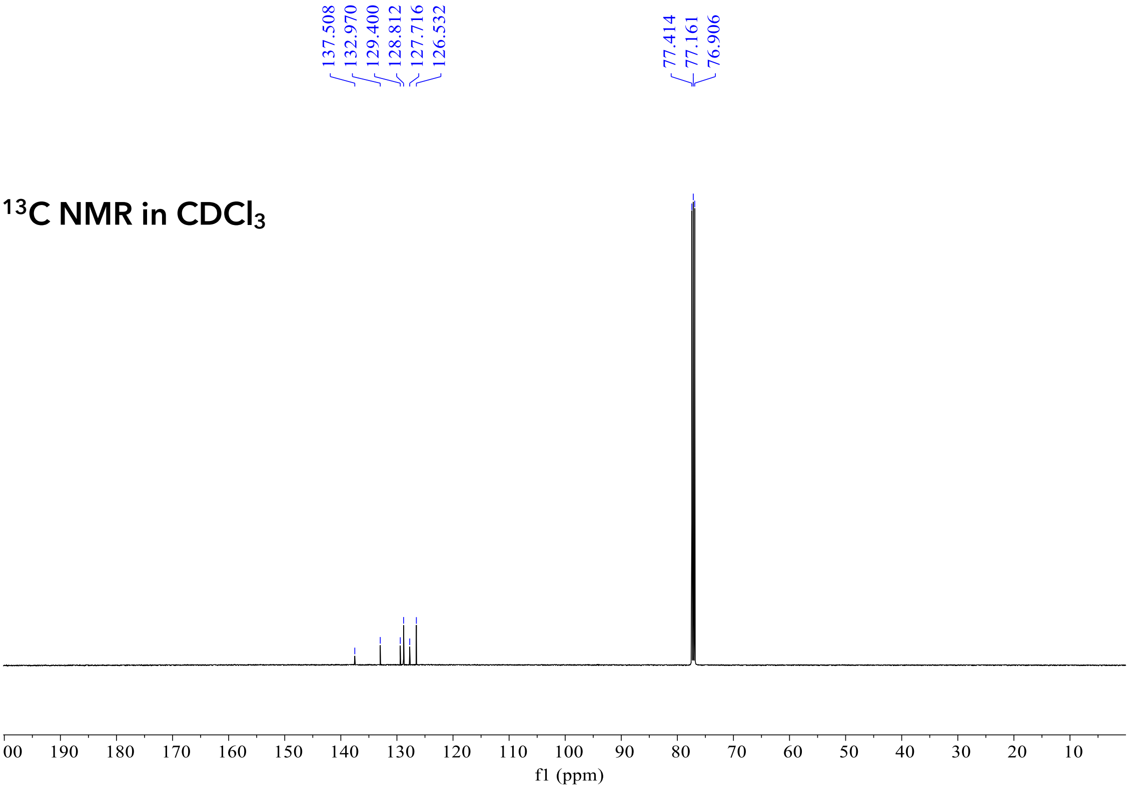

DEPT135 in $CDCl_3$

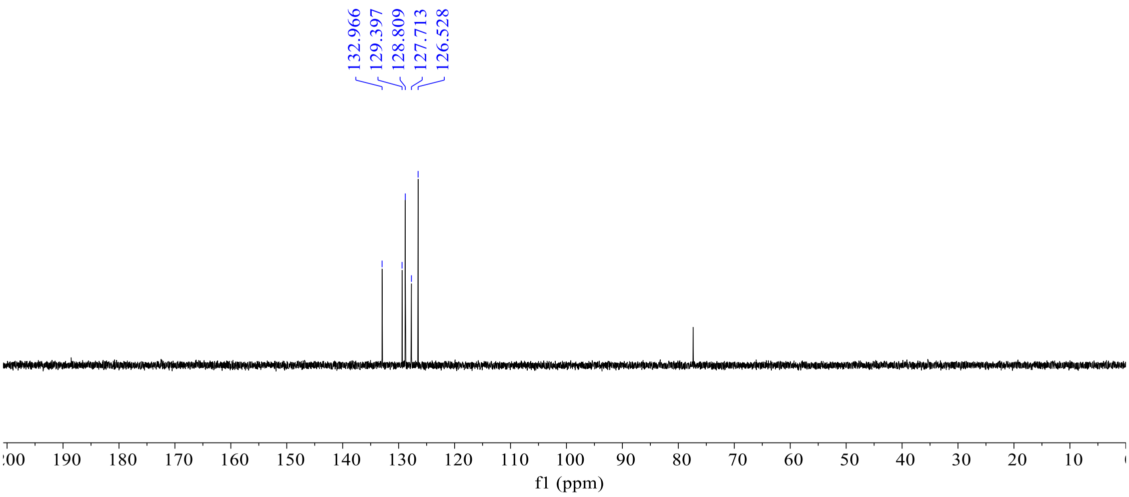

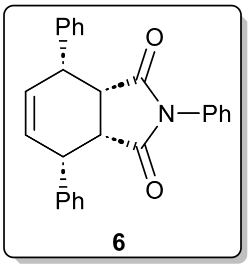

6

^{1}H-NMR in CDCl$_3$

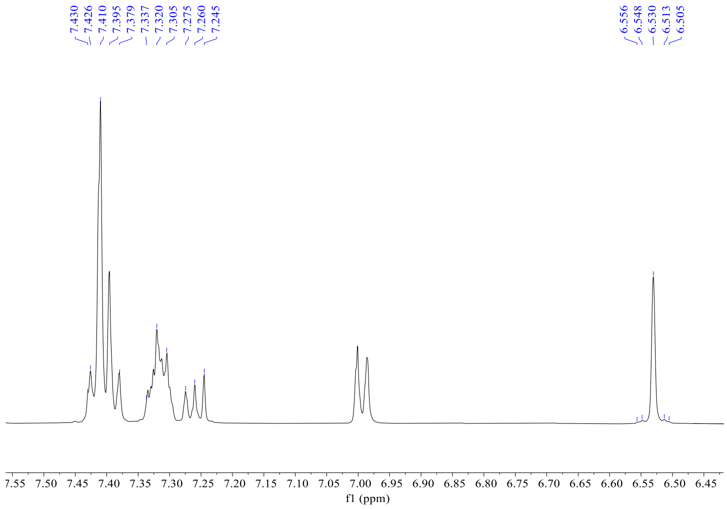

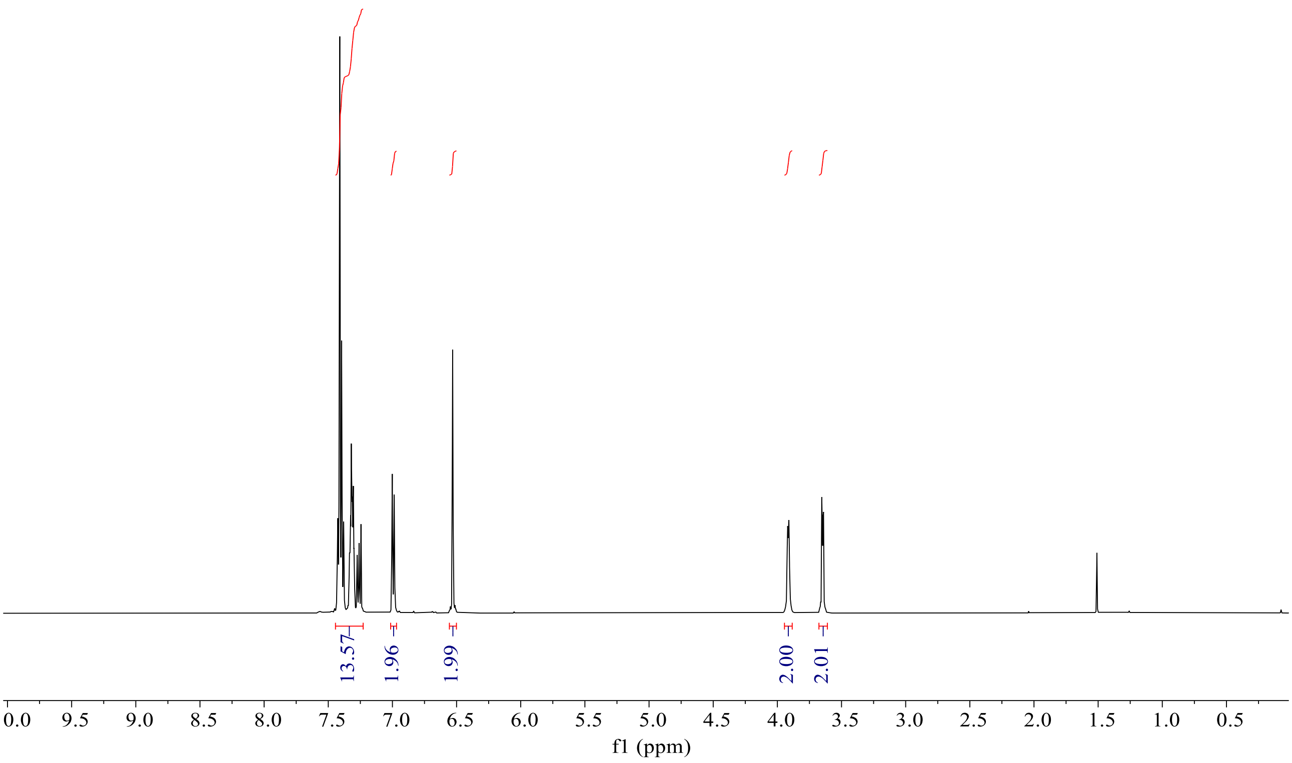

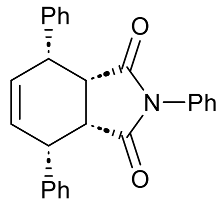

6

^{1}H-NMR in $CDCl_3$ – list of peaks

Label	Intensity	Frequency(ppm)	Frequency(ppm)
1	84	7.430	3716.492
2	212	7.426	3714.288
3	1670	7.410	3706.235
4	681	7.395	3699.030
5	223	7.379	3691.061
6	58	7.337	3669.699
7	400	7.320	3661.476
8	248	7.305	3653.592
9	154	7.275	3638.588
10	207	7.260	3631.286
11	253	7.245	3623.753
12	4	6.556	3279.328
13	14	6.548	3275.344
14	804	6.530	3266.401
15	15	6.513	3257.712
16	6	6.505	3253.812
17	109	3.922	1961.931
18	178	3.919	1960.039
19	170	3.911	1956.122
20	121	3.907	1954.391
21	304	3.654	1827.701
22	219	3.650	1825.478
23	237	3.644	1822.697
24	243	3.640	1820.598

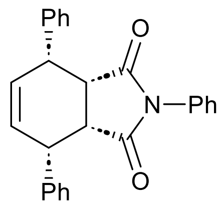

6

^{13}C NMR in $CDCl_3$

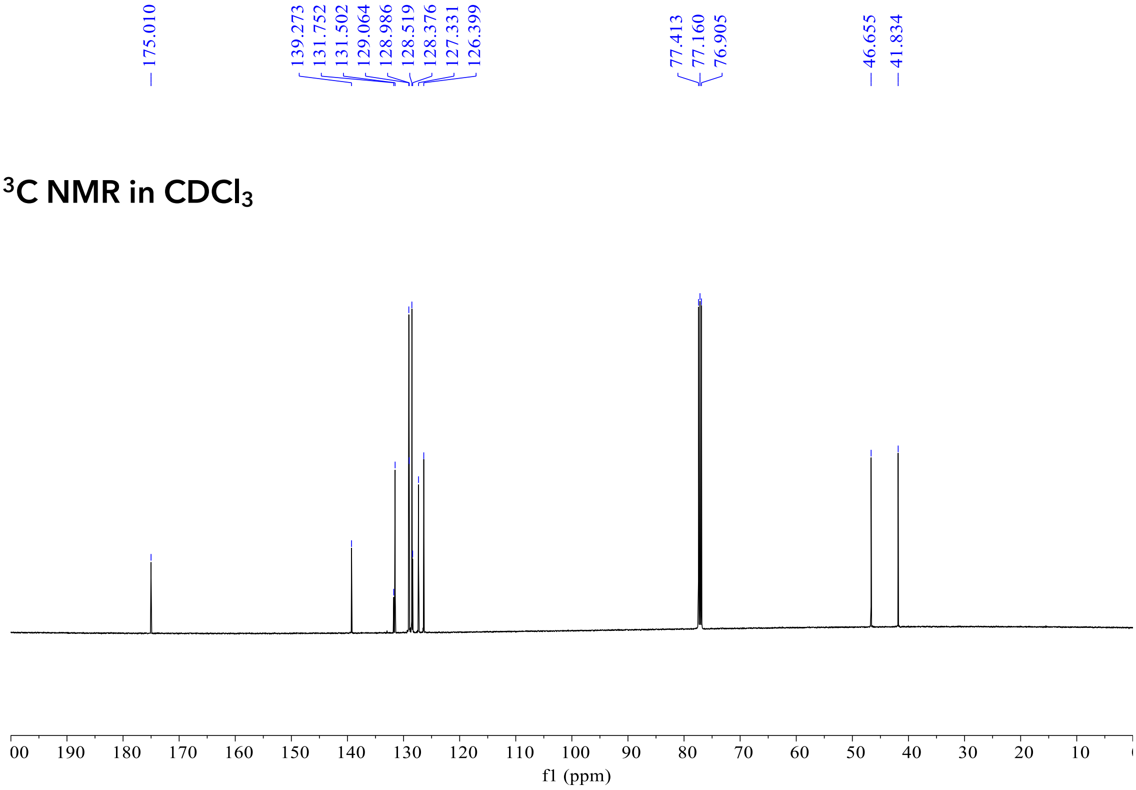

DEPT135 in $CDCl_3$

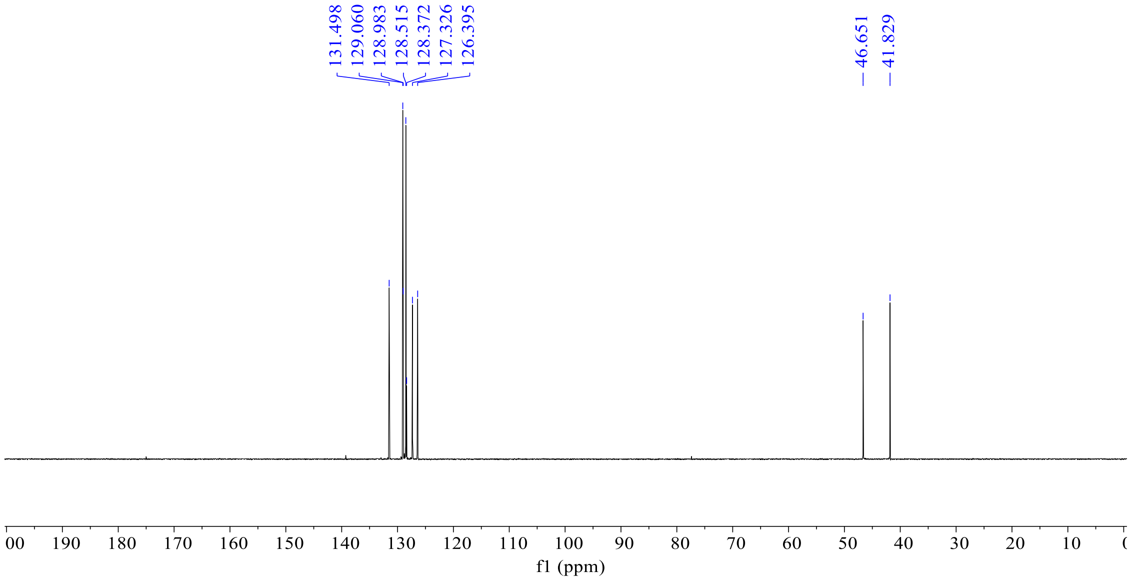

Supplement: Supplementary file 1 [file molecules-29-05552-s001.zip › molecules-3313609-supplementary.pdf]
